# Proteomic profiling reveals CEACAM6 function in driving gallbladder cancer aggressiveness through integrin receptor, PRKCD and AKT/ERK signaling

**DOI:** 10.1038/s41419-024-07171-x

**Published:** 2024-10-28

**Authors:** Raisatun Nisa Sugiyanto, Carmen Metzger, Aslihan Inal, Felicia Truckenmueller, Kira Gür, Eva Eiteneuer, Thorben Huth, Angelika Fraas, Ivonne Heinze, Joanna Kirkpatrick, Carsten Sticht, Thomas Albrecht, Benjamin Goeppert, Tanja Poth, Stefan Pusch, Arianeb Mehrabi, Peter Schirmacher, Junfang Ji, Alessandro Ori, Stephanie Roessler

**Affiliations:** 1 Institute of Pathology, University Hospital Heidelberg, Heidelberg University, Heidelberg, Germany; 2 Leibniz Institute on Aging-Fritz Lipmann Institute (FLI), Jena, Germany; 3 NGS Core Facility, Medical Faculty Mannheim, Heidelberg University, Mannheim, Germany; 4 Liver Cancer Centre Heidelberg (LCCH), Heidelberg, Germany; 5 Institute of Tissue Medicine and Pathology, University of Bern, Bern, Switzerland; 6 Institute of Pathology and Neuropathology, RKH Hospital Ludwigsburg, Ludwigsburg, Germany; 7 Center for Model System and Comparative Pathology, Institute of Pathology, University Hospital Heidelberg, Heidelberg University, Heidelberg, Germany; 8 Clinical Cooperation Unit Neuropathology, German Cancer Research Center (DKFZ), Heidelberg, Germany; 9 Department of Neuropathology, Institute of Pathology, University Hospital Heidelberg, Heidelberg, Germany; 10 Department of General Visceral and Transplantation Surgery, University Hospital Heidelberg, Heidelberg, Germany; 11 The MOE Key Laboratory of Biosystems Homeostasis & Protection, Zhejiang Provincial Key Laboratory for Cancer Molecular Cell Biology, Life Sciences Institute, Zhejiang University, Hangzhou, Zhejiang China

**Keywords:** Translational research, Biliary tract cancer, Proteomics, Protein-protein interaction networks, Preclinical research

## Abstract

Gallbladder cancer (GBC) presents as an aggressive malignancy with poor patient outcome. Like other epithelial cancers, the mechanisms of GBC cancer progression remain vague and efforts in finding targeted therapies fall below expectations. This study combined proteomic analysis of formalin-fixed paraffin-embedded (FFPE) GBC samples, functional and molecular characterization of potential oncogenes and identification of potential therapeutic strategies for GBC. We identified Carcinoembryonic Antigen-related Cell Adhesion Molecule 6 (CEACAM6) as one of the significantly most upregulated proteins in GBC. CEACAM6 overexpression has been observed in other cancer entities but the molecular function remains unclear. Our functional analyses in vitro and in vivo mouse models revealed that CEACAM6 supported the initial steps of cancer progression and metastasis by decreasing cell adhesion and promoting migration and invasion of GBC cells. Conversely, CEACAM6 knockdown abolished GBC aggressiveness by increasing cell adhesion while reducing cell migration, cell proliferation, and colony formation. BirA-BioID followed by mass-spectrometry revealed Integrin Beta-1 (ITGB1) and Protein Kinase C Delta (PRKCD) as direct molecular and functional partners of CEACAM6 supporting GBC cell migration. ERK and AKT signaling and their downstream target genes were regulated by CEACAM6 and thus the treatment with AKT inhibitor capivasertib or ERK inhibitor ulixertinib mitigated the CEACAM6-induced migration. These findings demonstrate that CEACAM6 is crucially involved in gallbladder cancer progression by promoting migration and inhibiting cell adhesion through ERK and AKT signaling providing specific options for treatment of CEACAM6-positive cancers.

## Introduction

Gallbladder cancer (GBC) is the most common biliary tract cancer (BTC) and the sixth most common malignancy of the gastrointestinal tract [1]. Although GBC is classified as a rare cancer, there are distinct differences in GBC around the globe reaching epidemic levels in some regions and ethnicities [2]. The highest incidence rates are found in South America, northern India, and East Asia [3]. GBC 5-year survival rate is less than 20% due to diagnosis at progressed state and resistance to chemotherapy [2]. Less than 10% of patients with GBC are resectable [4] and more than 40% are diagnosed after cancer cells have spread to regional lymph nodes or the liver [5]. This late diagnosis and the resistance of GBC to standard chemotherapy accounts for the poor prognosis of patients with GBC [3, 6].

The mechanisms of GBC metastasis and aggressiveness are largely unknown and understudied. The molecular signaling cascades involved in GBC progression are still unknown. Often patients with GBC are treated with the same therapy as other BTC diseases such as extrahepatic cholangiocarcinoma (eCCA), even though there is growing evidence of significantly different molecular profiles of GBC and eCCA [7]. Thus, efforts in finding the potential targets and molecular drivers of GBC are urgently needed and critical for the development of effective treatment of GBC.

Fresh frozen tissues are generally preferable for protein analysis, yet in a rare disease such as GBC, the availability of such specimens is a major drawback. Formalin-fixed paraffin-embedded (FFPE) tissues are more cost-effective and widely available and new technologies now enable untargeted quantitative proteomics of FFPE tissues [8–10]. In addition, the recently established protein isolation protocol used in this study facilitates quantitative proteomic analysis despite the limited amounts of FFPE material [9]. Consequently, the proteomics data included in this study unveiled valuable insights into deregulated proteins in GBC.

Carcinoembryonic Antigen-related Cell Adhesion Molecule 6 (CEACAM6), which emerged as one of the highest and most significantly upregulated proteins in our GBC patient cohort, also referred to as CD66c, is one of the CEACAM family members [11]. Its overexpression correlated with reduced overall survival in gastric cancer [12], pancreatic ductal carcinoma [13], osteosarcoma [14], and oral squamous cell carcinoma [15]. An oncogenic role of CEACAM6 has been suggested in breast, thyroid, colon, and pancreas carcinoma [16]. Thereby, CEACAM6 promoted cancer metastasis by increasing cell migration and invasion abilities [17]. However, the role and molecular mechanism of CEACAM6 in GBC progression remained unclear. Here, we identified CEACAM6 as a key factor in supporting GBC aggressiveness in vitro and in vivo. We showed that CEACAM6 directly interacted with Integrin Beta-1 (ITGB1) and Protein Kinase C Delta (PRKCD) mediating CEACAM6-induced GBC cell migration. Furthermore, inhibition of AKT and ERK as key nodes in CEACAM6 downstream signaling pathways suppressed GBC cell migration. Thus, this study provides comprehensive data on CEACAM6 molecular, functional, and translational properties in the context of GBC.

## Materials and methods

### Patient samples

The FFPE tissue blocks of GBC tumor tissues and non-tumorous normal gallbladder tissues were provided by the Tissue Bank of the National Center for Tumor Diseases (NCT, Heidelberg, Germany). GBC cases were histologically confirmed by at least two board-certified pathologists of the Institute of Pathology at University Hospital Heidelberg. The control tissues of non-tumor (NT) gallbladder were obtained from patients who underwent cholecystectomy because of gallstone disease but were only included in the study if the tissue was not strongly inflamed.

### Ethics approval and consent to participate

All research was conducted in accordance with the Declarations of Helsinki and Istanbul. The project was approved by the ethics committee of Heidelberg University (approval codes S-206/2005 and S-519/2019) The study was exempt from informed consent by the subjects.

### Mass spectrometry from FFPE tissue

Protein samples for mass spectrometry analysis were prepared and isolated from FFPE tissue blocks of five GBC and five NT gallbladders based on the previously described protocol [9]. The isolated peptides were labeled with the isobaric mass tags also referred to as TMT-10plexing and were performed as described previously. The TMT-labelled samples were subjected to high-pH liquid chromatography and thereby fractionated and injected afterward into the mass spectrometer.

### Statistical analyses

Statistical analyses were performed using GraphPad Prism 8.3.1 for Windows software (GraphPad Software, La Jolla, CA, USA). Data are presented as mean ± SD, as indicated. For statistical analyses, chi-square test, ANOVA test for multiple comparisons, or Student’s *t*-test for comparison of two groups were used. *P* values below 0.05 were considered statistically significant.

### Bioinformatic analyses

Bioinformatic analysis was done using statistical computing environment R (version 4.0.4, http://www.R-project.org/). Gene expression data were plotted using ggplot2 packages. Statistical analysis between two groups of RNAseq gene expression data was performed using the limma package in R.

Additional “Materials and methods” are provided online in the Supplementary Data.

## Results

### CEACAM6 is upregulated in gallbladder cancer tissue

**Fig. 1:**
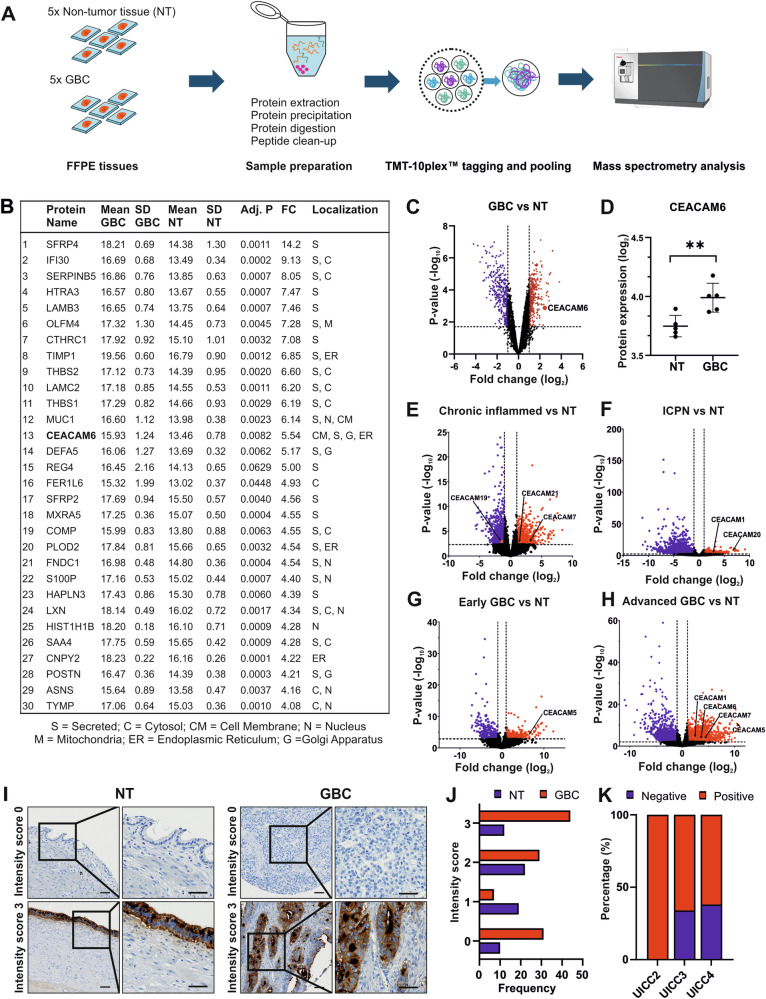
Proteomic profiling of gallbladder cancer samples. **A** Study design of mass spectrometry (MS) analysis. **B** Table showing top 30 most upregulated proteins in GBC compared to NT with its individual expression, standard deviation (SD) among samples, adjusted *p* value (Adj. P), fold-change (FC), and subcellular localization. **C** Volcano plot showing the protein expression of GBC versus gallbladder non-tumor tissue (NT). CEACAM6 is highlighted. **D** Dot plot of individual measurements of CEACAM6 protein expression based on MS analysis. **E** Volcano plots of the GSE202479 dataset comparing patient samples of the gallbladder with chronic inflammation, **F** intracholecystic papillary neoplasm (ICPN, previously named adenoma), **G** early GBC or **H** advanced GBC versus NT. **I** Representative images of CEACAM6 staining in NT and GBC tissue samples with respective intensity score in tissue microarray (TMA) analysis. The scale bar corresponds to 50 µm. **J** Frequency of NT (*N* = 63) or GBC (*N* = 111) tissue samples with corresponding intensity score of CEACAM6 staining in TMA analysis shown in a bar graph. The frequency distribution was analyzed with chi-square test with χ^2^ = 24.136, df = 3, and *p* < 0.001. **K** Bar graph depicting percentage of CEACAM6 staining in GBC tissue samples for each GBC stage, UICC2 (*N* = 13), UICC3 (*N* = 56), and UICC4 (*N* = 21). Chi-square test revealed χ^2^ = 6.638, df = 2 and *p* = 0.0362.

To investigate the proteomic changes between GBC and NT gallbladder tissue, we compared five GBC and five NT tissue samples by quantitative mass-spectrometric analysis using Tandem Mass Tag (TMT) labeling (Fig. 1A and Table S1). The correlation plot and t-distributed Stochastic Neighbor Embedding (t-SNE) analysis showed that samples clustered based on the tissue type (Fig. S1A, B). A total of 4827 proteins were detected of which 719 were significantly different between GBC and NT tissues (fold change (FC) > |2|, adj. *p* ≤ 0.05, Table S2). Of the significantly different proteins, 348 proteins were downregulated and 371 proteins were upregulated. To select candidate proteins for functional analysis, we focused on upregulated proteins located in the cell membrane and excluded proteins which are mainly secreted as these are more difficult to target therapeutically. Based on the UniProtKB database (https://www.uniprot.org/uniprotkb), CEACAM6 and Mucin 1 (MUC1) were among the most overexpressed proteins located in the cell membrane (Fig. 1B–D and Table S2, FC = 5.54, adj. *p* ≤ 0.01). As CEACAM6 has been less studied in cancer compared to MUC1, we chose CEACAM6 for further studies. Next, the 719 significantly different proteins were subjected to Ingenuity Pathway Analysis (IPA) to reveal pathways involved in GBC tumorigenesis. Several signaling pathways related to metastasis, including mTOR signaling, regulation of actin-based motility by Rho, and remodeling of epithelial adherens junctions, were activated in GBC (Fig. S1C and Table S3) [18–20].

Considering that CEACAM6 is one out of 12 members of the CEACAM protein family, we also analyzed the expression of other CEACAM family members. In our GBC proteomics cohort besides CEACAM6, CEACAM5, CEACAM7, and CEACAM8 were detected but not significantly altered in GBC compared to NT samples (Fig S1D, E and Table S2). We also found that in a patient cohort from Henan Provincial People’s Hospital, which included multi-stage transcriptomic profiling of GBC transformation (GSE202479) [21], the *CEACAM6* mRNA was exclusively overexpressed in advanced GBC, while other CEACAM family members were found to be perturbed in early stages of GBC development (Fig. 1E–H and Table S4). In eCCA of an international multicenter cohort (GSE132305) [22], *CEACAM6* and *CEACAM5* were significantly upregulated compared to NT tissue, while *CEACAM7* was significantly downregulated (Fig. S1F and Table S4).

Next, we evaluated CEACAM6 expression using immunohistochemical staining of tissue microarrays (TMA) including NT (*N* = 63) and GBC (*N* = 111) tissue samples. CEACAM6 exhibited low (score 1, *N* = 19, 30.2%) or intermediate expression (score 2, *N* = 22, 34.9%) in most NT samples, whereas, GBC epithelium showed high expression (score 3, *N* = 44, 39.6%) in a large proportion of cases (chi-square test *p* < 0.001, Fig. 1I, J). Interestingly, in patients with low-stage GBC with UICC2, the tumor epithelium was positive in all cases, whereas in later-stage GBC, denoted by UICC3 and UICC4, the GBC epithelium was negative in more than one-third of patients suggesting that CEACAM6 is early in carcinogenesis upregulated but not required or even has negative effects in advanced stage GBC (Fig. 1K). However, CEACAM6 levels in GBC were not associated with overall patient survival (Fig. S1G). Thus, our and external cohort data consistently showed that CEACAM6 is upregulated in a subset of GBC suggesting an important functional role in the progression of GBC.

### CEACAM6 knockdown suppresses GBC aggressiveness

**Fig. 2:**
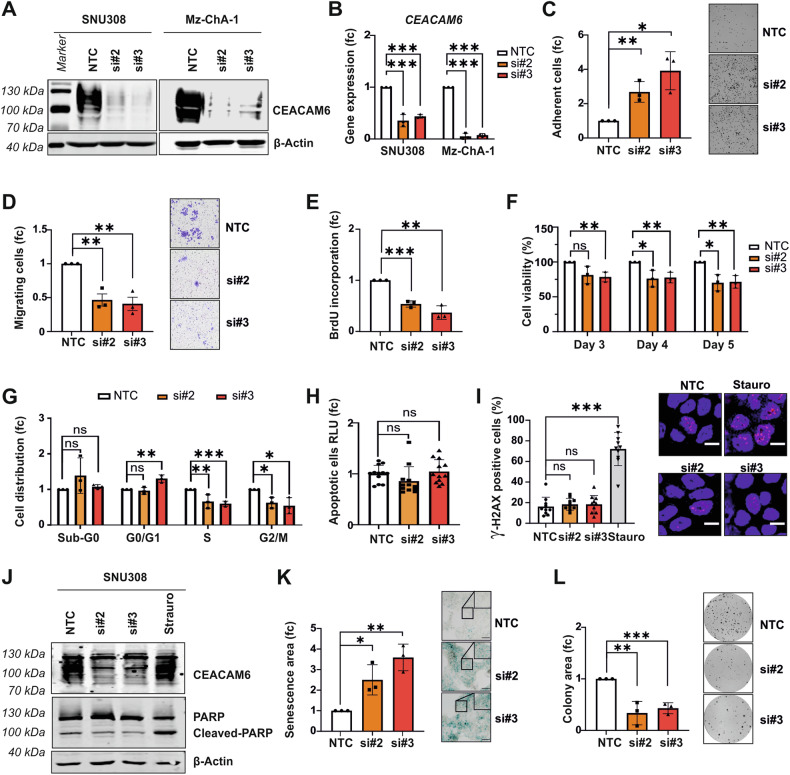
CEACAM6 knockdown inhibits GBC oncogenic phenotype. **A** CEACAM6 knockdown using two siRNAs targeting CEACAM6 (si#2 and si#3) transiently transfected in SNU308 and Mz-ChA-1 are shown in Western blot. β-Actin served as loading control. **B** qPCR was performed to analyze CEACAM6 gene expression levels on SNU308 and Mz-ChA-1 cells. SRSF4 gene expression was used as internal control for normalization. **C** Three days after transfection, cells were used for adhesion assay and images of adherent SNU308 cells 1 h after seeding were captured and quantified. **D** Transwell migration assay of SNU308 cells was quantified by the area of migrated cells per image and representative images of 10× microscope magnification are shown. Mean results of three independent replicates are shown. **E** Bar graphs showing relative cell proliferation based on BrdU incorporation ELISA and **F** cell viability of SNU308 cells after 3, 4, and 5 days of CEACAM6 knockdown. **G** Cell cycle distribution in sub-G0, G0/G1, S, and G2/M phases of SNU308 cells after CEACAM6 knockdown is depicted. **H** Relative light unit (RLU) of Annexin V apoptosis assay after CEACAM6 knockdown in SNU308 cells. **I** Percentage of γ-H2AX positive cells and representative immunofluorescence images of SNU308 after CEACAM6 knockdown. The scale is 10 µm. Staurosporine (25 µM) treatment for two hours was used as positive control. **J** Western blot image of CEACAM6 and PARP protein after CEACAM6 knockdown or after two hours of Staurosporine (25 µM) treatment. β-Actin served as loading control. **K** The senescent cell area in SNU308 cells was captured based on β-galactosidase staining with ×10 magnification. Representative images are shown of three independent replicates. Inset pictures show a detailed area and the scale bar is 20 µm. **L** Quantification and representative images of colony area after 14 days of CEACAM6 knockdown in SNU308 cells. Data are represented as mean ± SD of three independent experiments. *P* values were determined by unpaired *t*-test (*p* ≥ 0.05 ns, <0.05 *, <0.01 **, <0.001 ***).

To elucidate CEACAM6’s oncogenic function in vitro, we examined the expression levels of endogenous CEACAM6 proteins across various GBC cell lines (Fig. S1H). We conducted knockdown experiments using two siRNAs targeting CEACAM6 (siCEACAM6#2 and siCEACAM6#3) in the GBC cell lines SNU308 and Mz-ChA-1 to explore possible protumorigenic effects of CEACAM6 (Fig. 2A, B). Cell adhesion was significantly increased in both GBC cell lines upon siRNA-mediated CEACAM6 knockdown compared to non-targeting control (NTC, Figs. 2C and S2A). Conversely, cell migration was inhibited after CEACAM6 knockdown (Figs. 2D and S2B). We observed a reduction of cell proliferation based on BrdU assay (Figs. 2E and S2C) and cell viability by CEACAM6 knockdown compared to NTC in both GBC cell lines (Figs. 2F and S2D). Cell cycle analyses indicated that the reduction of cell viability is caused by cell cycle arrest in G1, significantly reducing cell proportions in S and G2/M phases (Figs. 2G and S2E). However, CEACAM6 knockdown did not induce apoptosis based on Annexin V assay (Figs. 2H and S2F). Further analyses of DNA damage marker γ-H2AX by immunofluorescence showed no differences after CEACAM6 knockdown (Figs. 2I and S2G). In contrast to Staurosporine control, CEACAM6 knockdown did not induce PARP cleavage suggesting that CEACAM6 did not lead to apoptosis (Figs. 2J and S2H). Interestingly, β-galactosidase staining furthermore revealed an induction of cellular senescence independent of the p16 pathway as CEACAM6-knockdown did not result in p16 induction (Figs. 2K and S2I, J) [23]. In addition, knockdown of CEACAM6 resulted in a significant reduction of single-cell colony formation (Figs. 2L and S2K).

**Fig. 3:**
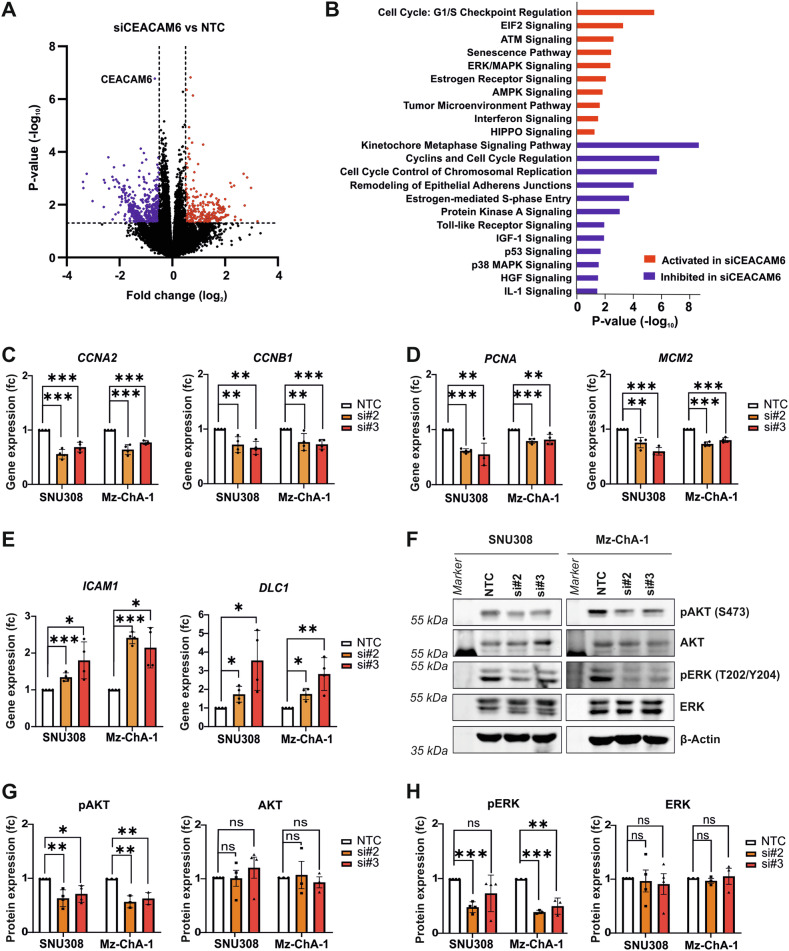
CEACAM6 knockdown inhibits ERK and AKT signaling. **A** Volcano plot of differentially expressed genes revealed by RNA sequencing of SNU308 cells after CEACAM6 knockdown using two siRNAs targeting CEACAM6 (si#2 and si#3) versus NTC and **B** the respective analysis of deregulated signaling pathways based on IPA. **C** qPCR analysis of genes related to cell cycle, **D** cell proliferation, and **E** senescence in SNU308 and Mz-ChA-1 cells after CEACAM6 knockdown (*N* = 4). SRSF4 gene expression was used as internal control for normalization. **F** Representative images of Western blots after CEACAM6 knockdown by transient transfection of two siRNAs showing protein levels of pAKT, AKT, pERK, and ERK in SNU308 and Mz-ChA-1 cells. β-Actin served as loading control. **G** Western blot signal quantification of pAKT and AKT and **H** of pERK and ERK in SNU308 (*N* = 4) and Mz-ChA-1 cells (*N* = 3). Respective band density was normalized to β-Actin. Data are represented as mean ± SD relative to NTC control. *P* values were determined by unpaired *t*-test (*p* ≥ 0.05 ns, <0.05 *, <0.01 **, <0.001 ***).

To identify the downstream signaling pathways affected by CEACAM6 inhibition, we performed RNA sequencing analysis of SNU308 cells after CEACAM6 knockdown with both siRNAs individually. The comparison of the gene expression profiles revealed that regulation of ERK/MAPK signaling, regulation of cell cycle, senescence pathways, and remodeling of epithelial adherens junctions were among the most significantly perturbed pathways based on IPA pathway analysis (Fig. 3A, B and Tables S5, S6). Independent qPCR validation of target genes known to regulate cell cycle and proliferation such as *CCNA2*, *CCNB1*, *PCNA,* and *MCM2* revealed significant reduction after CEACAM6 knockdown, while target genes related to senescence pathways such as *ICAM1* and *DLC1* were significantly increased in SNU308 and Mz-ChA-1 (Fig. 3C–E). We also found that AKT and ERK protein phosphorylation were significantly inhibited in both cell lines as a result of CEACAM6 knockdown (Fig. 3F–H). Overall, CEACAM6 inhibition was able to mitigate protumorigenic properties of GBC cells, possibly through ERK and AKT as downstream signaling pathways of CEACAM6.

### CEACAM6 regulates cell adhesion and the initial steps of cancer metastasis

**Fig. 4:**
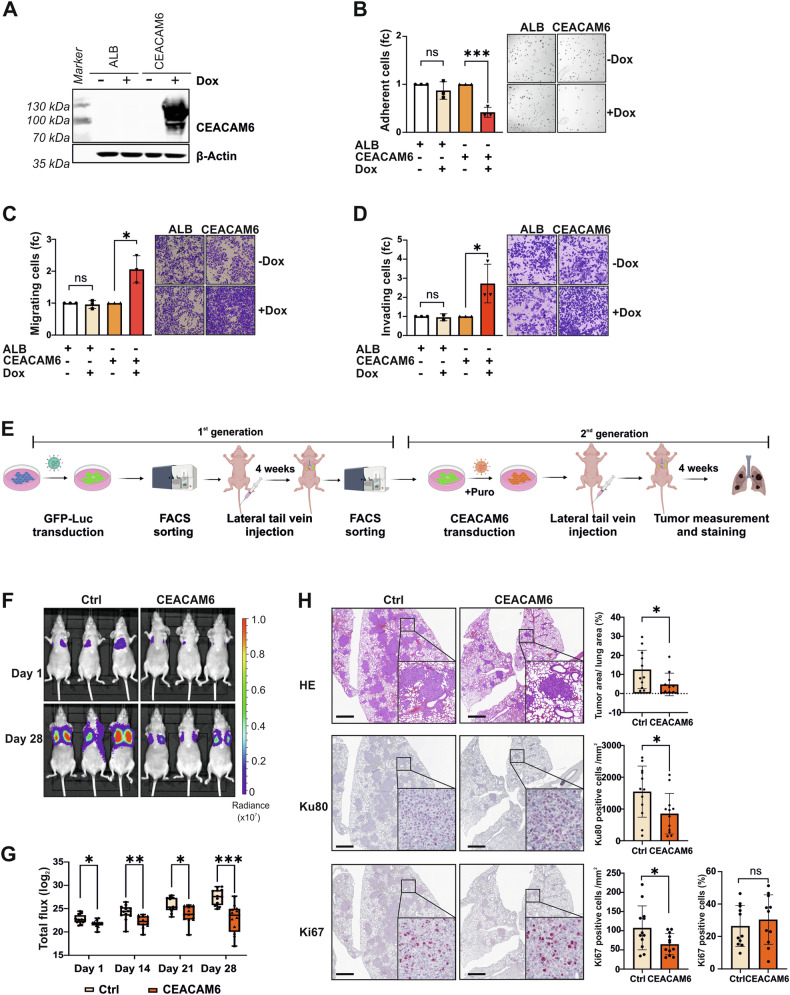
CEACAM6 regulates cell adhesion and the initial step of cancer metastasis. **A** Western blots of CEACAM6 overexpression in GB-d1 cells. ALB and CEACAM6 were overexpressed using inducible viral transduction with 2 μg/mL Doxycycline (Dox) treatment. Overexpression of ALB was used as a negative control. β-Actin served as loading control. **B** Quantification and images of GB-d1 adherent cells 1 h after seeding with or without ALB or CEACAM6 overexpression. **C** Transwell migration and **D** invasion assays of GB-d1 were quantified and representative ×10 magnification images are shown. **E** Study design of in vivo assay using lateral tail vein injection, created with Biorender.com. **F** Representative images of in vivo bioluminescence imaging on day 1 and day 28 after lateral tail vein injection. **G** Tumor growth in the lung was evaluated by total flux (log_2_) using the same region of interest (ROI) for every mouse and every measurement of mice injected with control (Ctrl, *N* = 11) or CEACAM6 (*N* = 11) expressing Gb-d1-GFP-Luc cells at different time points, as indicated. *P* values were determined by Mann-Whitney *U*-test (*p* ≥ 0.05 ns, <0.05 *, <0.01 **, <0.001 ***). **H** Representative images of lungs from the Ctrl and CEACAM6 group stained with HE, Ku80, or Ki67. Bar graphs indicating the percentage of tumor area based on HE staining, number of Ku80 positive cells per mm^2^, number of Ki67 positive cells per mm^2,^ and percentage of Ki67 positive cells. Each dot represents one mouse of the control (Ctrl, *N* = 11) or CEACAM6 (*N* = 11) group. Error bars depict SD and *p* values were determined by unpaired *t*-test (*p* ≥ 0.05 ns, <0.05 *, <0.01 **, <0.001 ***). The scale bar shown in the images is 100 μm.

Correspondingly, we established stable doxycycline (Dox)-inducible overexpression of CEACAM6 or albumin (ALB) as control. GB-d1 and TGBC1 cells were chosen for subsequent CEACAM6 overexpression studies as they possessed low expression of endogenous CEACAM6 (Fig. S1H). Successful overexpression of CEACAM6 or ALB was confirmed by Western blots and qPCR analysis (Figs. 4A and S3A–C). In both cell lines, overexpression of CEACAM6 inhibited cell adhesion (Figs. 4B and S3D) but increased cell migration (Figs. 4C, and S3E) and invasion (Figs. 4D and S3F). However, cell viability remained unchanged (Fig. S3G, H) excluding the possibility of increased migration due to cell proliferation.

To determine CEACAM6 function in vivo, we conducted lateral tail vein injection of GBC cells with or without CEACAM6 expression which were labeled with a GFP-Luc-fusion protein for in vivo monitoring (Fig. 4E). Stable overexpression of CEACAM6 protein was monitored by Western blot (Fig. S3I). In addition, FACS analysis and luciferase assay were performed to validate comparable GFP and luciferase expression between GFP-Luc-Ctrl and GFP-Luc-CEACAM6 (Fig. S3J, K). Tumor cell growth in the mouse lungs was monitored over a period of 4 weeks by bioluminescence imaging. Intriguingly, CEACAM6 overexpression caused less adhesion of tumor cells to the endothelium of pulmonary blood vessels starting the first day after tumor cell injection (Fig. 4F). Consistently, this effect led to reduced bioluminescence over the observation period of 4 weeks in mice injected with CEACAM6 expressing compared to control GBC cells (Fig. 4F, G). In addition, we observed a smaller tumor area derived from human GBC cells in mice injected with CEACAM6-expressing cells based on hematoxylin and eosin (HE) and Ku80 staining (Fig. 4H). The number of Ki67-positive cells per lung area was reduced in the CEACAM6 expressing group due to a lower number of tumor cells (Fig. 4H). However, within the tumor, there was no difference in cell proliferation marker Ki67 between tumors with or without CEACAM6, showing that CEACAM6 overexpression did not affect cell proliferation (Fig. 4H). Overall, the in vivo results were in accordance with the in vitro data showing that CEACAM6 decreased tumor cell adhesion to the pulmonal blood vessels but did not affect tumor cell viability. Thus, CEACAM6 demonstrated a pivotal role in supporting cell migration by regulating cell adhesion which may promote the initial steps of cancer metastasis in vivo and in vitro.

**Fig. 5:**
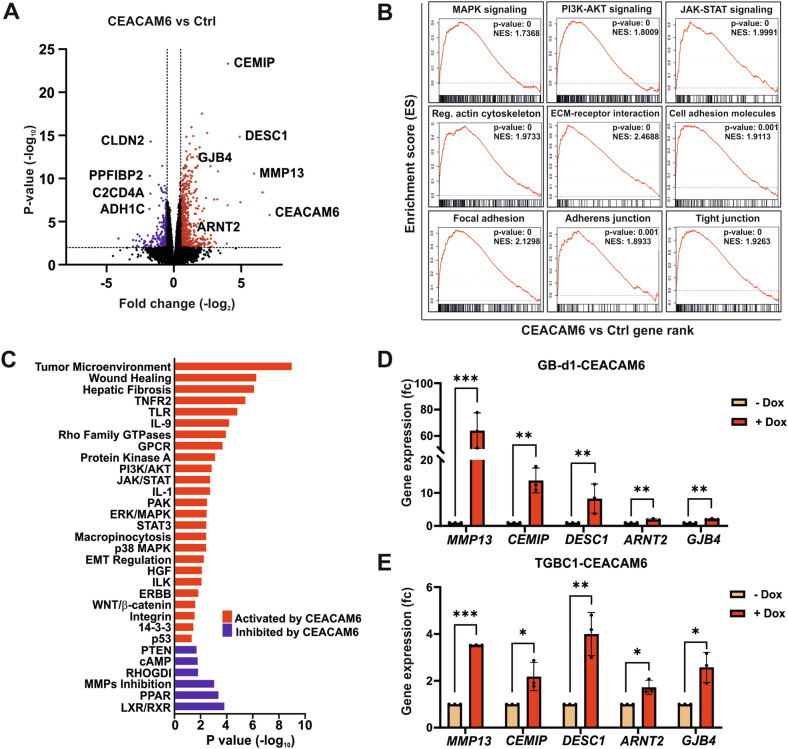
CEACAM6 regulates gene expression related to cell adhesion and migration. **A** Volcano plot of gene expression of GB-d1-CEACAM6 cells with or without CEACAM6 overexpression induced by Dox (2 µg/mL) based on RNA sequencing. **B** Gene Set Enrichment Analysis (GSEA) and **C** Ingenuity Pathways Analysis (IPA) displaying significant signaling pathways activated or inhibited by CEACAM6. **D** qPCR validation of *MMP13*, *CEMIP*, *DESC1*, *ARNT2,* and *GJB4* as upregulated genes regulated by CEACAM6 based on RNA sequencing analysis in GB-d1-CEACAM6 and **E** TGBC1-CEACAM6 cells with or without Dox (2 µg/mL) to induce CEACAM6 expression. *SRSF4* was used as internal control for gene normalization. Error bars depict SD and *p* values were determined by unpaired *t*-test (*p* ≥ 0.05 ns, <0.05 *, <0.01 **, <0.001 ***).

To gain better insight into the molecular perturbations caused by CEACAM6, we performed RNA sequencing of GB-d1 control or CEACAM6 expressing cells (Fig. 5A and Table S7). Gene set enrichment analysis (GSEA) and IPA revealed activation of pathways related to cell migration and tumor metastasis such as MAPK, PI3K/AKT, and JAK/STAT signaling. Increased signaling related to regulation of actin cytoskeleton, cell and focal adhesion, tumor microenvironment signaling, wound healing, and suppressed inhibition of MMPs in CEACAM6 expressing GB-d1 cells were also observed (Fig. 5B, C and Table S8). Among the significantly upregulated genes, we validated several genes related to cell migration and adhesion, including *MMP13*, *CEMIP*, *DESC1*, *ARNT2,* and gap junction beta-4 protein (*GJB4)* by qPCR in GB-d1 and TGBC1 (Fig. 5D, E). These findings further explained the relevance of CEACAM6 oncogenic properties in supporting cancer cell migration, adhesion, and metastasis.

### CEACAM6 directly interacts with ITGB1 and PRKCD to regulate cell migration

**Fig. 6:**
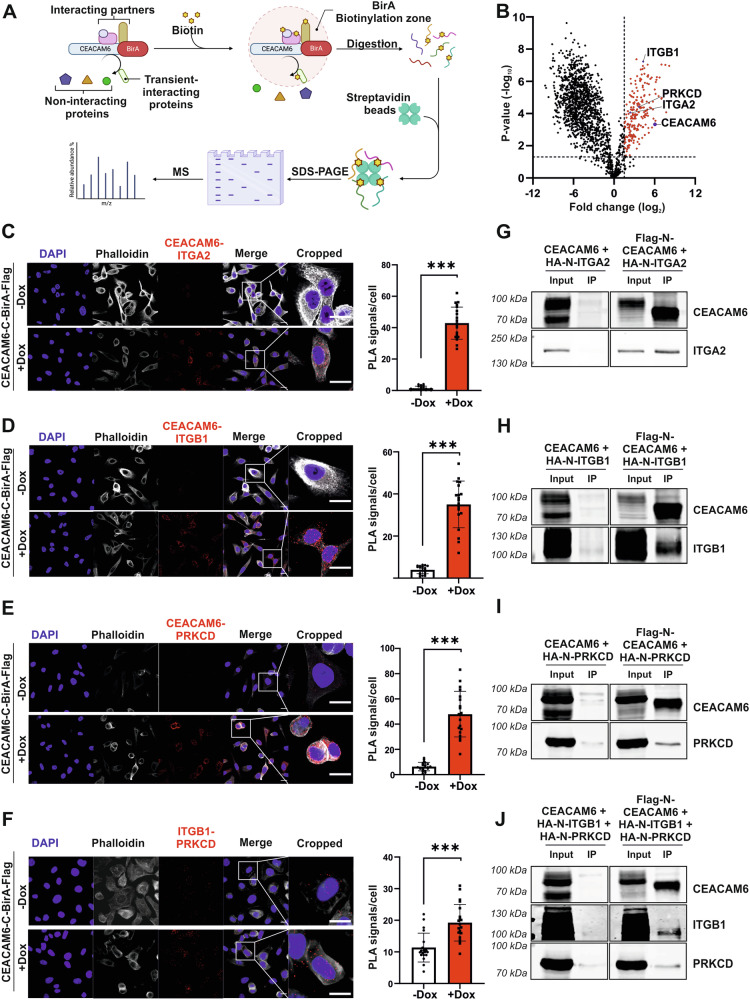
BirA-BioID and validation of CEACAM6 interacting proteins. **A** Overview of the BirA-BioID approach illustrated with Biorender.com. **B** Volcano plot of biotinylated proteins in CEACAM6-C-BirA-Flag versus Ctrl-BirA-Flag reveals Integrin Alpha-2 (ITGA2, FC = 5.23, adj. *p* < 0.01), Integrin Beta-1 (ITGB1, FC = 19.91, adj. *p* < 0.01) and Protein Kinase C Delta (PRKCD, FC = 12.94, adj. *p* < 0.01) as interacting partners of CEACAM6. **C** Proximity ligation assay (PLA) revealed direct interaction of CEACAM6 with ITGA2, **D** ITGB1, **E** PRKCD, and **F** interaction of ITGB1 with PRKCD. PLA images and quantification of PLA signals per cell of GB-d1-CEACAM6-C-BirA-Flag with or without Dox (2 µg/mL) treatment from two independent replicates and 10 different image areas with 60× microscope magnification each are displayed. Error bars depict SD and *p* values were determined by Mann-Whitney *U*-test (*p* < 0.001 ***). The scale bar in the PLA picture is 10 µm. **G** Co-immunoprecipitation (co-IP) experiments were performed in HEK cells with transient overexpression of CEACAM6 and its interacting partner proteins further validated the direct interaction of CEACAM6 with ITGA2, **H** ITGB1, **I** PRKCD and **J** ITGB1/PRKCD complex interaction.

To uncover the molecular mechanisms how CEACAM6 influences intracellular signaling leading to GBC cell metastasis, we performed proximity labeling by biotin ligase BirA (named BioID) to identify CEACAM6 interaction partners (Fig. 6A). This system results in the biotinylation of proteins within proximity of the BirA-fusion protein [24]. In this study, BirA-Flag was fused to the C-terminus of CEACAM6 to detect intracellular interaction partners. Inducible expression and correct localization of CEACAM6-C-BirA-Flag fusion or Ctrl-BirA-Flag protein were confirmed by immunofluorescence and Western blot (Fig. S4A, B). The addition of biotin led to biotinylation of proteins in the proximity of the CEACAM6-C-BirA-Flag protein and random biotinylation in Ctrl-BirA-Flag cells (Fig. S4C). Streptavidin-pulldown of biotinylated proteins followed by mass spectrometry revealed enrichment of potential CEACAM6 interaction partners (Fig. 6B and Table S9). In total, 224 proteins were significantly more abundant in the CEACAM6-C-BirA-Flag group compared to the control (FC > 3.0, adj. *p* ≤ 0.05, Table S9). Among those proteins, 189 proteins are predominantly located in the cell membrane, endoplasmic reticulum (ER), or Golgi apparatus. Consistently, we found that endogenous and overexpressed CEACAM6 localized to these subcellular locations (Fig. S5A–H). We further categorized the interaction partner candidates based on their gene ontology terms associated with cell migration, adhesion, and cell motility. Integrin Alpha-2 (ITGA2, FC = 5.23, adj. *p* < 0.01), Integrin Beta-1 (ITGB1, FC = 19.91, adj. *p* < 0.01) and Protein Kinase C Delta (PRKCD, FC = 12.94, adj. *p* < 0.01) were among the top enriched proteins and were found to co-localize with both endogenous and overexpressed CEACAM6 (Figs. 6B, S6A–H and Table S9).

We then validated the protein-protein interactions through proximity ligation assay (PLA) and co-immunoprecipitation (co-IP) experiments. Confocal imaging of PLA showed a significantly increased number of PLA interaction signals upon CEACAM6 induction for ITGA2 (Fig. 6C), ITGB1 (Fig. 6D), and PRKCD (Fig. 6E). We also found that interaction of endogenous ITGB1 and PRKCD protein was further enhanced by CEACAM6 overexpression shown by the increase of PLA signals (Fig. 6F). The CEACAM6-Flag pulldown in co-IP experiments demonstrated positive direct interactions between CEACAM6 and ITGA2 (Fig. 6G), ITGB1 (Fig. 6H) and PRKCD (Fig. 6I). Furthermore, ITGB1 and PRKCD were pulled down together with CEACAM6 in co-IP (Fig. 6J). The interaction between CEACAM6 with ITGA2, ITGB1, and PRKCD as well as between ITGB1 and PRKCD were also observed endogenously in SNU308 and Mz-ChA-1 cells by PLA (Fig. S7A–H).

**Fig. 7:**
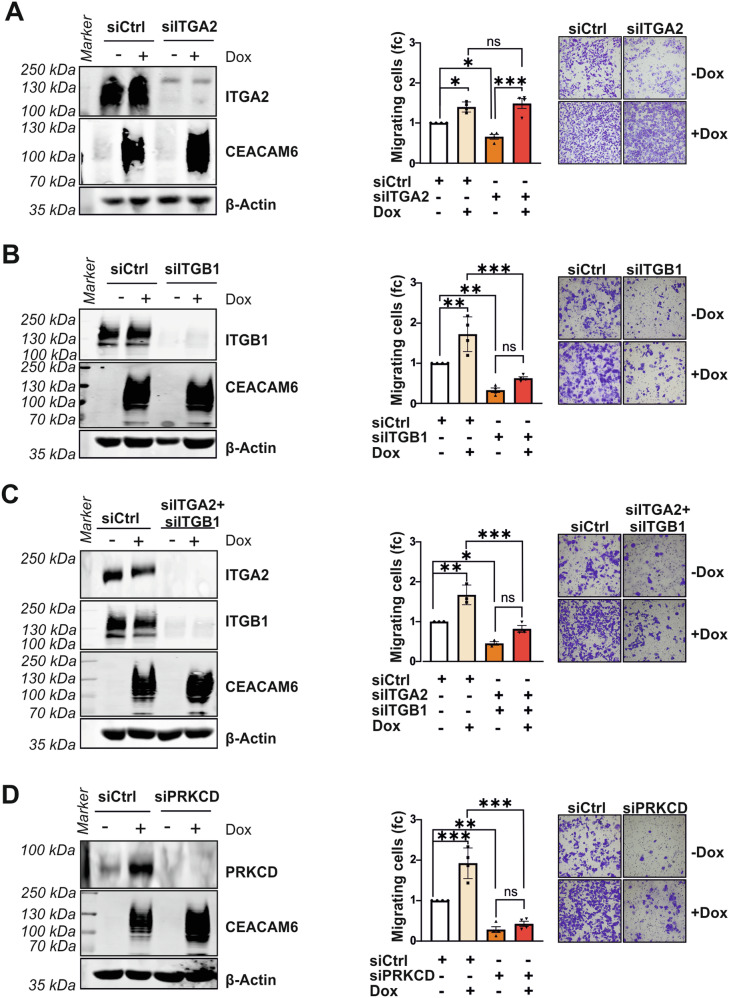
CEACAM6 collaborates with ITGB1 and PRKCD to regulate cell migration. **A** Western blots and transwell migration assay of GB-d1-CEACAM6 cells treated with Dox (2 µg/mL) to induce CEACAM6 overexpression and transfected with siPool (1 nM) to knockdown ITGA2, **B** ITGB1, **C** ITGA2 and ITGB1 together or **D** PRKCD. β-Actin served as loading control. Quantification of migrated cell area relative to the siRNA control (siCtrl) is presented in bar graphs and representative images are shown. Data are represented as mean ± SD of three to five independent experiments. *P* values were determined by ANOVA two-way followed by Sidak multiple comparisons test (*p* ≥ 0.05 ns, <0.05 *, <0.01 **, <0.001 ***).

Next, we explored the functional relevance of these protein-protein interactions in regard to cell migration. To answer this question, we performed transwell migration assays combining CEACAM6 overexpression with transfection of RNAi pools containing 30 different siRNAs targeting ITGA2, ITGB1, or PRKCD, respectively. As observed before, Dox-induced CEACAM6 expression increased cell migration, however, siITGA2 reduced cell migration but failed to rescue the effect of CEACAM6 in two different GBC cell lines (Figs. 7A and S8A). In contrast, inhibition of ITGB1 by siITGB1 prevented the CEACAM6-induced cell migration by reducing the cell migration to levels comparable to the control (Figs. 7B and S8B). The combined inhibition of ITGB1 and ITGA2 had effects comparable to ITGB1 alone suggesting that only ITGB1 inhibition is effective although ITGB1 and ITGA2 have been shown to directly interact as a heterodimer (Fig. 7C and S8C) [25]. CEACAM6 migratory effect also showed dependency on PRKCD expression with PRKCD knockdown abrogating the CEACAM6-induced migration phenotype (Figs. 7D and S8D). Thus, CEACAM6 directly interacted with ITGB1, ITGA2, and PRKCD and CEACAM6’s migratory phenotype depended on ITGB1 and PRKCD.

### AKT and ERK inhibitors interfere with CEACAM6-driven molecular functions in GBC cells

**Fig. 8:**
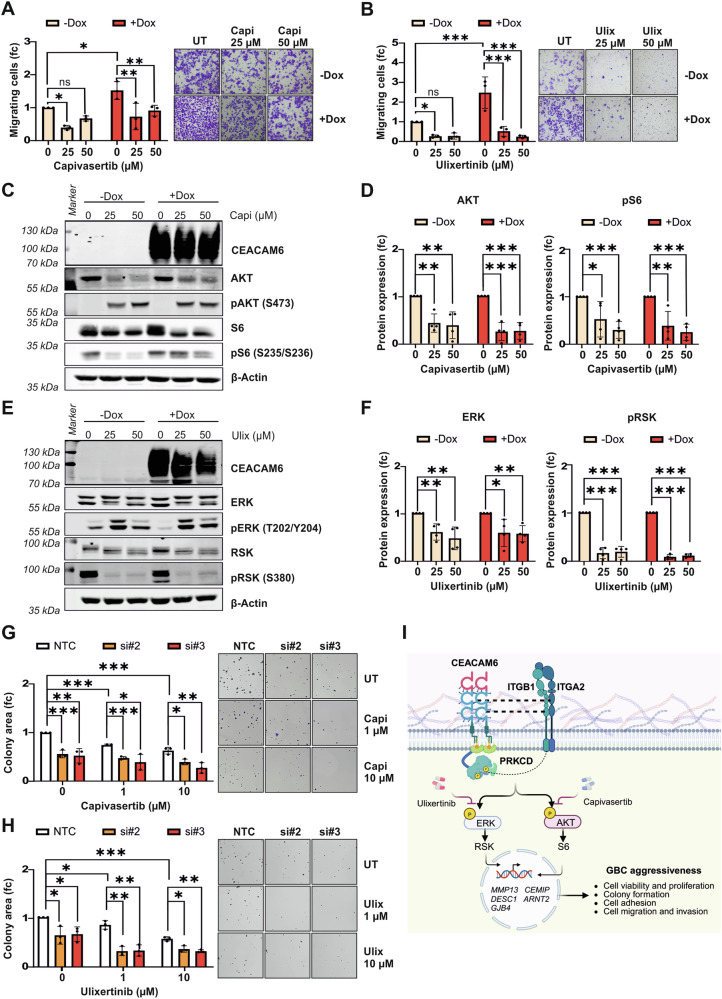
CEACAM6 function is inhibited by ERK and AKT inhibitors. **A** Cell migration transwell assay of GB-d1-CEACAM6 cells with or without CEACAM6 overexpression and capivasertib or **B** ulixertinib treatment for 24 h. Data are represented as mean ± SD of three independent experiments. *P* values were determined by ANOVA two-way followed by Sidak’s multiple comparisons test (*p* ≥ 0.05 ns, <0.05 *, <0.01 **, <0.001 ***). **C** Representative Western blot images of CEACAM6, AKT, pAKT (S473), S6, and pS6 (S235/S236) with or without CEACAM6 overexpression and capivasertib treatment for 24 h in GB-d1-CEACAM6 cells. **D** Western blot quantification of AKT and pS6 normalized to β-Actin which served as loading control. **E** Protein expression of CEACAM6, ERK, pERK (T202/Y204), RSK, and pRSK (S380) are shown in representative Western blot images of GB-d1-CEACAM6 cells with or without CEACAM6 overexpression and ulixertinib treatment for 24 h. **F** Quantification of ERK and pRSK normalized to β-Actin. Error bars depict SD and *p* values were determined by unpaired *t*-test (*p* ≥ 0.05 ns, <0.05 *, <0.01 **, <0.001 ***). **G** Quantification and representative images of colony forming area of CEACAM6 knockdown with or without capivasertib or **H** ulixertinib treatment in SNU308 cells. Data are represented as mean ± SD of three independent experiments. *P* values were determined by unpaired *t*-test (*p* ≥ 0.05 ns, <0.05 *, <0.01 **, <0.001 ***). **I** Schematic model of CEACAM6 molecular function and mechanism in the regulation of GBC aggressiveness. CEACAM6 overexpression in GBC leads to increased interaction between CEACAM6, the integrin ITGA2: ITGB1 receptor, and PRKCD. This protein-protein interaction regulates ERK and AKT downstream target genes related to cell migration such as *MMP13*, *CEMIP*, *DESC1*, *ARNT2,* and *GJB4*. Inhibition of ERK and AKT through molecular inhibitors or CEACAM6 knockdown decreased CEACAM6-induced GBC aggressiveness. This schematic model was illustrated with Biorender.com.

Intrigued by the finding that AKT and ERK signaling pathways were reduced by CEACAM6 knockdown (Fig. 3F–H), we asked whether AKT and ERK inhibition could effectively reduce CEACAM6-driven oncogenic function. To this end, we treated GB-d1 and TGBC1 cells with 25 and 50 µM of AKT inhibitor capivasertib or the ERK inhibitor ulixertinib. Capivasertib blocked CEACAM6-induced cell migration in both cell lines (Figs. 8A and S9A). Similar to the inhibition of AKT, ulixertinib effectively inhibited the CEACAM6-induced migration phenotype (Figs. 8B and S9B). Both capivasertib and ulixertinib did not significantly influence cell viability after 24 h of treatment (Fig. S9C, D). Consistent with previous studies, capivasertib reduced AKT protein expression and increased phosphorylation of AKT at S473 (pAKT) as a feedback mechanism after AKT inhibition (Figs. 8C, D and S9E, F) [26]. The AKT inhibition led to reduced phosphorylation of S6 at S235 and S236 (pS6, Figs. 8C, D and S9E, F). Ulixertinib also reduced ERK total protein and showed accumulation of pERK (T202/Y204) as a feedback mechanism compatible with the modes of action of ERK inhibitors (Figs. 8E, F and S9G, H) [27, 28]. As a result, phosphorylation of RSK at S380, downstream of the ERK signaling pathway, was abrogated (pRSK, Figs. 8E, F and S9G, H). To further dissect the effect of AKT and ERK inhibition on GBC cells, we analyzed low-dose treatment of capivasertib and ulixertinib in all four cell lines. The assessment of capivasertib and ulixertinib at concentrations of 0.1, 1, and 10 µM revealed that AKT and ERK protein inhibition was partially achieved at 1 µM, whereas inhibition at 10 µM was comparable to that observed at 25 and 50 µM for both treatments (Fig. S10A, B). Next, we evaluated the effect of 1 and 10 µM of capivasertib or ulixertinib on cell migration and cell invasion (Fig. S11). We observed that CEACAM6 significantly increased cell migration and invasion and 10 µM of capivasertib or ulixertinib was functionally effective to inhibit CEACAM6-driven migration and invasion in GB-d1-CEACAM6 and TGBC1-CEACAM6 cells (Fig. S11A–H). Therefore, the AKT inhibitor capivasertib and the ERK inhibitor ulixertinib effectively inhibited the CEACAM6-induced cell migration and invasion. To further support the hypothesis that inhibition of CEACAM6 and AKT or ERK signaling may be an effective strategy, we performed CEACAM6 knockdown with or without capivasertib or ulixertinib treatment. As observed before, siRNA-mediated inhibition of CEACAM6 reduced colony formation (Fig. 8G, H). In addition, the combination of CEACAM6 knockdown and capivasertib or ulixertinib treatment showed additive effects to further inhibit colony formation (Fig. 8G, H). Thus, the inhibition of AKT and ERK may serve as an effective strategy to counteract CEACAM6-driven oncogenic functions (Fig. 8I).

## Discussion

Similar to most other epithelial cancer entities, carcinomas of the biliary tree are very heterogeneous, and treatment options are limited. Despite recent advances in the understanding of molecular alterations in BTC, most research focused on CCA and the relatively low numbers of patients with GBC included in these studies limits the findings regarding GBC [3]. Due to late diagnosis, a disproportionally high recurrence rate after resection, and the lack of targeted therapies, patient overall survival is still very dismal [2, 3]. Adjuvant therapies using gemcitabine in combination with platinum-based compounds such as oxaliplatin and cisplatin are the first-line therapy for unresectable GBC, but overall survival is only minimally extended [29]. Genetic analyses revealed that GBC rarely harbor microsatellite instability and the most observed mutations, such as *TP53*, *ARID1*, *ARID2*, *ELF3,* and *KRAS*, are not targetable [30, 31]. In addition, GBC are considered to be immune cold tumors and unfortunately, only few patients with GBC show response to immunotherapy [32, 33]. *ERBB2*/*ERBB3* amplifications and mutations have been identified in a subset of patients with GBC and first clinical trials showed promising results [30, 34–36]. Interestingly, *ERBB2*/*ERBB3* mutations promote PD-L1-mediated immune escape suggesting that combination therapies of pan-HER inhibition and immunotherapy may be effective in patients with *ERBB2*/*ERBB3* alteration [37, 38].

Although genetic profiling is crucial, proteins reflect the pathophysiology of a disease and are ideal predictors of disease progression and the major active therapeutic targets [39]. Thus, we performed shotgun mass-spectrometric analysis and identified a large number of proteins differentially expressed in GBC which were involved in pathways promoting progression. Among the most significantly upregulated proteins, we selected CEACAM6 as it is localized in the cell membrane and little was known about its function in GBC. We demonstrated that (1) CEACAM6 promoted GBC aggressiveness in vitro and in in vivo mouse models, (2) CEACAM6 directly interacted with ITGB1 and PRKCD mediating CEACAM6-induced GBC cell migration and (3) inhibition of AKT and ERK as key nodes in CEACAM6 downstream signaling pathways may be effective therapeutic approaches in GBC. We also shed light on the complex landscape of the CEACAM protein family in the context of GBC. Contradicting reports about the molecular function of the CEACAM protein family exist. For instance, CEACAM1 has been reported to be downregulated in many patients of prostate, colon, and breast cancer suggesting its tumor-suppressing properties [40]. In contrast, CEACAM5 expression is high in patients with lung adenocarcinoma [41], gastric cancer [42], and colorectal cancer [11, 43] and correlated with poor clinical outcomes and overall survival [44, 45]. Here, we detected CEACAM6, CEACAM5, CEACAM7, and CEACAM8 by mass-spectrometry which are structurally similar (Fig. S12A). We also performed immunohistochemical analysis of CEACAM5 and found that CEACAM5 was upregulated in the GBC epithelium compared to the normal gallbladder epithelium, however, the CEACAM5 expression was not associated with patient outcome (Fig. S12B–E). The lack of association of CEACAM5 and CEACAM6 with patient survival may be explained by the overall poor outcome and aggressiveness of GBC. Furthermore, CEACAM6 was functionally involved in the initial steps of cancer progression and metastasis and may not be required for late-stage metastasis. This is further supported by the notion that all cases with early-stage GBC were positive for CEACAM6, whereas in later-stage GBC more than one-third of patients were negative for CEACAM6 suggesting that CEACAM6 is early in carcinogenesis upregulated but not required or even had negative effects in advanced stage GBC. CEACAM5 knockdown similarly to CEACAM6 resulted in reduced migration of GBC cells suggesting similar roles for CEACAM5 and CEACAM6 (Fig. S12F–I). This finding also suggested that CEACAM5 and CEACAM6 do not compensate each other which is possibly due to their differential expression patterns, protein structure, and downstream effects [46]. It is important to note that CEACAM5 protein was highly expressed in immune cells of the gallbladder (NT tissue, Fig. S12C) explaining why CEACAM5 was not differentially expressed in our mass-spectrometry analysis. In addition, the expression of CEACAM5 in immune cells prevents the therapeutic use of CEACAM5 inhibitors. Therefore, CEACAM6 stands out as potential therapeutic target.

CEACAM6 reduced GBC cell adhesion while promoting cell migration and invasion without affecting cell proliferation. This finding mirrors the nature of advanced GBC which invades the gallbladder wall and metastasizes to regional lymph nodes, liver, pancreas, or duodenum [1, 3]. The knockdown of CEACAM6 did not only inhibit cancer cell migration by increasing cell adhesiveness but also reduced overall cell vitality, increased senescence, and led to a block in cell cycle progression. CEACAM6 knockdown did not induce apoptosis, PARP cleavage, or p16 consistent with our findings of reduced cells proportions in S phase [23]. We also translated our findings to the in vivo situation in a xenograft mouse model, in which we validated CEACAM6’s role in the metastasis process. We found that GBC cells with CEACAM6 overexpression had a lower ability to adhere to the lung when injected via the tail vein of mice, showing reduced adhesion, which is essential for cell migration. Most of the CEACAM proteins, including CEACAM6, are expressed only in humans but absent in mice [47]. Thus, the xenograft model provides an attractive in vivo model to study CEACAM6 in human GBC. Future studies using mouse gallbladder organoids to functionally validate CEACAM6 may be useful to further study therapeutic approaches [48].

Furthermore, untargeted mass-spectrometric BirA-BioID revealed CEACAM6 interaction partners. Among the CEACAM6 binding proteins, we further studied the two integrin subunits, ITGB1 and ITGA2, and PRKCD. Interestingly, CEACAM6 has been previously shown to co-localize with integrin in myoblasts [49]. Overexpression of ITGB1 and ITGA2 is linked to carcinogenesis in epithelial cells increasing migration and invasion and contributing to EMT [50]. ITGA2 exclusively forms a dimer with ITGB1 as an integrin α2β1 heterodimer, but ITGB1 can form a heterodimer with at least 12 different α subunits [25]. This could explain our finding that the CEACAM6 migratory function is partially dependent on CEACAM6-ITGB1 interaction, but not on CEACAM6-ITGA2 interaction. We also showed that the absence of PRKCD abrogated CEACAM6-induced migration. PRKCD is a member of the Protein Kinase C protein family which requires allosteric lipid activation [51]. PRKCD links integrin to its downstream effectors, such as phospholipase D, to activate actin cytoskeletal rearrangement and support integrin-mediated cell migration [52]. We consistently demonstrated the interaction between PRKCD and ITGB1 in GBC. Given that CEACAM6 has no intracellular domain, it is tempting to speculate that CEACAM6, ITGB1, and PRKCD interact through the formation of a complex within lipid rafts [53]. The ITGB1/PRKCD/CEACAM6 complex appears to be indispensable for the oncogenic effects of CEACAM6, particularly in cell migration.

We also identified that CEACAM6 oncogenic properties in supporting cell migration may act through regulation of ERK and AKT signaling and their downstream gene effectors. Among the validated genes were *MMP13*, *CEMIP*, *DESC1*, *ARNT2, and GJB4*. *MMP13*, *CEMIP,* and *DESC1* encode proteins important for degradation of extracellular matrixes, expressed in the tumor invasion front, and are reported to increase cell migration and tumor cell extravasation [54–56]. ARNT2 is a transcription factor contributing to the regulation of angiogenesis, hypoxic responses, and xenobiotics metabolism through its interaction with HIF-1α, AHR, and other basic helix-loop-helix transcription factors [57, 58]. Furthermore, GJB4 has been shown to be correlated with AKT pathway activation inducing cell proliferation and metastasis [59]. Thus, CEACAM6 activated ERK and AKT signaling leading to the expression of metastasis-promoting genes.

When treated with AKT and ERK inhibitors, GBC cell migration was abrogated in cells with high CEACAM6 expression levels. This finding opens the door to further evaluate ERK and AKT inhibitors as potential treatment options for GBC. Interestingly, previous studies utilized CEACAM6-targeted antibodies to deliver anticancer drugs such as maytansinoid (DM1) [60] and gemcitabine [61] for PDAC therapy or to deliver doxorubicin in NSCLC [62] and paclitaxel [61] in lung cancer. Further investigation to coupled AKT and ERK inhibitors with anti-CEACAM6 antibodies may offer an intriguing approach for GBC targeted therapy. Therefore, our approach to select the overexpressed cell membrane protein CEACAM6 for further analysis may guide the development of antibody-drug conjugate therapy for GBC to overcome the high toxicity of the first-line chemotherapies currently used.

Taken together, our study provided untargeted quantitative mass-spectrometry-based proteomics of GBC and led to the identification of the protumorigenic cell membrane protein CEACAM6. We demonstrated that CEACAM6-inhibition greatly reduced GBC aggressiveness. Furthermore, AKT and ERK inhibitors effectively inhibited the CEACAM6-induced phenotype suggesting that combination therapies or CEACAM6-antibody drug conjugate therapies may offer a promising strategy in GBC therapy. Taken together, these findings provide a foundation for further studies of CEACAM6 and potential avenues for therapeutic developments in the fight against GBC aggressiveness.

## Supplementary information

Supplementary Data

Western blot images

Table S2

Table S3

Table S4

Table S5

Table S6

Table S7

Table S8

Table S9

Table S10

## Data Availability

The datasets generated in this study are available in the GEO repository with accession number GSE243306.
