# Supplementary material for: Proteomic profiling reveals CEACAM6 function in driving gallbladder cancer aggressiveness through integrin receptor, PRKCD and AKT/ERK signaling
Source: Cell Death Dis. 2024 Oct 28;15(10):780. doi: 10.1038/s41419-024-07171-x (PMC11519453; doi:10.1038/s41419-024-07171-x)
Supplement: Supplementary file 1 — Supplementary Data [file 41419_2024_7171_MOESM1_ESM.pdf]

Supplementary Data

Proteomic profiling reveals CEACAM6 function in driving gallbladder cancer aggressiveness through integrin receptor, PRKCD and AKT/ERK signaling

Sugiyanto *et al.*

Corresponding author: Stephanie Roessler, Email: Stephanie.Roessler@med.uni-heidelberg.de

Table of Content

Supplementary Methods **2**

Supplementary Figures **10**

Figure S1. Expression profiling of CEACAM6 and CEACAM protein family members. 10

Figure S2. CEACAM6 knockdown inhibits GBC oncogenic functions. 12

Figure S3. CEACAM6 regulates cell adhesion and initial step of cancer metastasis. 14

Figure S4. Validation of CEACAM6-C-BirA-Flag protein expression for CEACAM6 interacting partners elucidation through BioID. 16

Figure S5. Subcellular localization of endogenous and overexpressed CEACAM6 based on immunofluorescence. 17

Figure S6. ITGA2, ITGB1 and PRKCD colocalize with CEACAM6 in subcellular locations. 18

Figure S7. CEACAM6 interacts with ITGA2, ITGB1 and PRKCD in SNU308 and Mz-ChA-1 cells. 19

Figure S8. CEACAM6 collaborates with ITGB1 and PRKCD to regulate cell migration. 20

Figure S9. CEACAM6 function is inhibited by ERK and AKT inhibitors. 21

Figure S10. Dose titration of capivasertib and ulixertinib for AKT and ERK inhibition. 23

Figure S11. Capivasertib and ulixertinib treatment inhibit CEACAM6-induced migration and invasion. 24

Figure S12. CEACAM5 is required for GBC cell migration. 25

Supplementary Tables **27**

Table S1. Patient characteristics. 27

References **28**

Supplementary Tables S2-S10 are provided online in separate Excel files.

Supplementary Methods

Cell lines

Eight GBC cell lines (Mz-ChA-1, OZ, NOZ, TGBC1, TGBC2, YoMi, SNU308 and GB-d1), HeLa and HEK293T cells were used in this study. Routine checks for mycoplasma contamination (MycoAlert, Lonza, Basel, Switzerland) and authenticated by STR analysis were performed. OZ and NOZ were cultivated in William's E medium, SNU308 was cultured in RPMI1640, Mz-ChA-1, GB-d1, TGBC1, TGBC2, YoMi, HeLa and HEK294T were grown in DMEM medium. All media were supplemented with 10% fetal bovine serum (FBS; Thermo Fisher Scientific, Waltham, USA) and 1% Penicillin-Streptomycin (100 IU/mL and 100 g/mL). All media and Penicilin-Streptomycin were obtained from Sigma-Aldrich, Taufkirchen, Germany. Cell lines were cultured in a 37 °C incubator with a 5% CO_2 atmosphere.

Plasmid cloning

CEACAM6 and PRKCD cDNAs were obtained from Genewiz (Azenta Life Science, Leipzig, Germany) and then cloned into pDONR201. ALB, ITGA2 and ITGB1 cDNAs were provided by Stefan Pusch in the pDONR201, pDONR223 and pDONR221, respectively. Gateway cloning using the LR-Clonase was performed to transfer the genes into destination vectors pDEST-GW, pDEST-N-HA-GW or pDEST-N-Flag-GW for transient transfection, into pTRIPZ-GW-Puro and pTRIPZ-GW-C-BirA-Flag-Puro for Doxycycline (Dox)-induced gene expression or into pFUGW-Pol2-ffLuc2-eGFP and pLenti-CMV-GW-Puro for constitutive gene expression.

Generation of stable constitutive and Dox-inducible cell lines

The plasmid construct (10 µg) was transfected together with packaging vectors pMD2.G (2.5 µg; Addgene #12259, http://n2t.net/addgene:12259; RRID:Addgene_12259) and psPAX2 (8 µg; Addgene #12260, http://n2t.net/addgene:12260; RRID:Addgene_12260) in 1 mL Optimized Minimal Essential Medium (OptiMEM, Thermo Fisher Scientific, Waltham, MA, USA) with 60 µL Polyethylenimine (PEI; Polysciences, Warrington, PA, USA) into HEK293T. The medium was changed after 16 hours. After additional 24 hours of incubation, the supernatant containing lentiviral particles was filtered using a 0.45 µm Millex-HA filter (Merck Millipore, Burlington, MA, USA) and utilized to infect the target cell lines. For stable inducible cell lines, Puromycin (Puro, 2 µg/mL, Sigma-Aldrich, Taufkirchen, Germany) was used to select positively infected cells and were treated with or without Doxycycline (Dox; 2 µg/mL or 1 µg/mL; Sigma-Aldrich, Taufkirchen, Germany) to induce the expression of the respective proteins. For cells transduced with pFUGW-Pol2-ffLuc2-eGFP (Addgene #71394)[1], FACS was performed to select positive cells expressing low levels of GFP.

Oligonucleotides and plasmids transient transfection

For co-immunoprecipitation (co-IP) experiments, 2.5 million HEK293T cells were seeded in 15 cm dishes coated with Poly-L-Lysine (PLL; Sigma-Aldrich, Taufkirchen, Germany). The next day cells were transfected with 30 µg of plasmids using 90 µL PEI (Polysciences, Warrington, PA, USA) in 1.5 mL OptiMEM (Thermo Fisher Scientific, Waltham, MA, USA). The medium was changed the next day and after additional 24 hours cells were harvested for co-IP experiments.

CEACAM6 and CEACAM5 targeting siRNAs were obtained from Qiagen and are listed in Supplementary Table S10 (Qiagen, Germantown, MD, US). ITGA2, ITGB1, PRKCD targeting siPools or negative control siPools were obtained from siTools Biotech and were used at 1 nM concentration (siTOOLs Biotech, Martinsried, Germany). All siRNA-mediated knockdown

experiments were performed using Lipofectamine RNAiMAX Transfection Reagent (Thermo Fisher Scientific, Waltham, MA, USA) according to the manufacturer's instructions.

Protein extraction and Western blot

Proteins were extracted with cell lysis buffer (Cell Signaling Technology, Frankfurt am Main, Germany) supplemented with 1x PhosStop and 1x protease inhibitor Complete Mini EDTA-free (Roche Diagnostics, Mannheim, Germany). Cells were lysed using sonication for 3 x 30 seconds with 1-minute incubation on ice in between. Then, cells were centrifuged at 14,000 rpm at 4 °C for 15 minutes. The protein concentration was determined using Bradford's assay according to the manufacturer's instructions (Sigma-Aldrich, Taufkirchen, Germany). The absorbance was measured at 595 nm using the Omega FLUOStar microplate reader (BMG LABTECH, Ortenberg, Germany) and calculated based on BSA standard curve. The samples were prepared by adjusting the protein lysates with 4x Laemmli buffer and nuclease-free water to solutions with a final concentration of 1 µg/µL and denatured at 95 °C for 8 minutes. Protein samples (25-50 µL) and 5 µL of PageRuler™ or PageRuler™ Plus pre-stained protein ladder (Thermo Fisher Scientific, Waltham, USA) as reference were loaded onto an 8-10% Bis/Tris polyacrylamide gel and separated by electrophoresis at 80 V and 150 V for 1.5 hours. Proteins were transferred onto a nitrocellulose membrane by wet blotting for 2.5 hours at 90 V or overnight at 35 V and blocked with 5% milk in TBST at room temperature for 1 hour. Proteins were immunoblotted with indicated antibodies overnight at 4 °C or for 2 hours at room temperature and detected with IRDye secondary antibodies using an Odyssey Sa Infrared Imaging System (LI-COR Biosciences, Bad Homburg, Germany). Protein abundance was quantified using Image Studio v3.1.4 (LI-COR Biosciences).

RNA extraction, cDNA synthesis and semi-quantitative reverse-transcription polymerase chain reaction (qRT-PCR)

Total RNA was extracted using the ExtractMe Total RNA Kit (Blirt, Gdansk, Poland) according to the manufacturer's instructions and concentration was confirmed using the Nanodrop ND-1000 spectrophotometer (Thermo Fisher Scientific, Waltham, USA). cDNA was synthesized from 0.5 to 1 µg total RNA using the Takara PrimeScript RT Reagent protocol (Takara Bio Europe SAS, Saint-Germain-en-Laye, France). Gene expression levels of specific genes were analyzed by qRT-PCR with the use of the PrimaQUANT qPCR-CYBR-Green-MasterMix-high-ROX (Steinbrenner, Heidelberg, Germany) on QuantStudio™ 5 Real-Time PCR (Applied Biosystems, Darmstadt, Germany). The internal reference gene for relative quantification was the serine/arginine-rich splicing factor 4 (SRSF4). Relative mRNA expression levels were calculated using the ΔΔCt method. Primers were obtained from Thermo Fisher Scientific and are listed in Supplementary Table S10.

Cell viability assay

For CEACAM6 overexpression experiments, cells were seeded in 12- or 24-well plates in triplicates and treated with Dox (2 µg/mL) the next day. For CEACAM6 knockdown, cells were seeded in 12-well plates in triplicates after respective siRNA knockdown. To investigate the effect of AKT inhibitor (capivasertib, MedChemExpress Monmouth Junction, NJ, USA) and ERK inhibitor (ulixertinib, MedChemExpress Monmouth Junction, NJ, USA), GB-d1-CEACAM6 or TGBC1-CEACAM6 cells were seeded in 24-well plates and were treated without or with Dox (2 µg/mL) for 24 hours, followed by the treatment using 0 µM, 25 µM or 50 µM of the respective inhibitor. Cells were incubated in medium containing 10% Resazurin (R&D Systems, Minneapolis, MN, USA) for 1 hour at 37 °C. Absorbance was measured at 544 nm

Ex/590 nm Em using the Omega FLUOstar Microplate Reader (BMG LABTECH, Ortenberg, Germany) every 24 hours.

Cell proliferation BrdU-ELISA assay

To measure cell proliferation of cells with CEACAM6 knockdown, Mz-ChA-1 and SNU308 cells were seeded in 6 cm dishes. Cells were reverse-transfected with NTC or siCEACAM6 using Lipofectamine™ RNAiMAX transfection reagents (Life Technologies, Darmstadt, Germany). Six hours post transfection, the medium was changed and cells were incubated for 48 hours before re-seeded into 96-well plates with 10,000 cells/well. After 2 days of incubation, the medium was replaced by growth medium containing 1:10 BrdU (100 µL/well) for 1 hour and the instruction of Cell Proliferation Biotrak™ Version 2 ELISA assay (Sigma-Aldrich, Taufkirchen, Germany) were followed. Finally, the plate was measured in the Omega FLUOstar Microplate Reader at 450 nm (BMG LABTECH, Ortenberg, Germany).

Colony formation assay

For colony formation, SNU308 and Mz-ChA-1 cells were seeded into 6-well plates with 1000 cells per well. The next day, cells were transfected with NTC or siCEACAM6 using Lipofectamine™ RNAiMAX transfection reagents (Life Technologies, Darmstadt, Germany) in 3 mL medium. The cells were incubated for 14 days.

For combination with CEACAM6 knockdown, SNU308 cells were seeded in 6-well plates with 250,000 cells/well. The next day, cells were transfected with NTC or siCEACAM6 using Lipofectamine™ RNAiMAX transfection reagents (Life Technologies, Darmstadt, Germany). Six hours post transfection, the medium was changed. The next day, AKT inhibitor (capivasertib, MedChemExpress Monmouth Junction, NJ, USA) and ERK inhibitor (ulixertinib, MedChemExpress Monmouth Junction, NJ, USA) with concentration of 0, 1, or 10 µM were added. After 24 hours cells were re-seeded for colony formation with 1000 cells per well in 3 mL medium.

At the end of the incubation period, cells were washed with PBS and stained with 0.5% crystal violet in 25% methanol for 45 minutes at room temperature. Subsequently, the crystal violet staining solution was removed and the 6-well plates were thoroughly washed with double-distilled water until the staining solution was completely removed. Pictures of the plates were taken and the number of colonies or the area covered by colonies was counted using FIJI software.

Cell adhesion assay

Cells were seeded on 12-well plates with 100,000 cells per well, following Dox induction or siRNA-mediated knockdown. After 1 hour of incubation at 37 °C, cells were washed and the remaining attached cells were stained with 0.5% crystal violet in 25% methanol for 45 minutes at room temperature. Subsequently, the crystal violet staining solution was removed and the plates were thoroughly washed with double-distilled water. Adherent cells were observed under a microscope and cell numbers were calculated using FIJI software.

Transwell migration and invasion assay

To perform migration and invasion assay of ALB or CEACAM6 overexpressing cells, GB-d1 and TGBC1 were seeded in 10 cm dishes. Dox (2 µg/mL) was added to the medium the following day. After 24 hours, cells were harvested and 75,000 cells were seeded in medium without FCS in the upper chamber of migration transwell inserts Falcon® Permeable Support 8.0 µm or in previously equilibrated BioCoat® Matrigel® Invasion Chambers 8.0 µm (both

Corning Incorporated, NY, USA) for invasion assay. Transwell inserts were placed in a Falcon® 24-well Companion Plate (both Corning Incorporated, NY, USA) filled with 750 µL medium with FCS as chemoattractant.

For CEACAM6 or CEACAM5 knockdown experiments, SNU308 and Mz-ChA-1 cells were seeded on 6 cm dishes with 400,000 cells per dish. NTC or siRNAs targeting CEACAM6 or CEACAM5 were reverse-transfected using Lipofectamine™ RNAiMAX transfection reagents (Life Technologies, Darmstadt, Germany). 6 hours post-transfection, 1 mL of fresh medium was added and the medium was changed into 3 mL fresh medium the following day. After 3 days of incubation, cells were harvested and re-seeded on the upper chamber of transwell inserts with 200,000 or 150,000 cells for SNU308 and Mz-ChA-1, respectively.

GB-d1 and TGBC1 cells with inducible CEACAM6 were also used for migration experiments with knockdown of ITGA2, ITGB1, a combination of ITGA2 and ITGB1 or PRKCD. The siPools (1 nM in total) targeting NTC, ITGA2, ITGB1, a combination of ITGA2 and ITGB1 or PRKCD were reverse-transfected using Lipofectamine™ RNAiMAX transfection reagents (Life Technologies, Darmstadt, Germany) into GB-d1-CEACAM6 or TGBC1-CEACAM6 cells. For each well, 250,000 cells/well were seeded in 6-well plates. The medium was changed with 2 mL fresh medium without or with Dox (2 µg/mL) the following day to induce the expression of CEACAM6. After 24 hours, cells were harvested and 75,000 cells were re-seeded in the upper chamber of migration transwell inserts in 750 µL DMEM medium without FCS. DMEM medium with FCS was added to the bottom chamber as a chemoattractant.

To investigate the effect of AKT inhibitor (capivasertib, MedChemExpress Monmouth Junction, NJ, USA) and ERK inhibitor (ulixertinib, MedChemExpress Monmouth Junction, NJ, USA), GB-d1-CEACAM6 or TGBC1-CEACAM6 cells were seeded in 6-well plates with 250,000 cells/well. Cells were treated without or with Dox (2 µg/mL) for 24 hours, followed by the treatment using 0, 1, 10, 25 or 50 µM of the respective inhibitor. After 24 hours cells were re-seeded for transwell assays as described above.

After 18-20 hours of incubation, the upper part of the transwell insert was swabbed gently using a cotton swab. Cells were fixed in methanol and stained with 0.5% crystal violet in 25% methanol for 1 hour. The transwell inserts were thoroughly washed with double-distilled water until the staining solution was completely removed. Pictures of the inserts were taken and the area of migrating or invading cells was counted using FIJI software.

Cell cycle assay

For cell cycle analysis, SNU308 and Mz-ChA-1 cells were seeded in 6-well plates with 250,000 cells per well in triplicates. Cells were transfected with NTC or siRNAs targeting CEACAM6 using Lipofectamine™ RNAiMAX transfection reagents (Life Technologies, Darmstadt, Germany). After 6 hours, 0.5 mL fresh medium was added and the next day, the medium was changed to fresh medium. Three days after transfection, cells were harvested, washed with PBS and fixed with 200 µL ice-cold 70% ethanol while vortexing at low speed. After 1 hour of incubation at 4 °C, cells were washed with PBS, incubated with a staining solution containing 50 µg/mL propidium iodide (Sigma-Aldrich, Taufkirchen, Germany) and 200 µg/mL RNAse (Thermo Fisher Scientific, Waltham, MA, USA) in PBS for 30 minutes at 37 °C. DNA amount was determined by flow cytometry using the Guava easyCite HT system (Merck Millipore, Darmstadt, Germany).

Apoptosis assay

SNU308 and Mz-ChA-1 cells were seeded on in 6 cm dishes with 400.000 cells per dishes. Cells were reverse-transfected with NTC or siCEACAM6 using Lipofectamine™ RNAiMAX transfection reagents (Life Technologies, Darmstadt, Germany). Six hours post transfection, the medium was changed and cells were incubated for 48 hours before re-seeded into 96-well plates with 10,000 cells/well. After two days incubation, apoptotic cells were detected according RealTime-Glo™ Annexin V Apoptosis and Necrosis Assay protocol (Promega,Madison, WI, USA).

Senescence assay

For β-galactosidase staining, SNU308 and Mz-ChA-1 cells were seeded on PLL coated 18 mm glass coverslips with 50,000 cells per coverslip. The next day, cells were transfected with NTC or siRNAs targeting CEACAM6 using Lipofectamine™ RNAiMAX transfection reagents (Life Technologies, Darmstadt, Germany). After 6 hours, 0.5 mL fresh medium was added and the medium was changed to fresh medium the next day. Three days after transfection, cells were washed twice with PBS and fixed with 0.5% glutaraldehyde in PBS (Thermo Fisher Scientific, Waltham, MA, USA) for 15 minutes at room temperature. Then cells were washed twice with 1 mM pH 6.0 $MgCl_2$ in PBS and stained for 4 hours at 37 °C with freshly prepared X-Gal staining solution containing 40 mg/mL X-Gal in N,N-dimethylformamide (Roche, Mannheim, Germany), KC Buffer (K3Fe(CN)6 and K4Fe(CN)6*3H2O in PBS) and 1 mM pH 6.0 MgCl2 in PBS. After staining, coverslips were stored with 70% glycerol and pictures were taken using a Nikon Ni-E microscope with Nikon Plan Apo λ 10x NA 0.45 objective and Nikon DS-Ri2 color camera. The positively stained area was calculated using FIJI Software.

Gene expression and pathway analysis

For RNA sequencing analysis, total RNA of GB-d1-CEACAM6 cells treated with 2 µg/mL Dox for 24 h or left untreated as control was isolated using the Macherey-Nagel RNA Extraction Kit (Macherey-Nagel, Düren, Germany). In addition, total RNA of SNU308 cells tranfected with NTC (non-targeting control), siCEACAM6#2 or siCEACAM6#3 was extracted 3 days after transfection. Subsequently, 2 µg of total RNA was sent to BGI Hongkong Tech Solution for mRNA sequencing using the DNBseq™ platform. For library preparation, oligo dT beads were used to enrich mRNA and cDNA was synthesized with respective adaptor sequences. Sequencing was performed with phi29 and single end 50 base reads were generated in the way of combinatorial Probe-Anchor Synthesis.

Analysis of RNAseq data was done with R and bioconductor using the next generation sequencing (NGS) analysis package systempipeR[2]. Quality control of raw sequencing reads was performed using FastQC (https://www.bioinformatics.babraham.ac.uk/projects/fastqc/). Low-quality reads were removed using trim_galore (version 0.6.4). The resulting reads were aligned to human genome version GRCh38.p13 from GeneCode and counted using kallisto version 0.46.1[3]. The count data was transformed to $\log_2$-counts per million (logCPM), estimated the mean-variance relationship and used this to compute appropriate observational-level weights for linear modelling using the voom-function from the limma package[4]. Differential expression analysis was performed using the limma package in R. A false positive rate of $\alpha = 0.05$ with FDR correction was taken as the level of significance. The raw and normalized data were deposited in the GEO database (https://www.ncbi.nlm.nih.gov/geo/; accession number GSE243306).

RNA sequencing data (Supplementary Table S5,S7) were analyzed by Gene Set Enrichment Analysis (GSEA) and Qiagen Ingenuity Pathway Analysis (IPA) software for enriched pathways (Supplementary Table S6,S8).

Co-immunoprecipitation (co-IP)

Cells were lysed in 1 mL of FLAG-CoIP lysis buffer (50 mM Tris HCl pH 7.5, 150 mM NaCl, 1 mM MgCl, 1% NP-40, 1 mg/mL BSA) supplemented with 1x PMSF and 1x protease inhibitor Complete Mini EDTA-free (Roche Diagnostics, Mannheim, Germany). For each reaction, 50 µg of total protein lysates were used as input and 5 mg of total protein lysate was immunoprecipitated using 40 µL Anti-FLAG® M2 Magnetic Beads (Sigma-Aldrich GmbH, Taufkirchen, Germany) overnight at 4 °C while rotating. After washing with TBST, the immunoprecipitated proteins were eluted with 40 µL 4x Laemmli buffer by boiling at 95 °C for 3 minutes and analyzed by Western blot.

Immunofluorescence

GB-d1-CEACAM6 or GB-d1-CEACAM6-C-BirA-Flag cells were seeded on 18 mm cover glasses coated with PLL (Sigma-Aldrich, Taufkirchen, Germany). Cells were treated with or without Dox (2 µg/mL) for 24 hours. To observe endogenous expression of CEACAM6, SNU308 or Mz-ChA-1 cells were seeded on 18 mm cover glasses coated with PLL (Sigma-Aldrich, Taufkirchen, Germany) and incubated for 48 hours. To analyze the effect of CEACAM6 knockdown on γ-H2AX expression, SNU308 and Mz-ChA-1 cells were seeded on 12 mm cover glasses coated with PLL (Sigma-Aldrich, Taufkirchen, Germany). The next day, cells were transfected with NTC or siCEACAM6 using Lipofectamine™ RNAiMAX transfection reagents (Life Technologies, Darmstadt, Germany). Cells were incubated for 48 hours. For Staurosporine control group, SNU308 cells were treated with 25 µM and Mz-ChA-1 cells with 5 µM Staurosporine for 2 hours before cell fixation (Santa Cruz Biotechnology, Dallas, TX, USA).

Cells were washed with PBST and fixed with paraformaldehyde (PFA) for 15 minutes. After washing with PBST, cells were permeabilized with 0.02% Triton-X100 in PBS for 10 minutes. Cells were blocked with 0.05% BSA in PBST for 30 minutes at room temperature. Primary antibodies listed in Supplementary Table S10 were added and incubated at 4 °C overnight. Cells were washed with PBST and incubated with secondary antibodies for 1 hour at room temperature followed by washing. Coverslips were mounted onto glass slides using DAPI Fluoromount-G® Mounting Medium (Thermo Fisher Scientific, Waltham, MA, USA). Cells were examined with Nikon C2 Plus confocal microscope and Nikon Apo λS 60x NA 1.40 oil immersion objective. Image processing was conducted by FIJI software. To evaluate the percentage of colocalization for each comparison five different images with 60x magnification including at least 10 cells per image were used for calculation of Mander's coefficient using the JaCoP plugin in FIJI software.

Proximity ligation assay (PLA)

GB-d1-CEACAM6-C-BirA-Flag cells were seeded on 18 mm cover glasses coated with PLL. Cells were treated with or without Dox (2 µg/mL) to induce CEACAM6 expression for 24 hours. Cells were fixed with 4% PFA and permeabilized with 0.2% Triton X-100/PBS. PLA was performed according to the Naveni™ Proximity Ligation (Navinci, Upssala, Sweden) or Duolink® (Sigma-Aldrich, Taufkirchen, Germany) in situ assay protocol. Briefly, slides were first blocked with blocking solution and incubated with two primary antibodies binding the proteins of interest overnight at 4 °C. After washing with TBST, coverslips were incubated in antibodies conjugated to proprietary oligonucleotide arms for 1 hour at 37 °C. To detect interactions,

enzymatic reactions were performed subsequently at 37 °C followed by washing steps in between reactions according to the instructions. Coverslips were mounted onto glass slides using the DAPI Fluoromount-G® Mounting Medium (Thermo Fisher Scientific, Waltham, MA, USA) and cells were imaged with the Nikon C2 Plus confocal microscope and Nikon Apo λS 60x NA 1.40 oil immersion objective. Image processing was conducted by FIJI software.

BirA BioID

GB-d1-Ctrl-BirA-Flag and GB-d1-CEACAM6-C-BirA-Flag were seeded in 15 cm dishes with 2.5 million cells each. Cells were treated with 2 µg/mL Dox (Sigma-Aldrich, Taufkirchen, Germany) for 24 hours to induce the expression of Ctrl-BirA-Flag or CEACAM6-C-BirA-Flag fusion protein. Biotin (50 µM, Sigma-Aldrich GmbH, Taufkirchen, Germany) was added the following day to start the biotinylation reaction of interacting and surrounding proteins of the fusion proteins. After 24 hours, cells were harvested with 1000 µL BioID lysis buffer (500 mM Tris pH 7.5, 200 mM NaCl, 0.1% SDS, 1% Triton X-100, 1mM EDTA and 0.25% Na-desoxycholat). Cells were lysed using sonication for 3 x 30 seconds with 1-minute incubation on ice in between. Then, cell lysates were centrifuged at 14,000 rpm at 4 °C for 15 minutes. To pull down the biotinylated proteins, 100 µL of Dynabeads™ MyOne™ Streptavidin C1 (Thermo Fisher Scientific, Waltham, MA, USA) were washed twice with 100 µL BioID lysis buffer, 1000 µL cell lysate was added and incubated at 4 °C with rotation overnight. The beads were washed subsequently with 500 µL BioID washing buffer 1 (2% SDS), 500 µL BioID washing buffer 2 (500 mM Tris pH 7.5, 500 mM NaCl, 1% Triton X-100, 1 mM EDTA and 6.1% Na-Desoxycholat), 500 µL BioID washing buffer 3 (10 mM Tris pH 7.5, 250 LiCl, 0.5% Triton X-100, 1 mM EDTA and 0.5% Na-Desoxycholat) and 500 µL PBS. The pulled-down proteins were eluted from the beads using 4x Laemmli buffer followed by incubation in Thermomixer at 25 °C for 20 min with 500 rpm shaking. Samples were boiled at 95 °C for 8 min and then loaded onto 10% Bolt Bis-Tris Plus gels (Thermo Fisher Scientific, Waltham, MA, USA) and run on XCellSureLock Mini Cell Electrophoresis System (Thermo Fisher Scientific, Waltham, MA, USA) until the sample front reaching 1 cm inside the running gel. Gels were then rinsed in water and fixed using a fixation solution (50% ethanol and 10% acetic acid in water) for 30 minutes. After removing the fixation solution, gels were incubated in the Coomassie solution (Serva, Heidelberg, Germany) for 4 hours while shaking. Gels were washed in ultrapure water overnight and submitted to Core Facility for Mass Spectrometry & Proteomics (CFMP) at the Zentrum für Molekulare Biologie der Universität Heidelberg (ZMBH) for tryptic digestion and LC/MS analysis. Data analysis was performed using MaxQuant software version 1.6.12.0 and Perseus software v2.0.3.0.

In vivo lateral tail vein injection

The setup and group size for mouse experiments were approved by the German Regional Council of Baden-Württemberg and all experiments were performed in accordance with the approved protocol and other relevant guidelines and regulations. Tumor burden was monitored by imaging using luminescence and animal health was scored according to the animal study protocol. Animals were sacrificed before maximal tumor burden was not reached. Animals were kept with an automated night and day rhythm. First, GB-d1 cells were stably transduced with pFUGW-Pol2-ffLuc2-eGFP lentivirus and the resulting GB-d1-GFP-Luc cells were subjected to FACS to select cells with low expression of GFP. For the first generation of lateral tail vein injection, 200,000 GB-d1-GFP-Luc cells in 300 µL PBS were injected into the lateral tail vein of 8-10 weeks female ATYM-Foxn1nu/nu nude mice (Charles River Laboratories, Sulzfeld, Germany). After 4 weeks, mice were sacrificed and GB-d1-GFP-Luc cells were isolated from the lungs. Mouse lungs were cut into small pieces and incubated with Earle's

Balanced Salt Solution medium (EBSS; Sigma-Aldrich, Taufkirchen, Germany) containing 2.5 mg/mL collagenase type IV (Thermo Fisher Scientific, Waltham, MA, USA) and 0.1 mg/mL DNAseI (Merck Millipore, Darmstadt, Germany) for 30 minutes with shaking at 37 °C. The supernatant was strained using a 100 µm cell strainer (Merck Millipore, Darmstadt, Germany), expanded and sorted for GFP positive cells by FACS. The resulting cells were then stably transduced with pLenti-CMV-Puro-Ctrl or pLenti-CMV-Puro-CEACAM6 plasmid and positively selected using Puromycin (2 µg/mL) for 3 days. To compare luciferase expression levels between transduced cells, firefly luciferase assays were performed according to Promega Dual-Luciferase® Reporter Assay (Promega,Madison, WI, USA). For the second generation of the injection, GB-d1-GFP-Luc-Ctrl or GB-d1-GFP-Luc-CEACAM6 cells were injected into the lateral tail vein of 8-10 weeks ATYM-Foxn1nu/nu nude mice (each group of 12 mice, 200,000 cells/mouse). To observe the tumor growth, mice were anesthetized using isoflurane (Baxter, Deerfield, IL, USA) and injected intraperitoneally with 200 µL of Xenolight D-luciferin (PerkinElmer, Waltham, MA, USA) and tumor growth was observed using the IVIS system. Mice were sacrificed and lungs were isolated 4 weeks post-injection.

Hematoxylin and eosin (HE) and immunohistochemical (IHC) staining

After overnight fixation in 10 % buffered formalin, representative specimens of the liver were routinely dehydrated, embedded in paraffin and cut into 3 µm-thick sections. Tissue sections were stained with hematoxylin and eosin (HE) according to standard protocols. Staining of Ku80 and Ki67 were performed using antibodies listed in Supplementary Table S10. Sections were pretreated by boiling for 10 min with pH 6.0 Buffer (S2031, Agilent) and incubated over night with the primary antibody, anti-Ku80 or anti Ki67, using a dilution of 1:100 and 1:500, respectively. An anti-rabbit secondary antibody conjugated to AP was applied (PolyviewPlus, ENZO Life Sciences GmbH, Lörrach, Germany) and the signal was visualized using alkaline phosphatase (Permanent AP Red, Zytomed Systems, Berlin, Germany) as chromogen. Stained slides were scanned using the NanoZoomer slide scanner and analyzed by QuPath digital software.

Tissue microarrays (TMA) and immunohistochemical staining

Tissue microarrays (TMA) followed by immunohistochemistry included 89 non-tumor (NT) gallbladder and 131 GBC tissue samples. Formalin-fixed paraffin-embedded (FFPE) tissue blocks were punched with 1 mm diameter and embedded into a new paraffin block by using a tissue microarrayer (TMA Grand Master fa. Sysmex, Germany). For immunohistochemical staining, 3 µm sections of the TMA were cut, deparaffinized and rehydrated. Then, immunohistochemical staining was carried out with an automated immunostainer (Ventana BenchMark ultra, Roche Diagnostics, Rotkreuz, Switzerland) using CEACAM5 antibody (TA803456S, Origene) and CEACAM6 antibody (sc-59899, Santa Cruz Biotechnology). For further steps, the biotin-free system OptiView DAB IHC Detection Kit (Ventana Medical Systems, AZ, USA) was used including OptiView Universal Linker, OptiView HRP Multimer and DAB-Chromogen. Finally, the slides were counterstained with hematoxylin. Stained slides were scanned using the NanoZoomer slide scanner and analyzed by QuPath digital software. Staining intensity was evaluated resulting in 4 different staining scores: score 0 (no staining), score 1 (weak staining), score 2 (moderate staining) and score 3 (strong staining). Further analysis of CEACAM5 and CEACAM6 staining intensity score in correlation with patient clinical status including overall survival and GBC staging based on UICC were performed.

Supplementary Figures

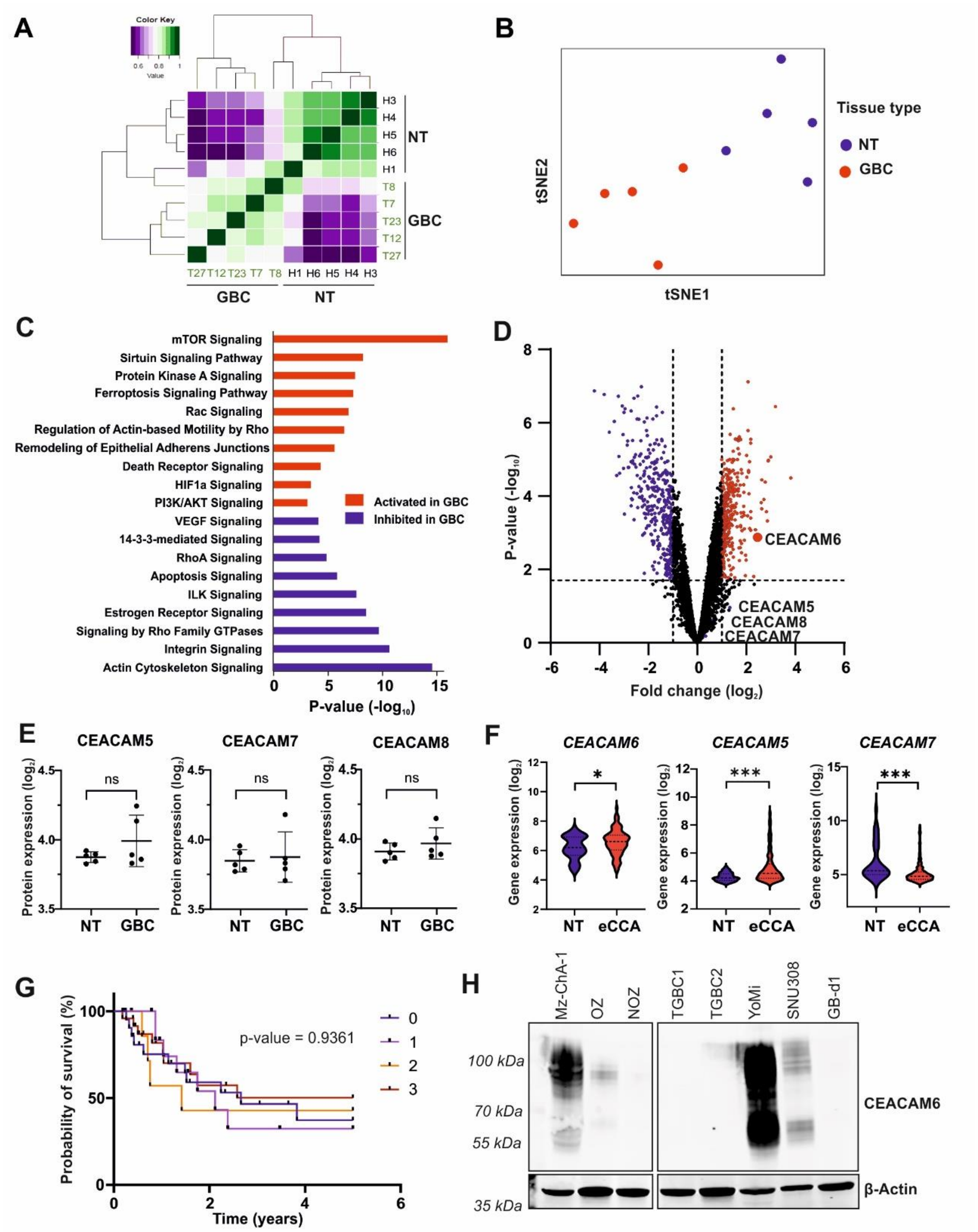

Figure S1. Expression profiling of CEACAM6 and CEACAM protein family members. (A) Heatmap of correlation plot and **(B)** t-distributed Stochastic Neighbor Embedding (t-SNE) analysis of the mass-spectrometry (MS)-based proteomics of 5 GBC and 5 non-tumor gallbladders (NT). **(C)** IPA of MS-based proteomics revealed up- and downregulated pathways related to GBC tumorigenesis. **(D)** Volcano plot highlighting the CEACAM family proteins and **(E)** dot plot of CEACAM5, CEACAM7 and CEACAM8 detected by MS analysis. P-values were determined by unpaired t-test (p≥0.05 ns, <0.05 *, <0.01 **, <0.001 ***). **(F)** Violin plots depicting gene expression of *CEACAM6*, *CEACAM5* and *CEACAM7* in extrahepatic

cholangiocarcinoma (eCCA, N=182) versus non-tumor bile duct (NT, N=38) of the GSE132305 dataset. P-values were determined by Mann-Whitney U test (p≥0.05 ns, <0.05 *, <0.01 **, <0.001 ***). **(G)** Kaplan-Meier curve showing the survival probability of patients with GBC (N=70) based on CEACAM6 staining intensity group with score 0 (N=19), score 1 (N=3), score 2 (N=19) and score 3 (N=29). P-value was determined by log-rank (Mantel-Cox) test. **(H)** CEACAM6 protein levels detected by Western blot in 8 different GBC cell lines. β-Actin served as loading control.

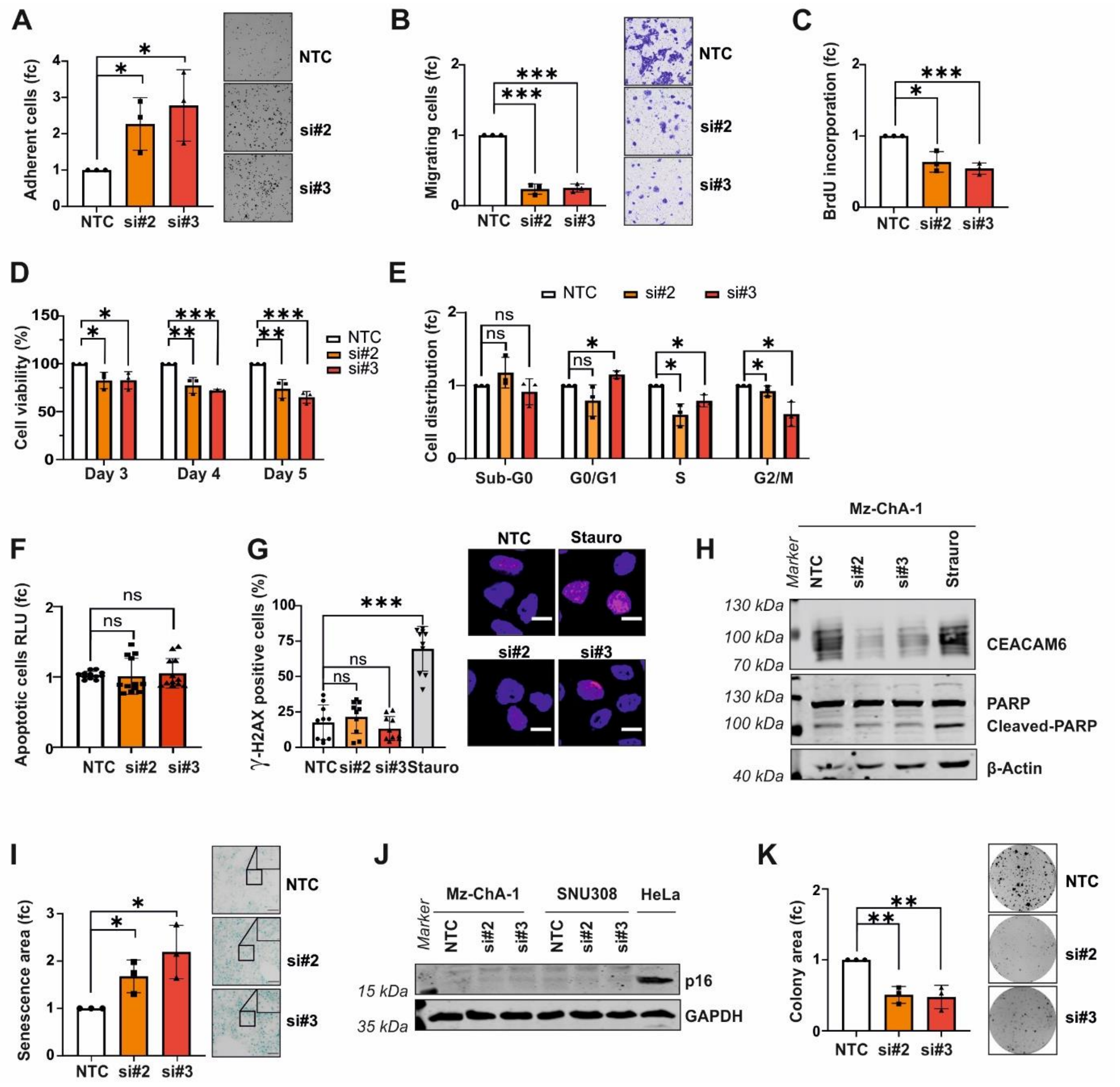

Figure S2. CEACAM6 knockdown inhibits GBC oncogenic functions. (A) Mz-ChA-1 cells were transiently transfected with two different siRNAs targeting CEACAM6. Three days after transfection, cells were used for adhesion assay and images of adherent Mz-ChA-1 cells 1 hour after seeding were captured and quantified. **(B)** From the transwell migration assay, the migrated Mz-ChA-1 cell area was calculated and representative images at 10x magnification are shown. **(C)** Bar graphs showing relative cell proliferation based on BrdU incorporation assay and **(D)** cell viability of Mz-ChA-1 cells after CEACAM6 knockdown on days 3, 4 and 5. **(E)** Cell cycle distribution in sub-G0, G0/G1, S and G2/M phases after CEACAM6 knockdown is depicted relative to NTC-transfected Mz-ChA-1 cells. **(F)** Quantification of apoptotic cells in Mz-ChA-1 cells after CEACAM6 knockdown based on Annexin V assay luminescence (RLU, relative light unit). **(G)** Percentage of γ-H2AX positive cells and representative immunofluorescence images of Mz-ChA-1 after CEACAM6 knockdown. The scale bar is 10 µm. Staurosporine (5 µM) treatment for 2 hours was used as positive control. **(H)** Western blot image of CEACAM6 and PARP protein after CEACAM6 knockdown or after two hours of Staurosporine (5 µM) treatment. β-Actin served as loading control. **(I)** The senescent cell area was captured based on β-galactosidase staining with 10x microscope magnification. Inset pictures show a detailed area and the scale bar is 20 µm. **(J)** Western blot image of p16 protein in Mz-ChA-1 and SNU308 after NTC or siCEACAM6 transfection, HeLa cell line was included

as positive control of p16 protein. GAPDH served as loading control. **(K)** Quantification and representative images of colony area after 14 days of CEACAM6 knockdown in Mz-ChA-1 cells. Data are represented as mean ± SD of three independent experiments. P-values were determined by unpaired t-test ($p \geq 0.05$ ns, <0.05 *, <0.01 **, <0.001 ***).

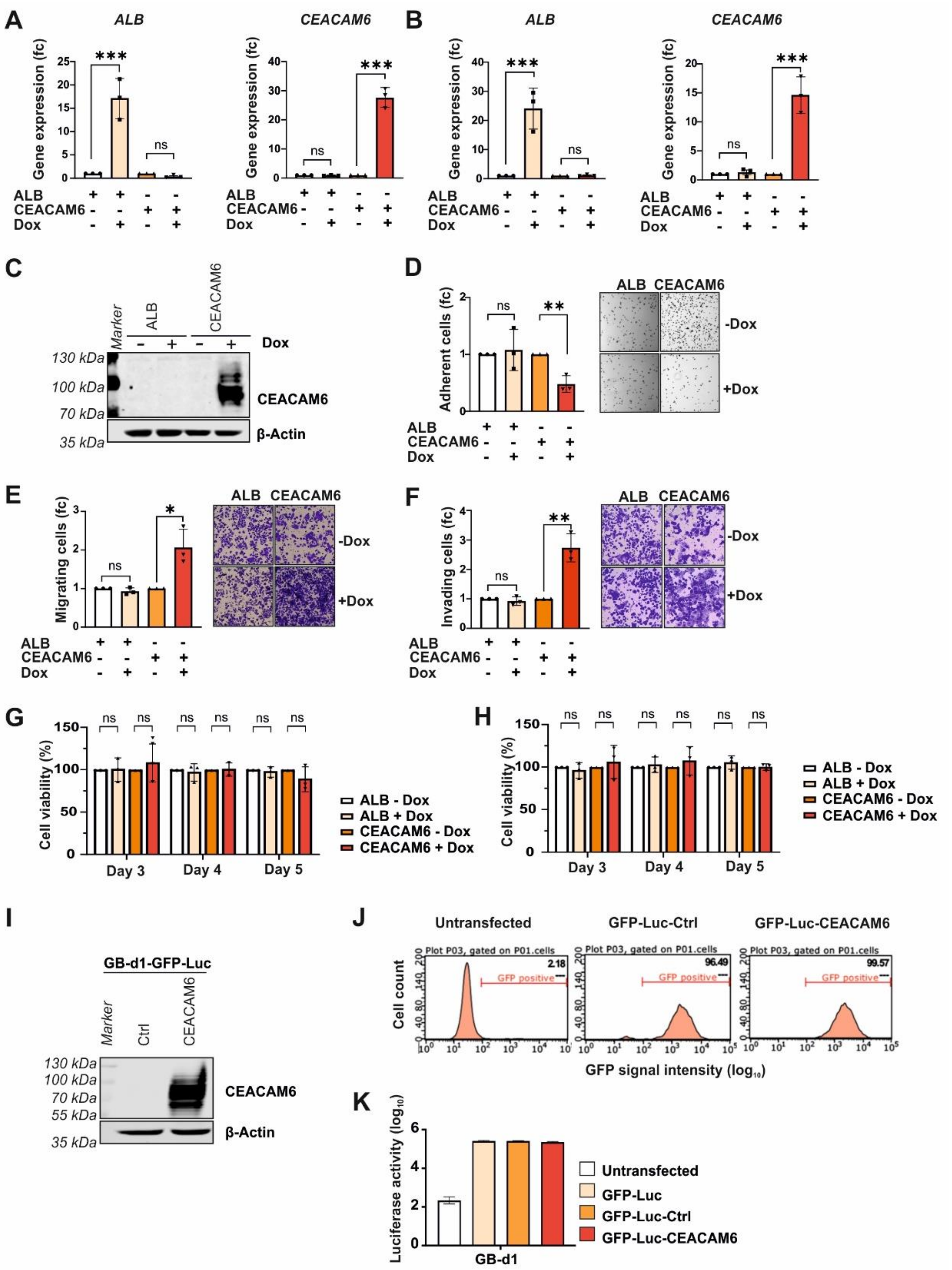

Figure S3. CEACAM6 regulates cell adhesion and initial step of cancer metastasis. (A) Analysis of ALB or CEACAM6 overexpression in GB-d1 and **(B)** TGBC1 cells with or without Doxycycline (Dox, 2 µg/mL) after transduction of inducible viral constructs by qPCR. SRSF4 gene expression was used as internal control for normalization. **(C)** Western blots of CEACAM6 overexpression in TGBC1 cells using inducible viral transduction with or without 2 µg/mL Dox treatment. β-Actin served as loading control. **(D)** Transwell migration and **(F)** invasion assays of TGBC1 were quantified and representative 10x magnification images are

shown. **(G)** Cell viability assay of GB-d1 and **(H)** TGBC1 with or without overexpression of ALB or CEACAM6 on days 3, 4 and 5. Data are represented as mean ± SD of three independent experiments. P-values were determined by unpaired t-test ($p \geq 0.05$ ns, <0.05 *, <0.01 **, <0.001 ***). **(I)** Western blot of CEACAM6 protein expression in GB-d1-GFP-Luc cells transduced with pLenti-CMV-Ctrl or pLenti-CMV-CEACAM6 and **(J)** FACS analysis showing the distribution of GFP-positive cells. **(K)** Quantification of luciferase activity normalized to protein concentration in GB-d1-GFP-Luc cells, as indicated. Shown are the mean $\log_{10}$ transformed relative values ± SD of two different cell passages.

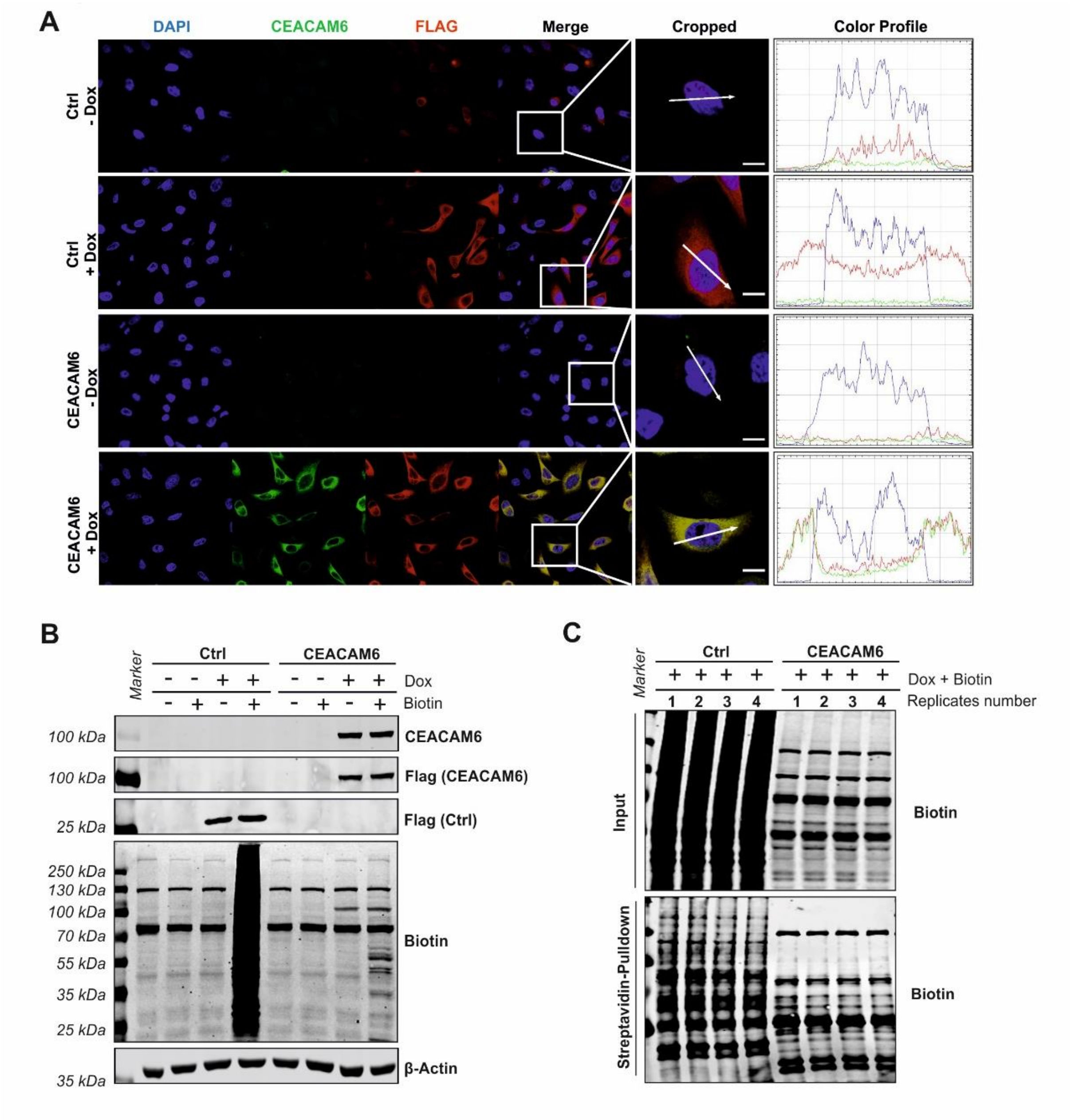

Figure S4. Validation of CEACAM6-C-BirA-Flag protein expression for CEACAM6 interacting partners elucidation through BioID. (A) Dox (2 µg/mL) induces the expression of Ctrl-BirA-Flag or CEACAM6-C-BirA-Flag protein in GB-d1 cells shown by representative immunofluorescence images. The arrows (30 µm long) in the magnified images represent intensity profiles of DAPI (blue signal), CEACAM6 (green signal) and FLAG-tag (red signal). The scale bar is 10 µm long. **(B)** Western blot of Ctrl-BirA-Flag or CEACAM6-C-BirA-Flag cells with or without Dox (2 µg/mL) and Biotin (50 nM) treatment. **(C)** Biotinylated proteins were detected with anti-Biotin antibody in input samples and after streptavidin pulldown. Four biological replicates were prepared for each construct and subjected to mass-spectrometric analysis.

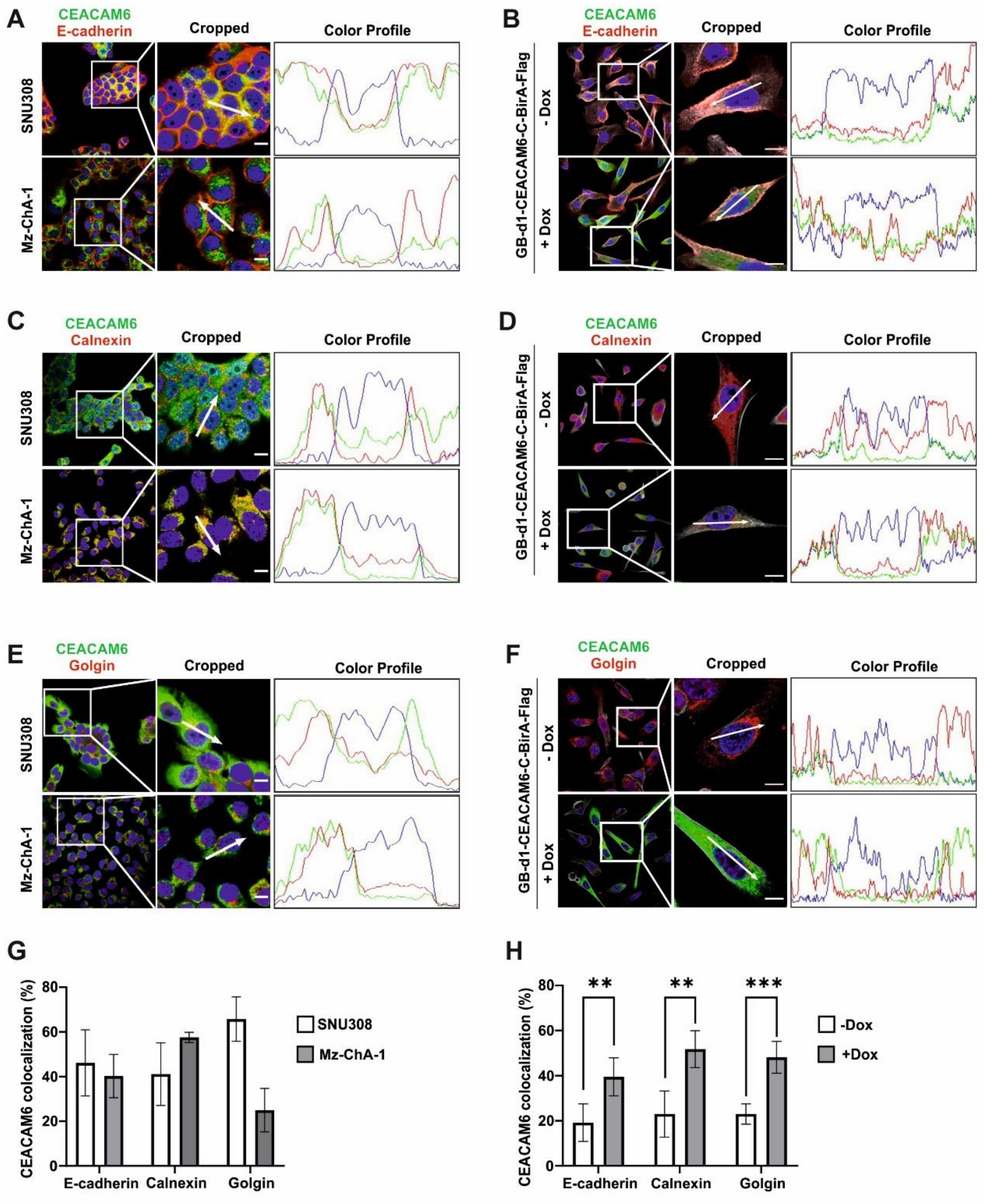

Figure S5. Subcellular localization of endogenous and overexpressed CEACAM6 based on immunofluorescence. Immunofluorescence images of endogenous CEACAM6 in SNU308 and Mz-ChA-1 or overexpressed CEACAM6 induced by Dox (2 µg/mL) treatment in GB-d1-CEACAM6-C-BirA-Flag are shown to colocalize with **(A,B)** cell membrane marker E-Cadherin, **(C,D)** endoplasmic reticulum marker Calnexin and **(E,F)** Golgi apparatus marker Golgin. Arrows (30 µm long) indicate the direction of color profile analysis with DAPI (blue signal), CEACAM6 (green signal) and E-Cadherin, Calnexin or Golgin (red signal), as indicated. The scale bar is 10 µm. **(G)** Quantification of CEACAM6 colocalization with E-cadherin, Calnexin and Golgin signals in SNU308 and Mz-ChA-1 cells and **(H)** in GB-d1-CEACAM6-BirA-Flag with or without CEACAM6 overexpression. Data are represented as mean ± SD of five different images. Percentage of colocalization was based on calculation of Mander's coefficient using the JaCoP plugin in FIJI software. P-values were determined by unpaired t-test ($p \geq 0.05$ ns, <0.05 *, <0.01 **, <0.001 ***).

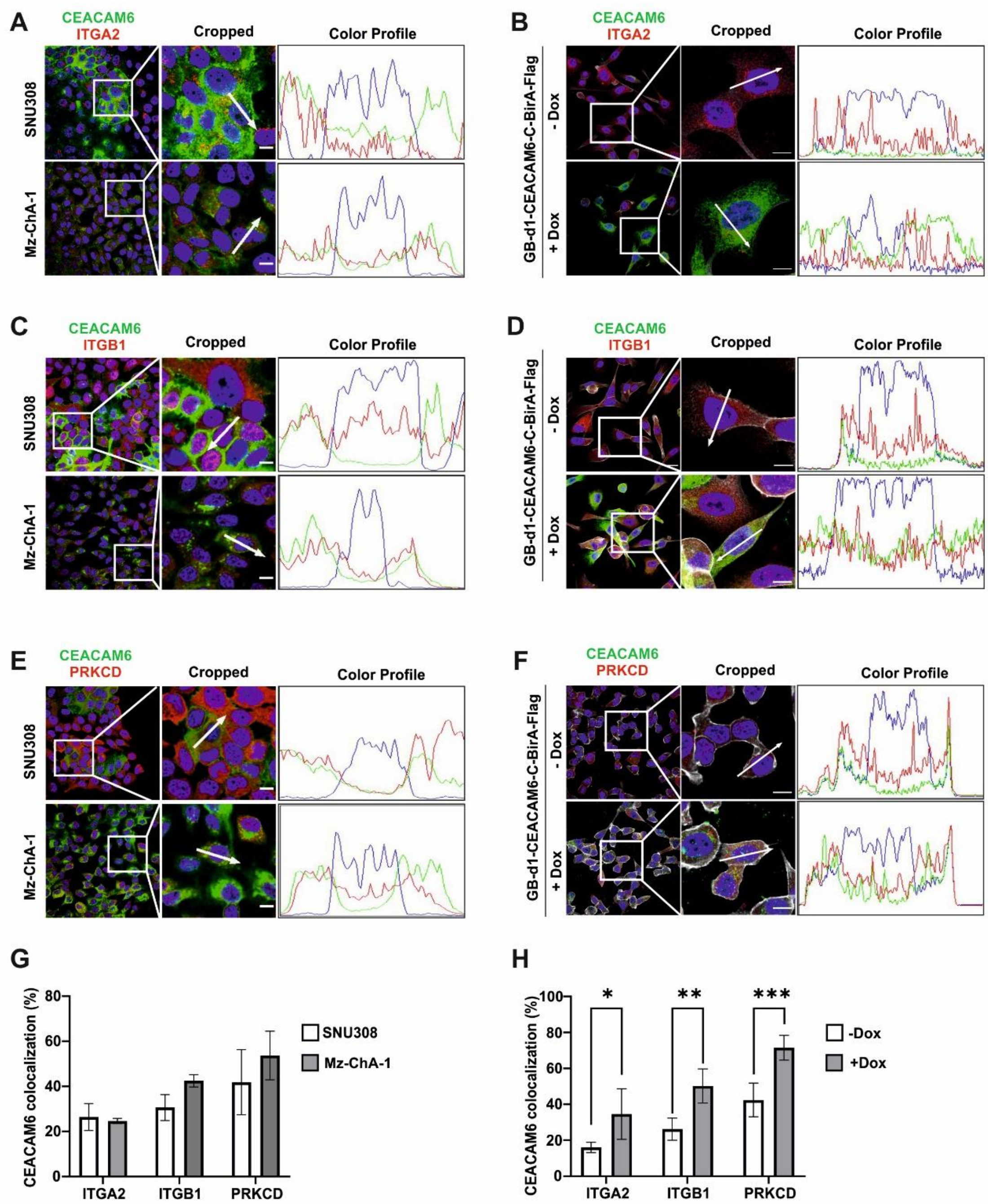

Figure S6. ITGA2, ITGB1 and PRKCD colocalize with CEACAM6 in subcellular locations. Immunofluorescence images of endogenous CEACAM6 in SNU308 and Mz-ChA-1 or overexpressed CEACAM6 induced by Dox (2 µg/mL) treatment in GB-d1-CEACAM6-C-BirA-Flag are shown to colocalize with CEACAM6 interaction partners **(A,B)** ITGA2, **(C,D)** ITGB1 and **(E,F)** PRKCD. Arrows (30 µm long) indicate the direction of color profile analysis with DAPI (blue signal), CEACAM6 (green signal) and ITGA2, ITGB1 or PRKCD (red signal), as indicated. The scale bar is 10 µm. **(G)** Quantification of CEACAM6 colocalization with ITGA2, ITGB1 and PRKCD signals in SNU308 and Mz-ChA-1 cells and **(H)** in GB-d1-CEACAM6-BirA-Flag with or without CEACAM6 overexpression. Data are represented as mean ± SD of five different images. Percentage of colocalization was based on calculation of Mander's coefficient using the JaCoP plugin in FIJI software. P-values were determined by unpaired t-test ($p \geq 0.05$ ns, <0.05 *, <0.01 **, <0.001 ***).

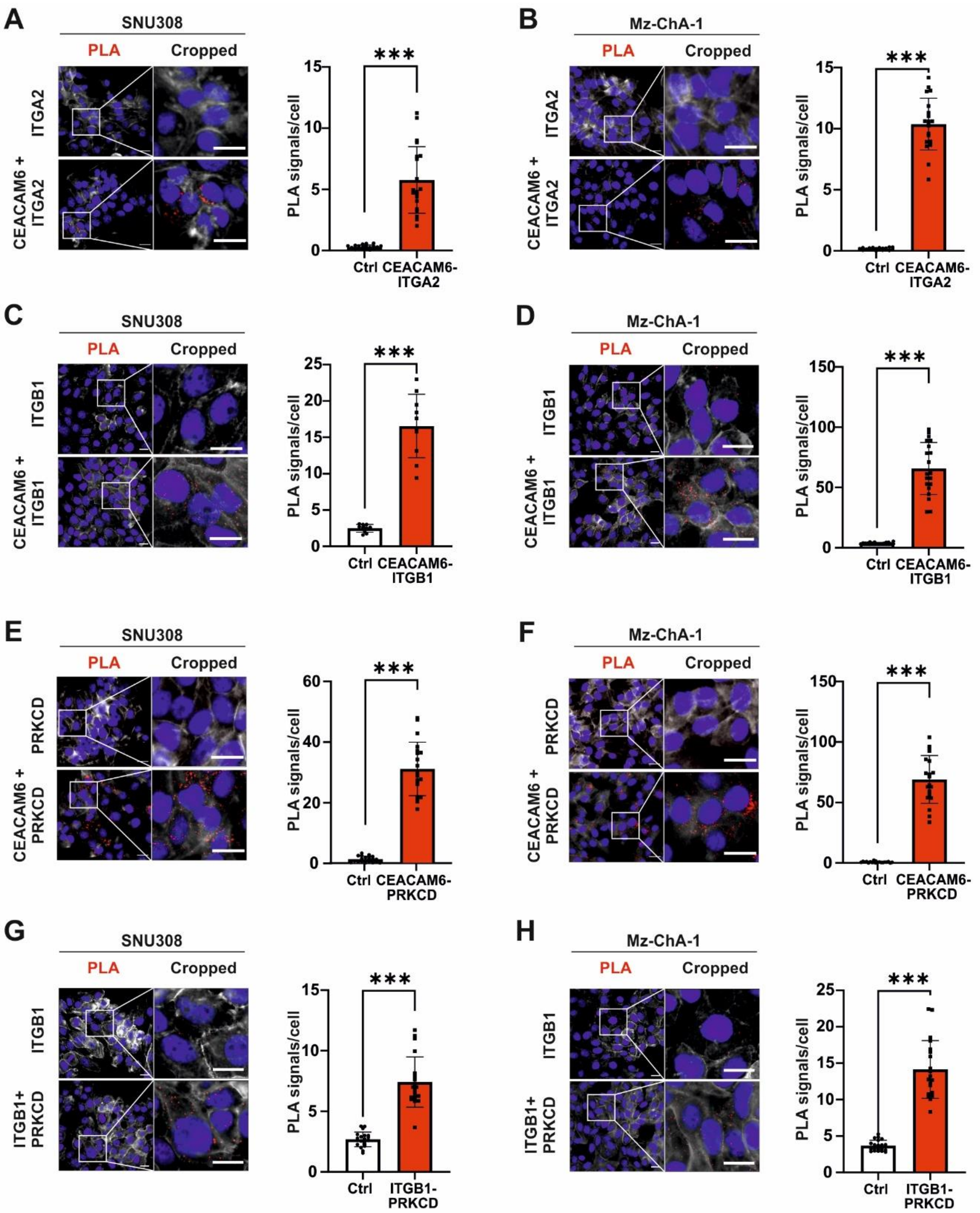

Figure S7. CEACAM6 interacts with ITGA2, ITGB1 and PRKCD in SNU308 and Mz-ChA-1 cells. (A,B) Immunofluorescence images from proximily ligation assay (PLA) showing direct interaction of CEACAM6 with ITGA2, **(C,D)** ITGB1 and **(E,F)** PRKCD, as well as **(G,H)** interaction of ITGB1 with PRKCD in SNU308 and Mz-ChA-1 cells. PLA images and quantification of PLA signals per cell from two independent replicates and 10 different image areas with 60x microscope magnification each are displayed. Error bars depict SD and p-values were determined by Mann-Whitney U test ($p < 0.001$ ***). The scale bar in the PLA picture is 10 µm.

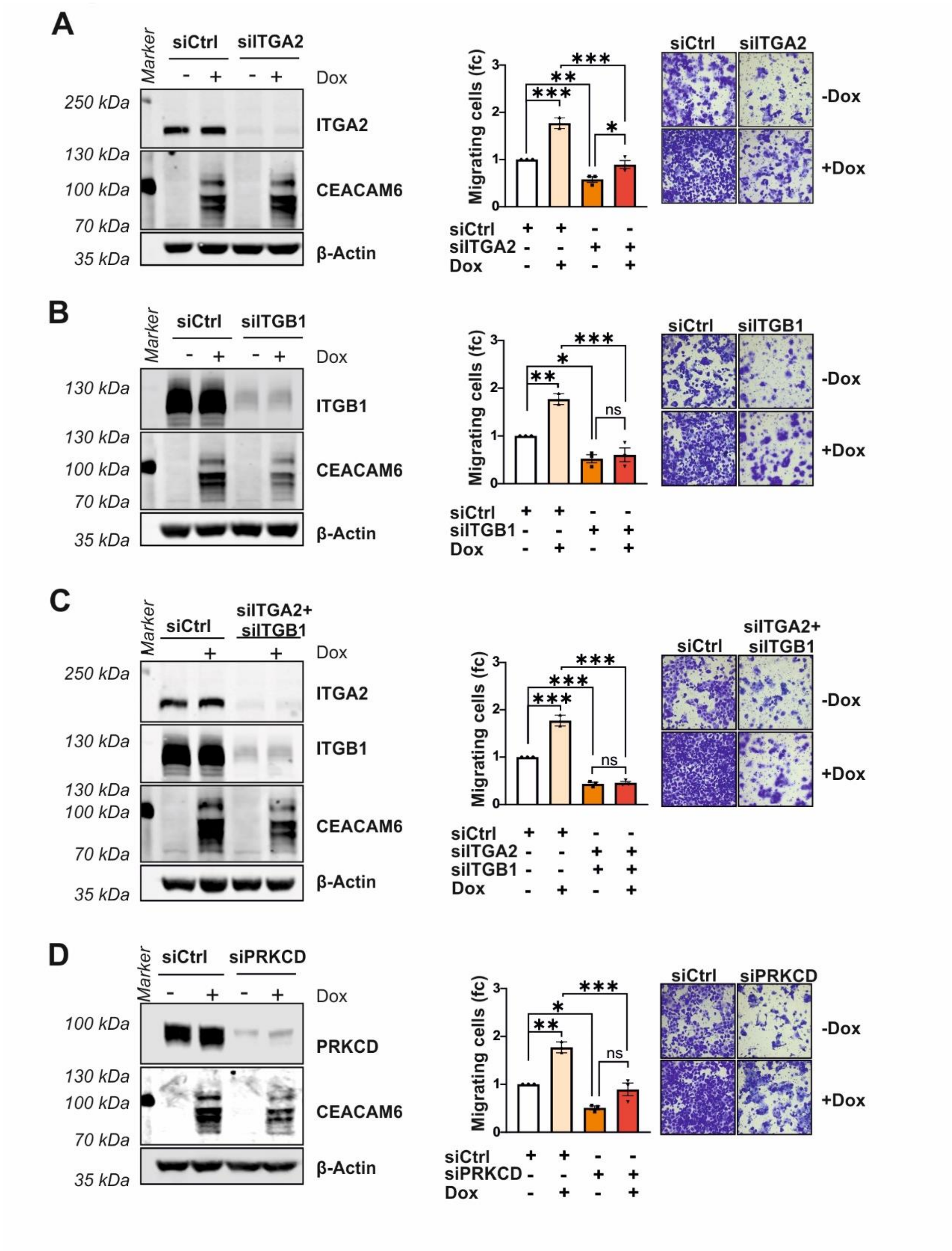

Figure S8. CEACAM6 collaborates with ITGB1 and PRKCD to regulate cell migration. (A) Western blots and transwell migration assays of TGBC1-CEACAM6 cells treated with Dox (2 µg/mL) to induce CEACAM6 overexpression and transfected with siPool (1 nM) to knockdown ITGA2, **(B)** ITGB1, **(C)** ITGA2 and ITGB1 together or **(D)** PRKCD. Values are the mean of three independent experiments with SD as error bars. P-values were determined by one-way ANOVA followed by Sidak's multiple comparisons test (p≥0.05 ns, <0.05 *, <0.01 **, <0.001 ***).

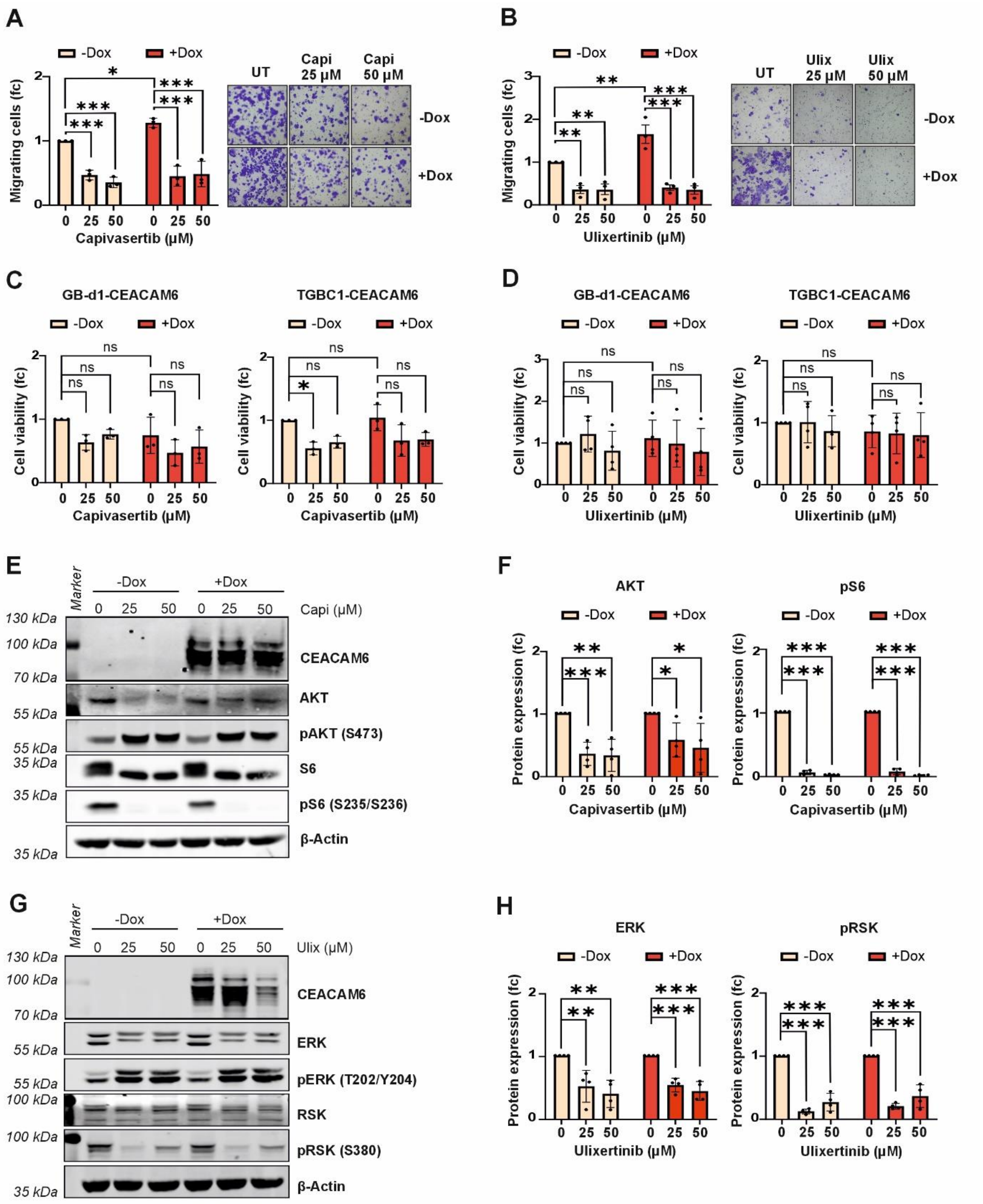

Figure S9. CEACAM6 function is inhibited by ERK and AKT inhibitors. (A) Cell migration transwell assays of TGBC1-CEACAM6 cells with or without CEACAM6 overexpression under capivasertib or **(B)** ulixertinib treatment for 24 hours. **(C)** Cell viability assay of GB-d1-CEACAM6 and TGBC1-CEACAM6 cells with or without CEACAM6 overexpression under capivasertib or **(D)** ulixertinib treatment for 24 hours. Data are represented as mean ± SD of three independent experiments. P-values were determined by two-way ANOVA followed by Sidak's multiple comparisons test (p≥0.05 ns, <0.05 *, <0.01 **, <0.001 ***). **(E)** Representative Western blot images of CEACAM6, AKT, pAKT (S473), S6 and pS6 (S235/S236) with or without CEACAM6 overexpression and capivasertib treatment for 24 hours in TGBC1-CEACAM6 cells. **(F)** Quantification of Western blot band density of AKT and pS6 after

capivasertib treatment in TGBC1-CEACAM6 for 24 hours. **(G)** Protein expression of CEACAM6, ERK, pERK (T202/Y204), RSK and pRSK (S380) are shown in representative Western blot images of TGBC1-CEACAM6 cells with or without CEACAM6 overexpression and ulixertinib treatment for 24 hours. **(H)** Quantification of Western blot band density of ERK and pRSK after ulixertinib treatment in TGBC1-CEACAM6 for 24 hours. β-Actin served as loading control and for normalization. P-values were determined by unpaired t-test ($p \geq 0.05$ ns, <0.05 *, <0.01 **, <0.001 ***).

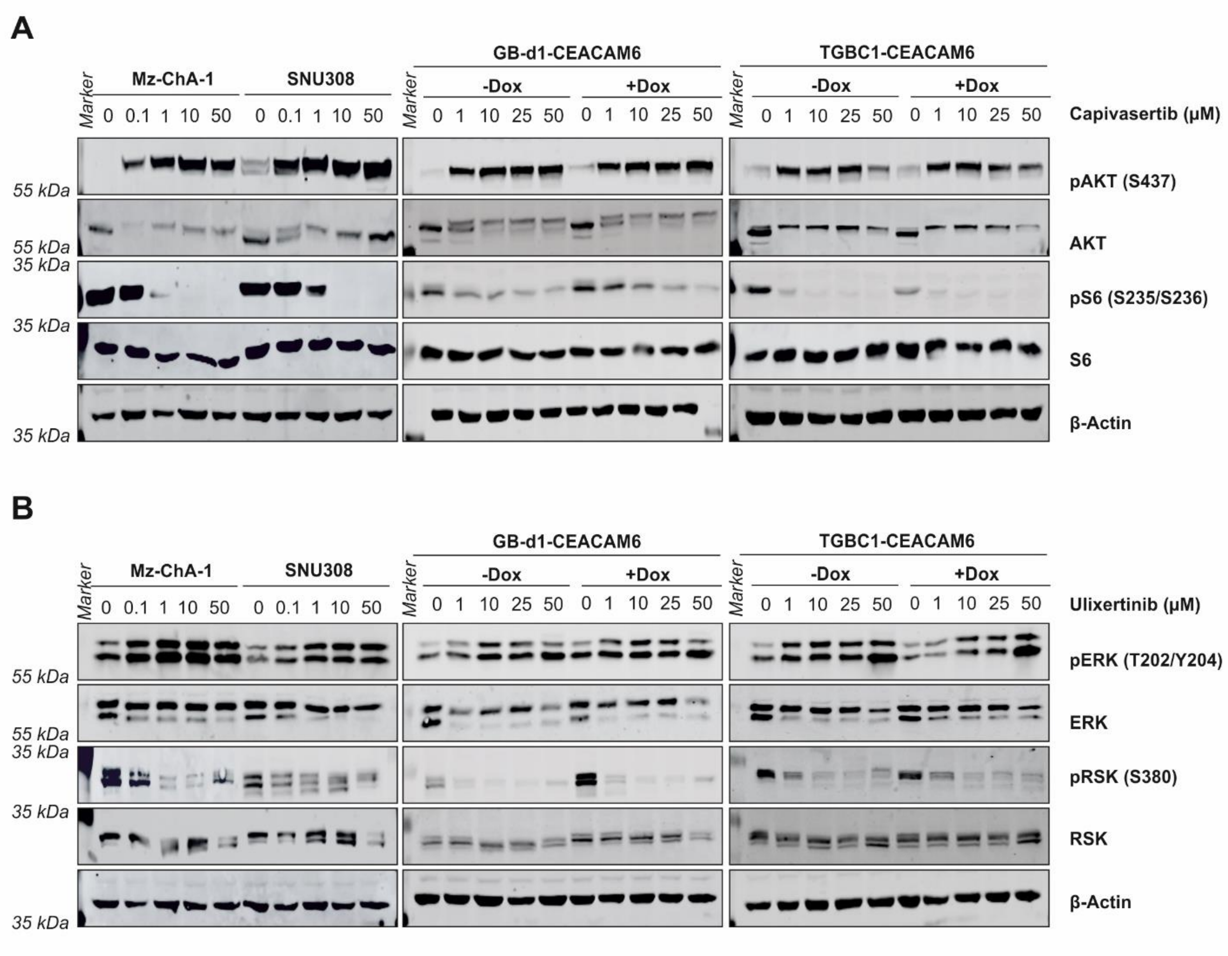

Figure S10. Dose titration of capivasertib and ulixertinib for AKT and ERK inhibition. (A) Western blot of Mz-ChA-1, SNU308, GB-d1-CEACAM6 and TGBC1-CEACAM6 cells detecting AKT, pAKT, S6 and pS6 after treatment with various concentraton of capivasertib and **(B)** detecting ERK, pERK, RSK and pRSK after treatment with various concentraton of ulixertinib for 24 hours. β-Actin served as loading control.

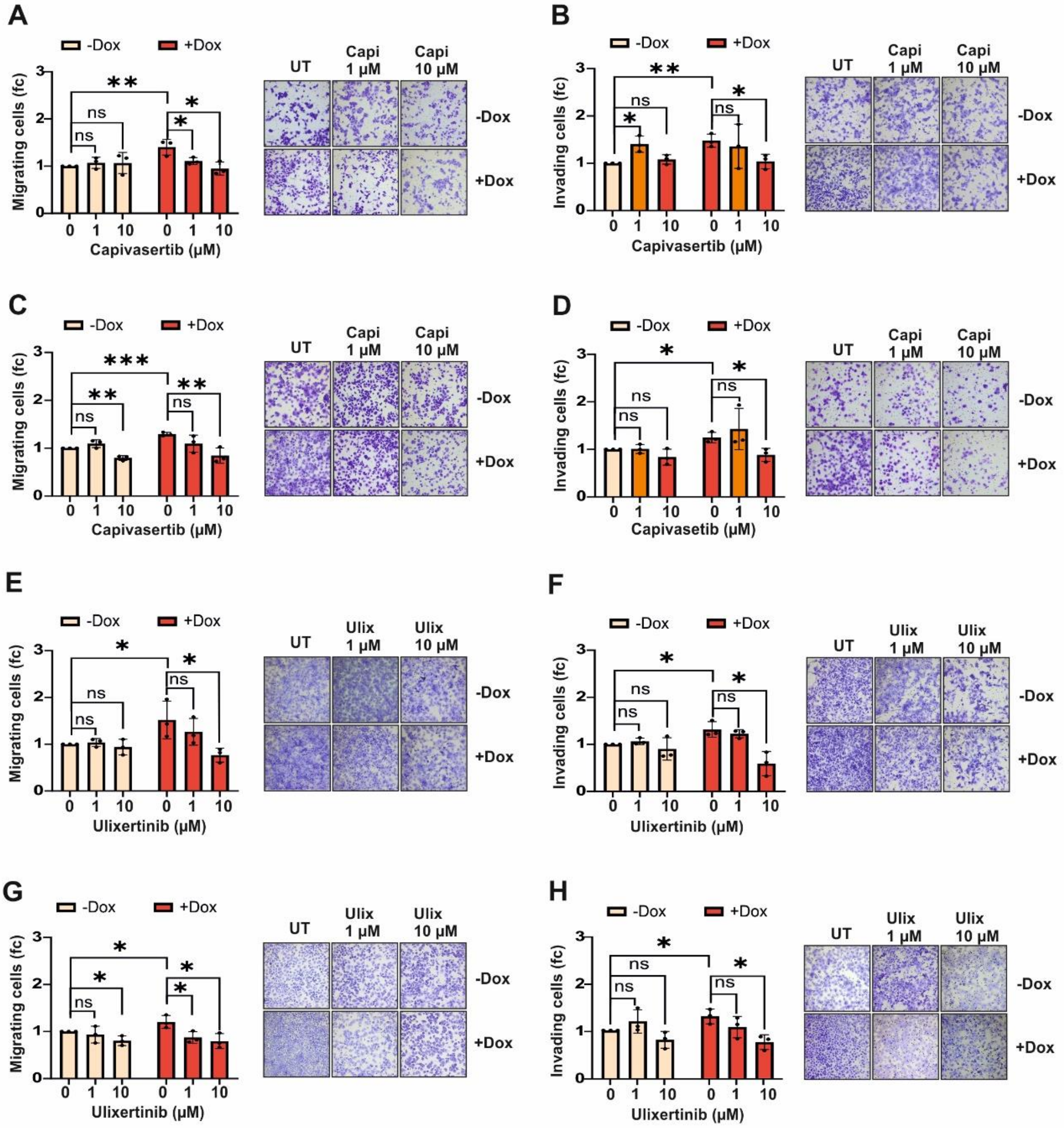

Figure S11. Capivasertib and ulixertinib treatment inhibit CEACAM6-induced migration and invasion. (A) Quantification and representative images of transwell migration and **(B)** invasion assay of GB-d1-CEACAM6, as well as **(C)** transwell migration and **(D)** invasion assay of TGBC1-CEACAM6 cells with or without CEACAM6 overexpression and 0, 1 or 10 µM capivasertib. **(E)** Quantification and representative images of tranwell migration and **(F)** invasion assay of GB-d1-CEACAM6, as well as **(G)** transwell migration and **(H)** invasion assay of TGBC1-CEACAM6 cells with or without CEACAM6 overexpression and 0, 1 or 10 µM ulixertinib. Values are the mean of three independent experiments with SD as error bars. P-values were determined by unpaired t-test ($p \geq 0.05$ ns, <0.05 *, <0.01 **, <0.001 ***).

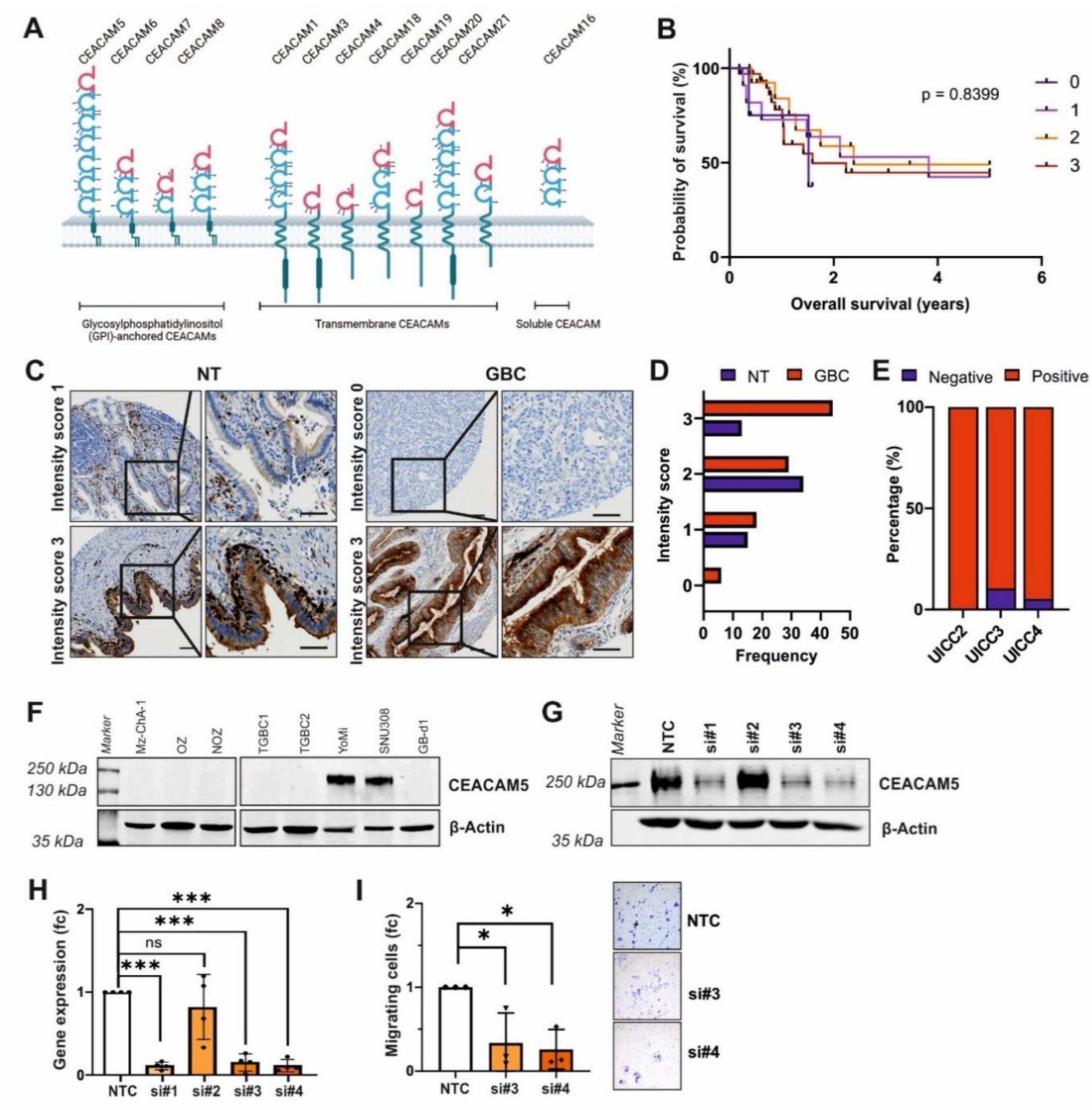

Figure S12. CEACAM5 is required for GBC cell migration. (A) Overview of CEACAM family structure, created with Biorender (https://app.biorender.com/) adapted from Johnson and Mahadevan and from Rizeq et al. [5, 6]. **(B)** Kaplan-Meier curve showing the survival probability of patients with GBC (N=63) based on CEACAM5 staining intensity group with score 0 (N=4), score 1 (N=11), score 2 (N=15) and score 3 (N=33). P-value was determined by log-rank (Mantel-Cox) test. **(C)** Representative images of CEACAM5 staining in non-tumor (NT) and GBC tissue samples with respective intensity scores. The scale bar is 50 µm. **(D)** Frequency of NT (N=62) or GBC (N=97) tissue samples with corresponding intensity score of CEACAM5 staining shown in a bar graph. Chi-square test χ^2=16.631, df=3 and p<0.001. **(E)** Bar graph depicting percentage of CEACAM5 staining status in GBC tissue samples for UICC2 (N=12), UICC3 (N=48) and UICC4 (N=19). Chi-square test χ^2=1.678, df=2 and p=0.4321. **(F)** Western blot of endogenous CEACAM5 in 8 GBC cell lines. **(G)** CEACAM5 protein and **(H)** RNA levels upon transient transfection of 4 siRNAs targeting CEACAM5. SRSF4 gene expression was used for normalization. **(I)** Quantification of migrated SNU308 cells after CEACAM5 knockdown and representative images of 10x magnification are shown. Data are

represented as mean ± SD of independent experiments. P-values were determined by unpaired t-test ($p \geq 0.05$ ns, <0.05 *, <0.01 **, <0.001 ***).

Supplementary Tables

Table S1. Patient characteristics.

Patient no.	Sex	Age (years)	pT	pN	L	M	V	G	R	UICC	Pre-existing condition
1	f	70.0	T2	N1	L1	M0	V0	G2	R0	UICC3	Cholezystolithiasis
2	m	83.9	T2	NA	L0	M0	V0	G2	R0	NA*	None
3	m	63.6	T3	NA	L0	M0	V0	G3	R1	NA*	Cholezystolithiasis
4	m	41.1	T4	NA	L1	M0	V0	G2	R0	UICC4	PSC
5	f	68.0	T4	N0	L0	M0	V0	G2	R1	UICC4	Cholezysto-/Choledocholithiasis

NA: not available

*Due to missing lymph node status, UICC could not be determined.

Supplementary Tables S2-S10 are provided online in separate Excel files.

Table S2: Comparison of protemic profiles of GBC versus non-cancerous normal (NT) gallbladder.

Table S3: Ingenuity Pathway Analysis (IPA) of differentially expressed proteins in GBC.

Table S4: Analysis of publicly available datasets.

Table S5: RNA sequencing data of CEACAM6 knockdown in SNU308.

Table S6: Ingenuity Pathway Analysis (IPA) of differentially expressed genes upon CEACAM6 knockdown in SNU308.

Table S7: RNA sequencing data of CEACAM6 overexpression in GB-d1.

Table S8: Ingenuity Pathway Analysis (IPA) of differentially expressed genes upon CEACAM6 overexpression in GB-d1.

Table S9: Mass-spectrometric detection of CEACAM6 interaction partners in BioID.

Table S10: List of materials

References

1. Day CP, Carter J, Bonomi C, Esposito D, Crise B, Ortiz-Conde B*, et al.* Lentivirus-mediated bifunctional cell labeling for in vivo melanoma study. *Pigment Cell Melanoma Res* 2009, **22**(3)**:** 283-295.

2. TW HB, Girke T. systemPipeR: NGS workflow and report generation environment. *BMC Bioinformatics* 2016, **17:** 388.

3. Bray NL, Pimentel H, Melsted P, Pachter L. Near-optimal probabilistic RNA-seq quantification. *Nat Biotechnol* 2016, **34**(5)**:** 525-527.

4. Ritchie ME, Phipson B, Wu D, Hu Y, Law CW, Shi W*, et al.* limma powers differential expression analyses for RNA-sequencing and microarray studies. *Nucleic acids research* 2015, **43**(7)**:** e47.

5. Johnson B, Mahadevan D. Emerging Role and Targeting of Carcinoembryonic Antigen-related Cell Adhesion Molecule 6 (CEACAM6) in Human Malignancies. *Clin Cancer Drugs* 2015, **2**(2)**:** 100-111.

6. Rizeq B, Zakaria Z, Ouhtit A. Towards understanding the mechanisms of actions of carcinoembryonic antigen-related cell adhesion molecule 6 in cancer progression. *Cancer Sci* 2018, **109**(1)**:** 33-42.
