# Supplementary material for: Proteomic profiling reveals CEACAM6 function in driving gallbladder cancer aggressiveness through integrin receptor, PRKCD and AKT/ERK signaling
Source: Cell Death Dis. 2024 Oct 28;15(10):780. doi: 10.1038/s41419-024-07171-x (PMC11519453; doi:10.1038/s41419-024-07171-x)

Figure 2A

Knockdown of CEACAM6

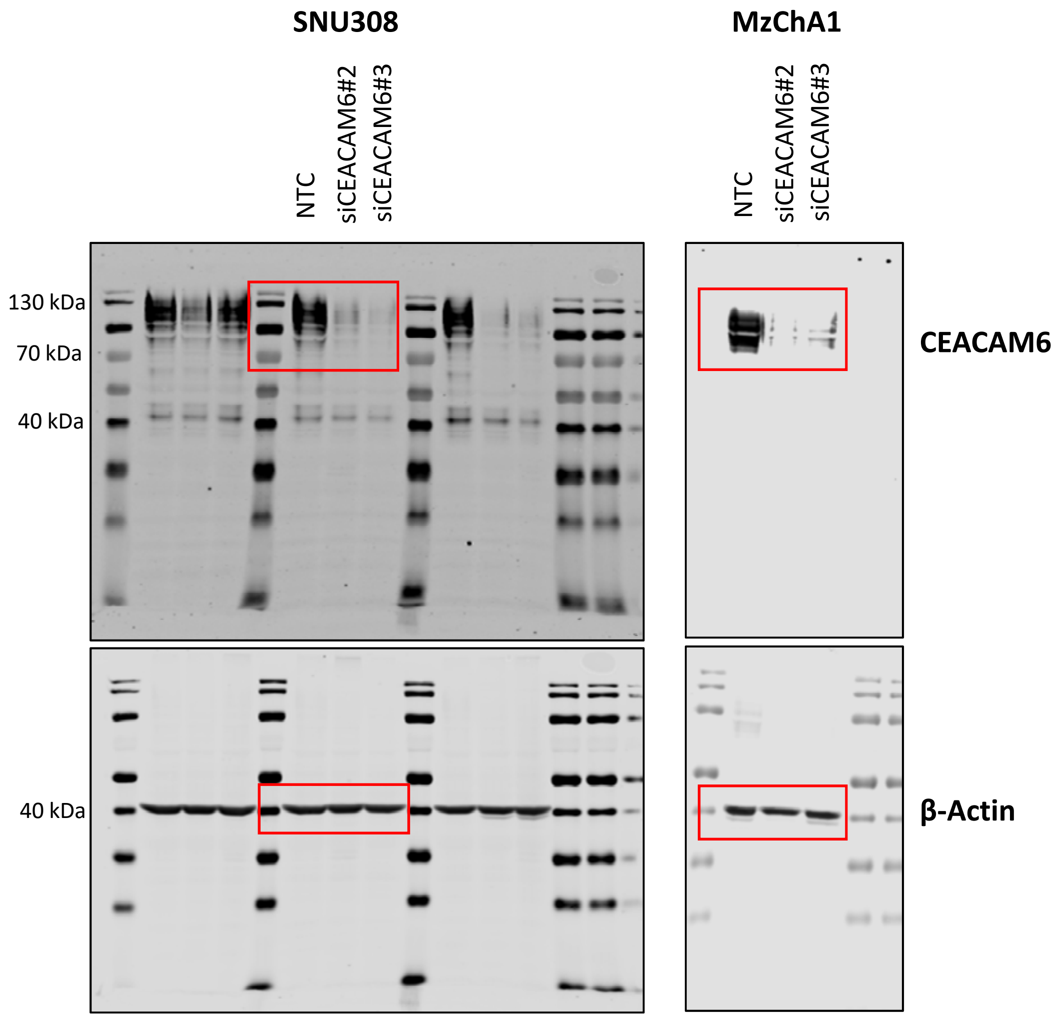

Figure 2J

PARP cleavage after siCEACAM6

SNU308

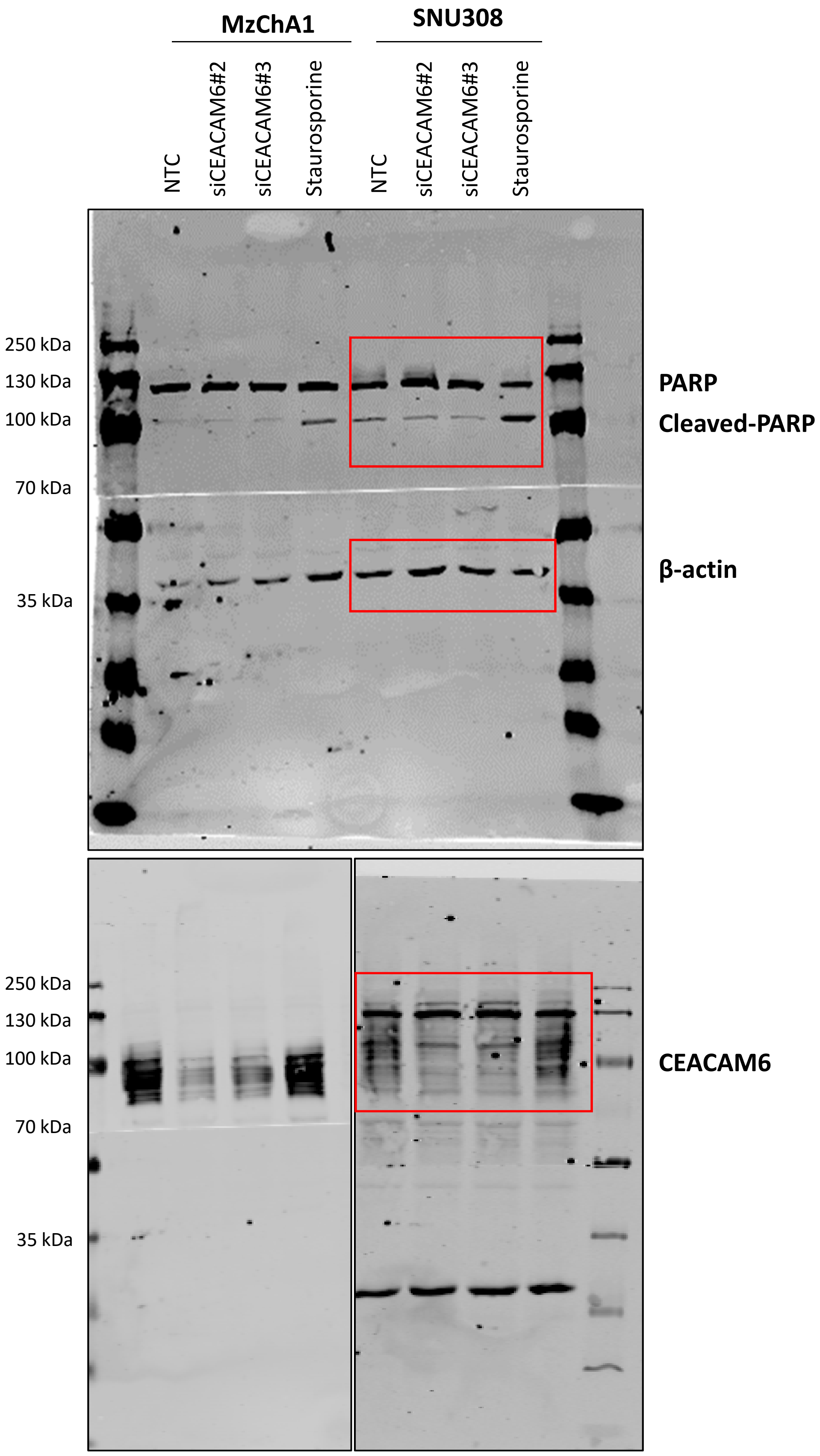

Figure 3F

AKT and ERK signaling after CEACAM6 knockdown

SNU308

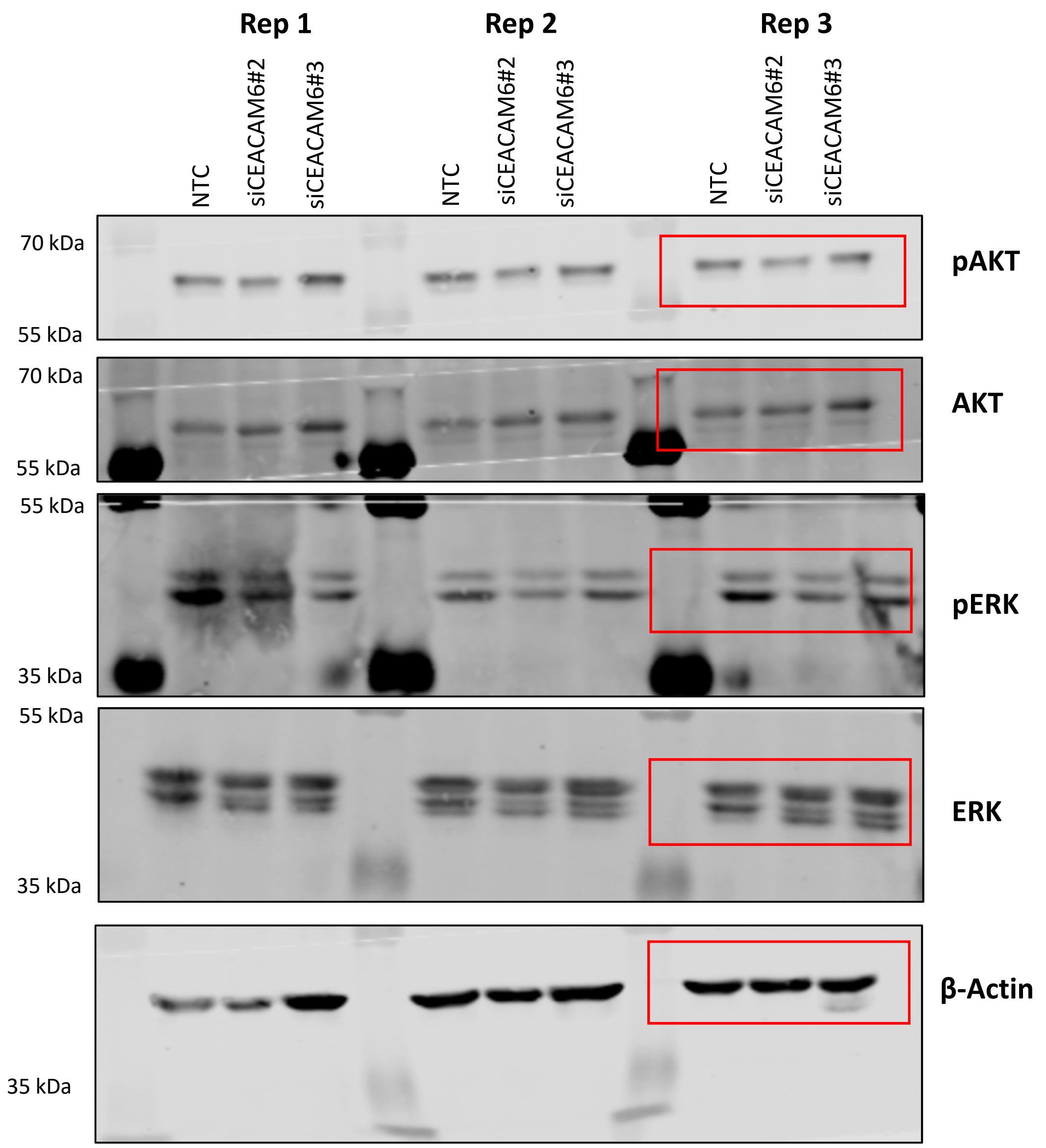

Figure 3F

AKT and ERK signaling after CEACAM6 knockdown

MzChA1

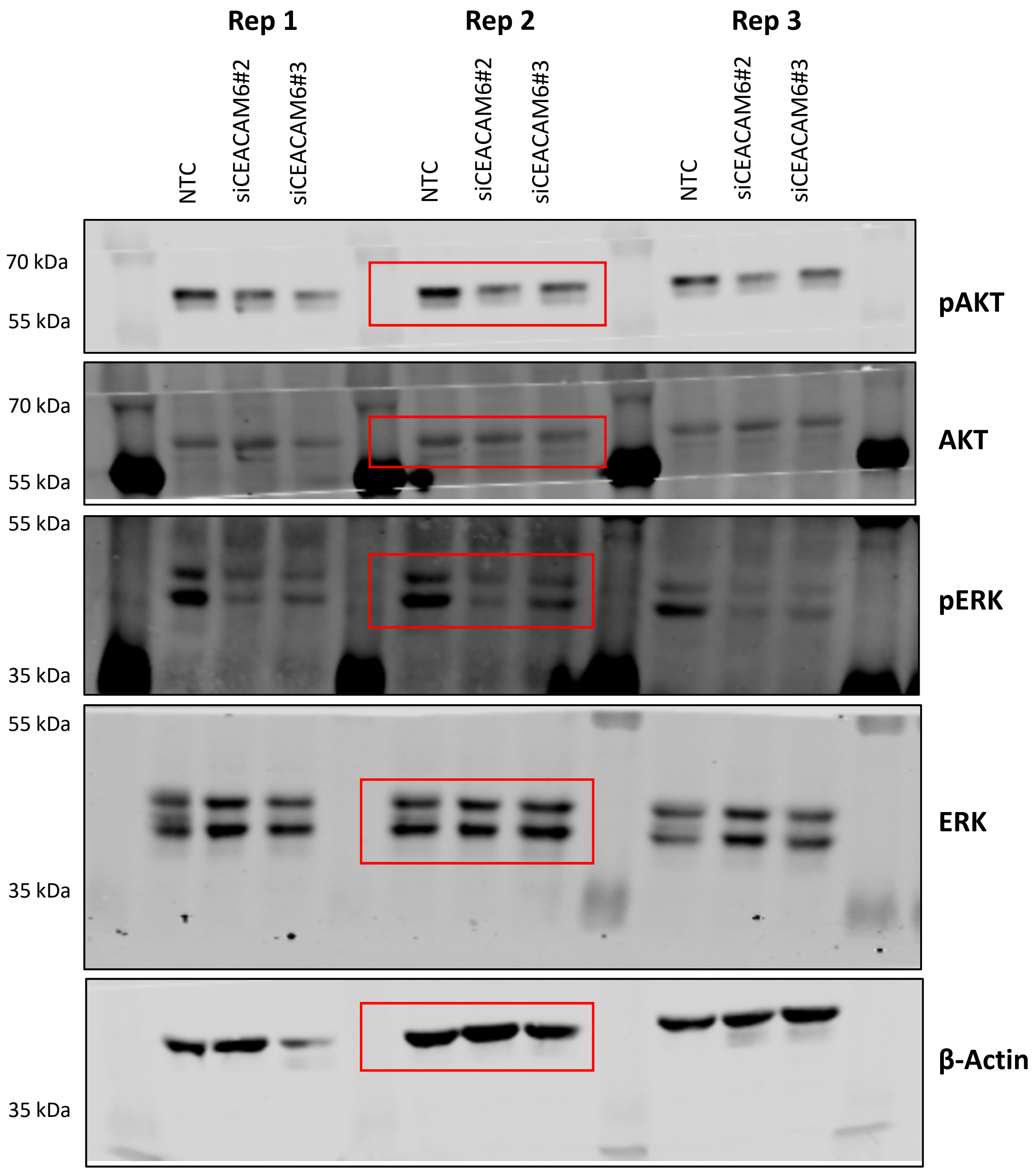

Figure 4A

CEACAM6 Overexpression

GB-d1

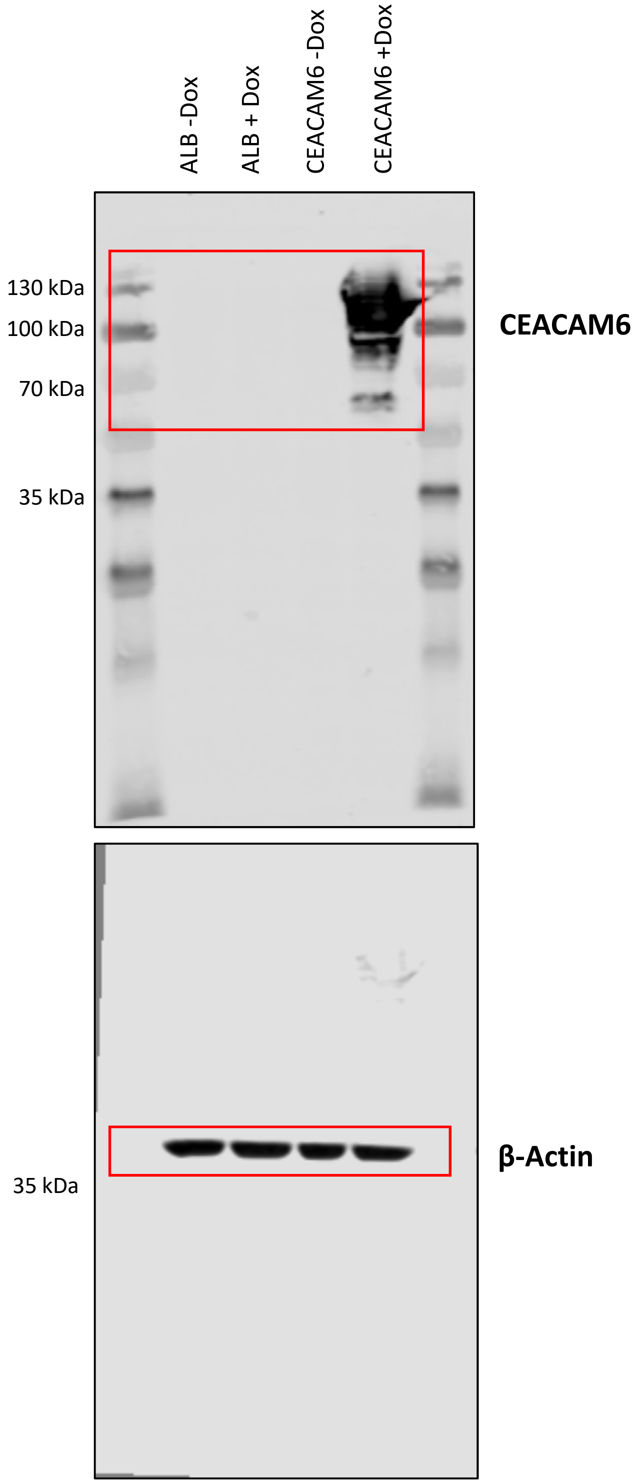

Figure 6G

CoIP CEACAM6 vs ITGA2

HEK

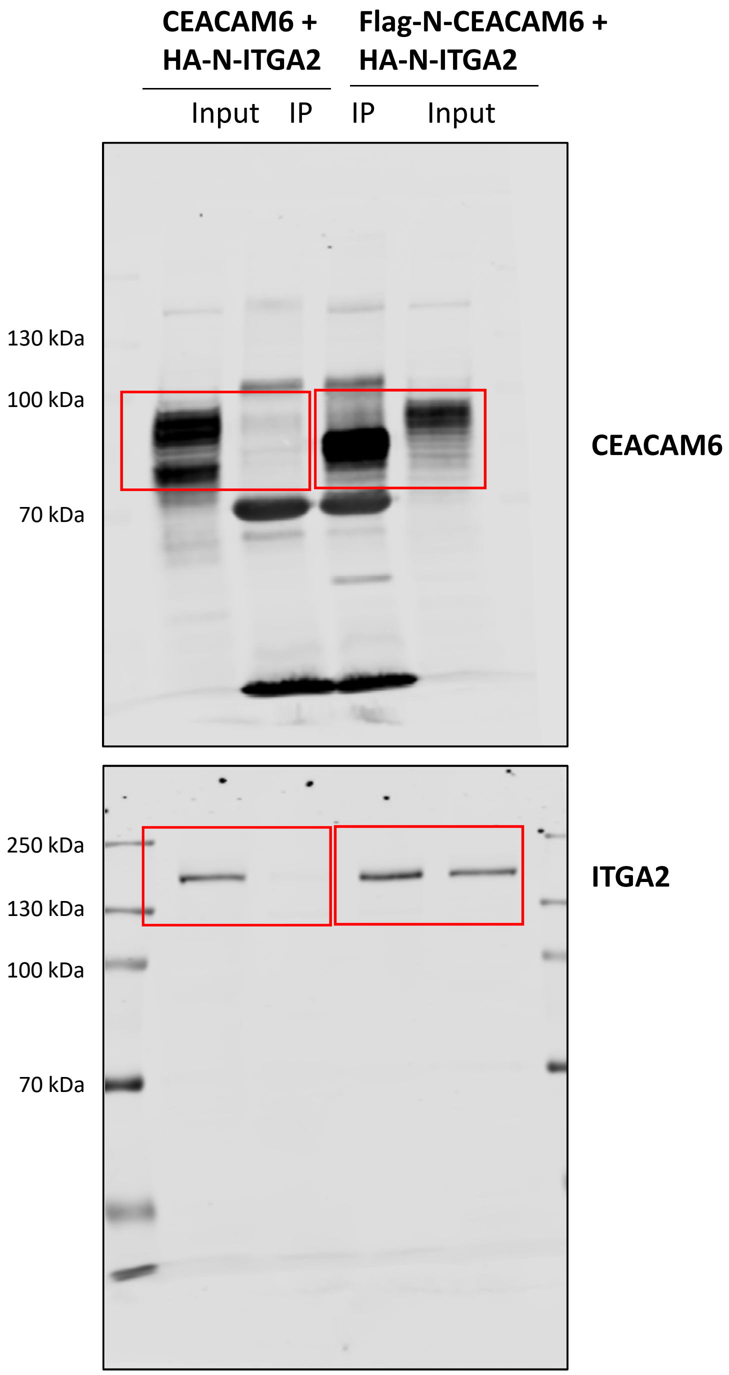

Figure 6H

CoIP CEACAM6 vs ITGB1

HEK

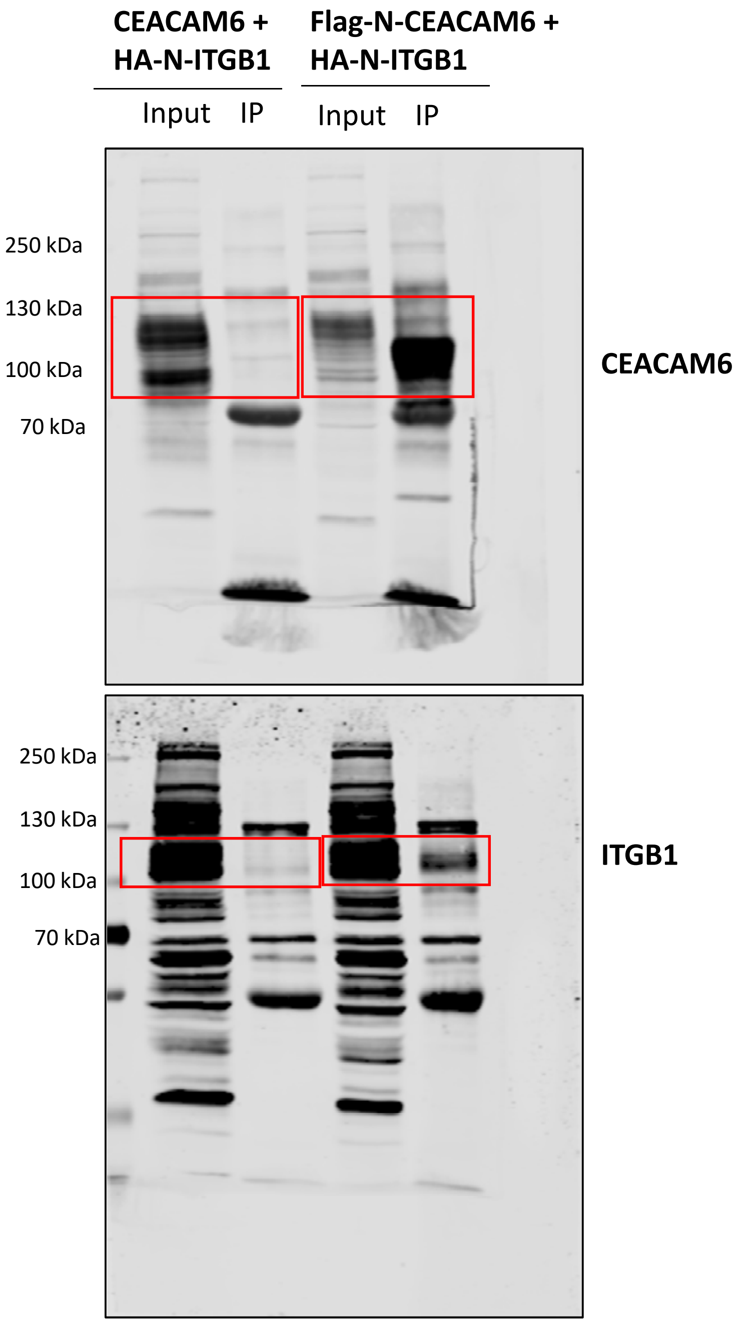

Figure 6I

CoIP CEACAM6 vs PRKCD

HEK

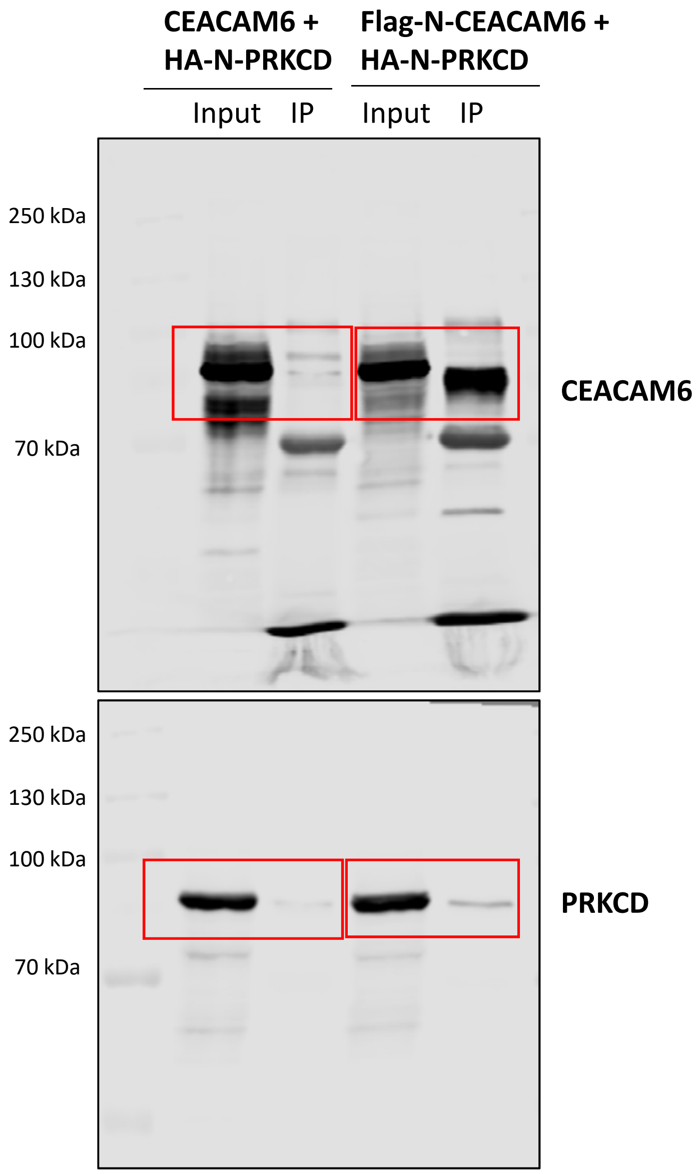

Figure 6J

CoIP CEACAM6 vs ITGB1 + PRKCD

HEK

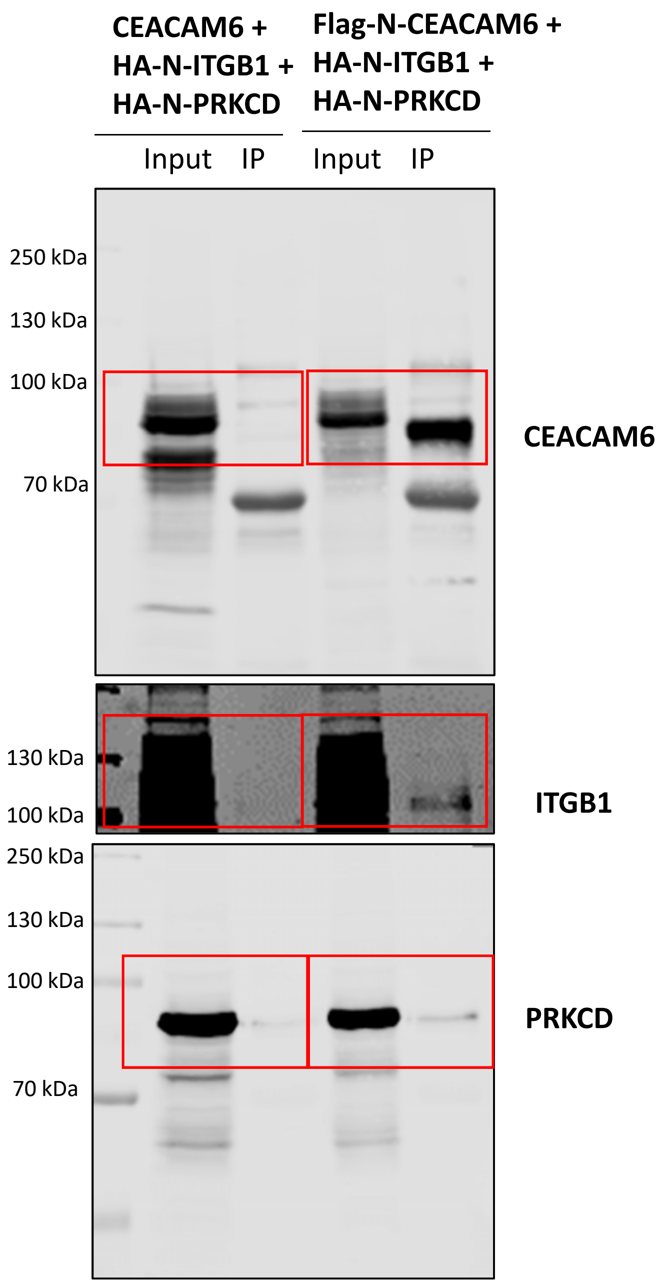

Figure 7A

siITGA2

GB-d1-CEACAM6

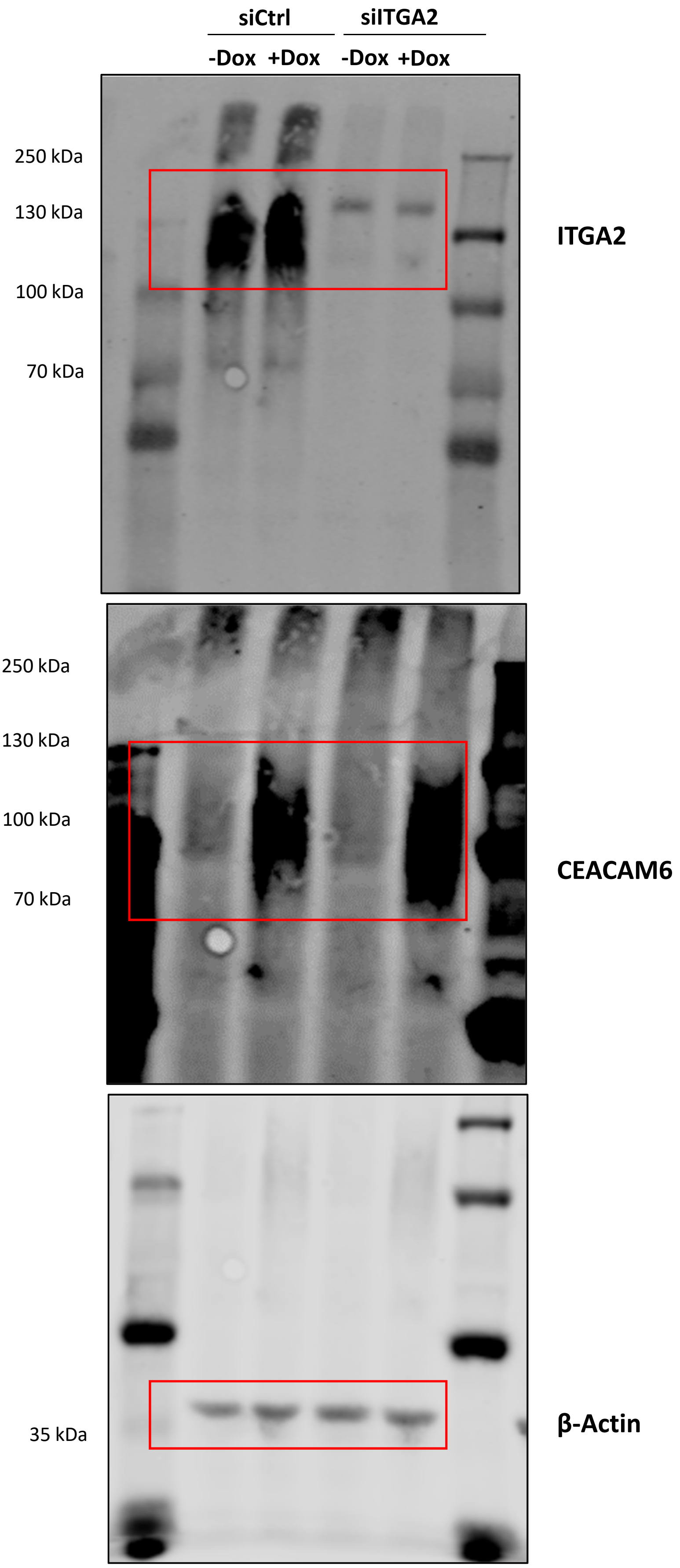

Figure 7B

siITGB1

GB-d1-CEACAM6

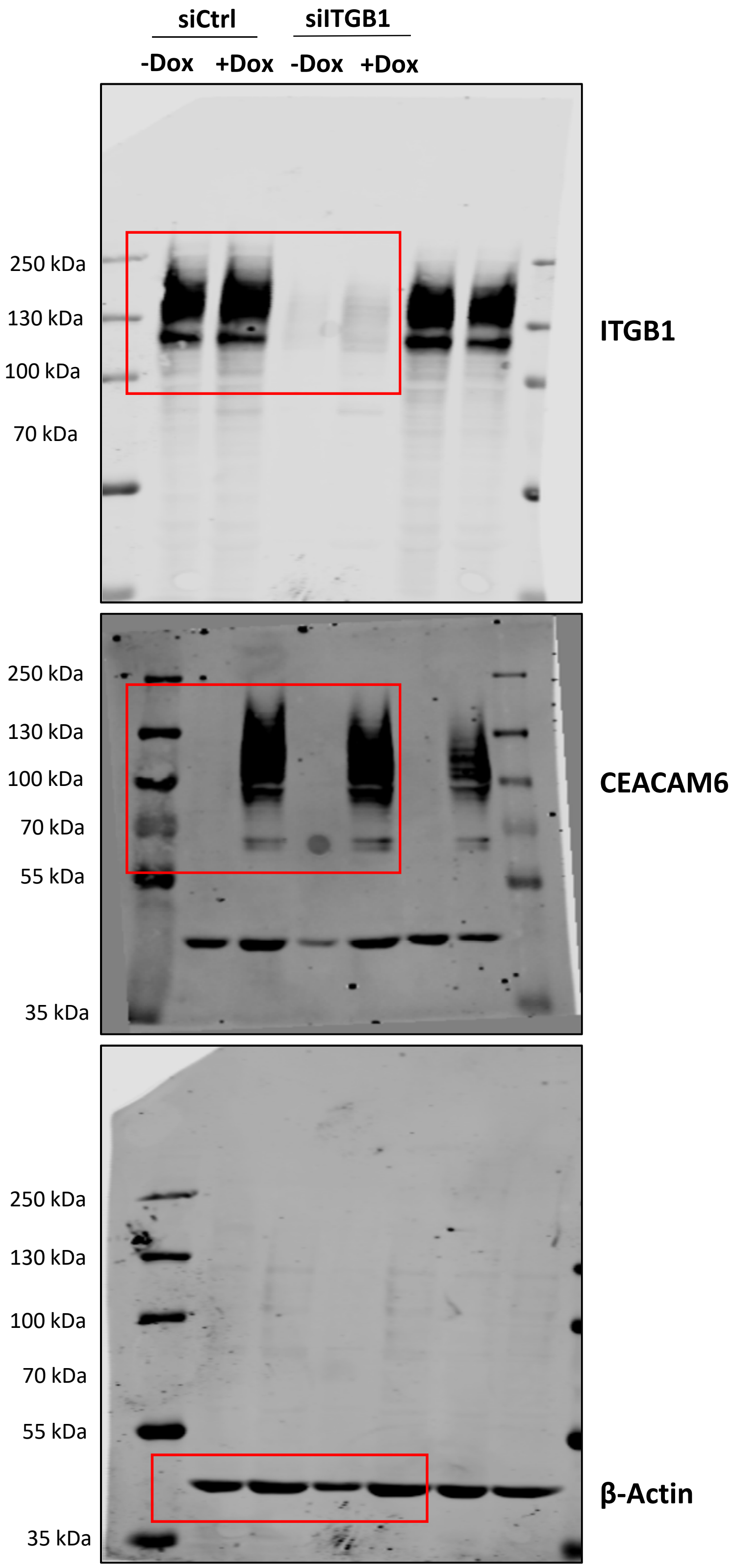

Figure 7C

siITGA2 + siITGB1

GB-d1-CEACAM6

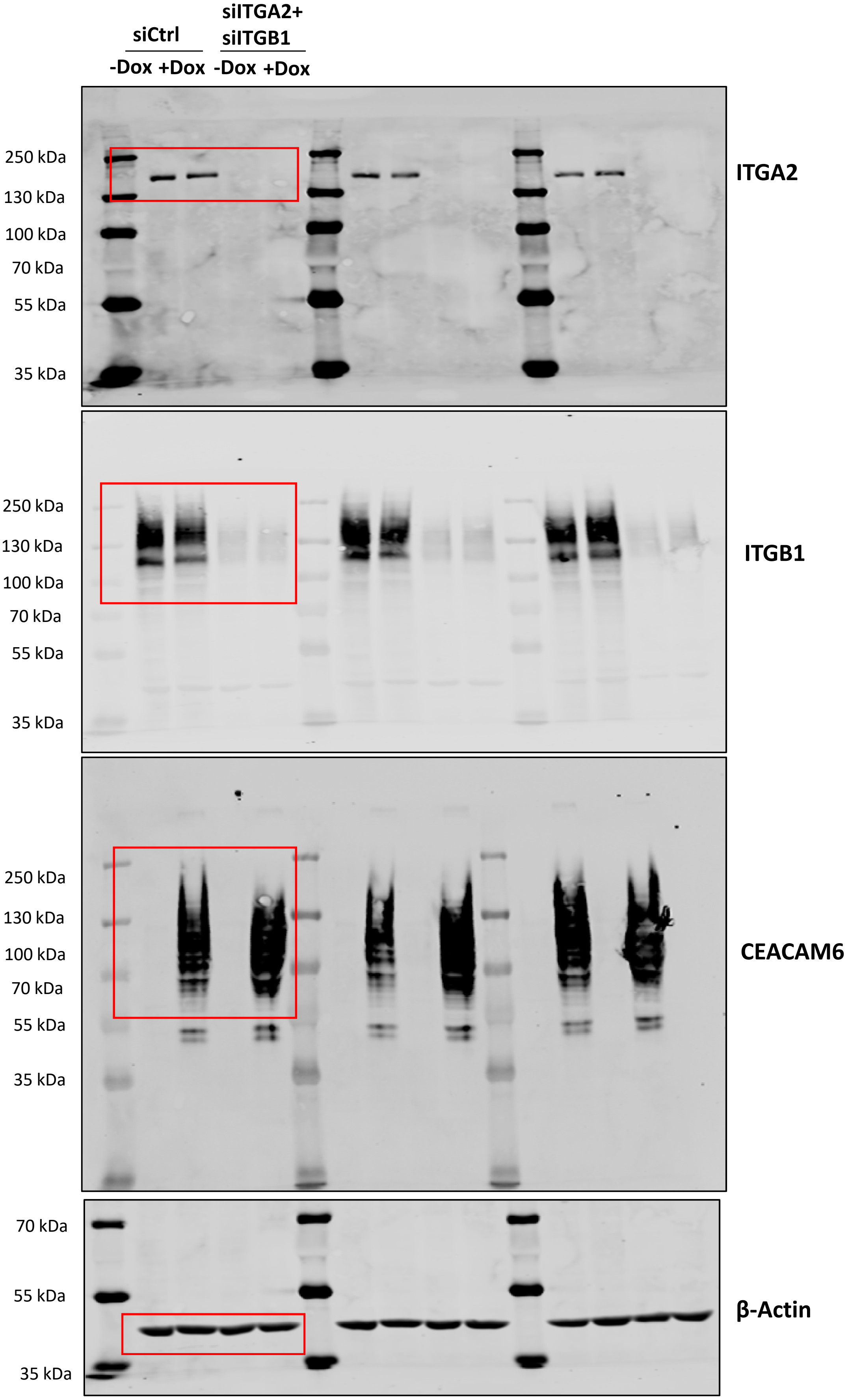

Figure 7D

siPRKCD

GB-d1-CEACAM6

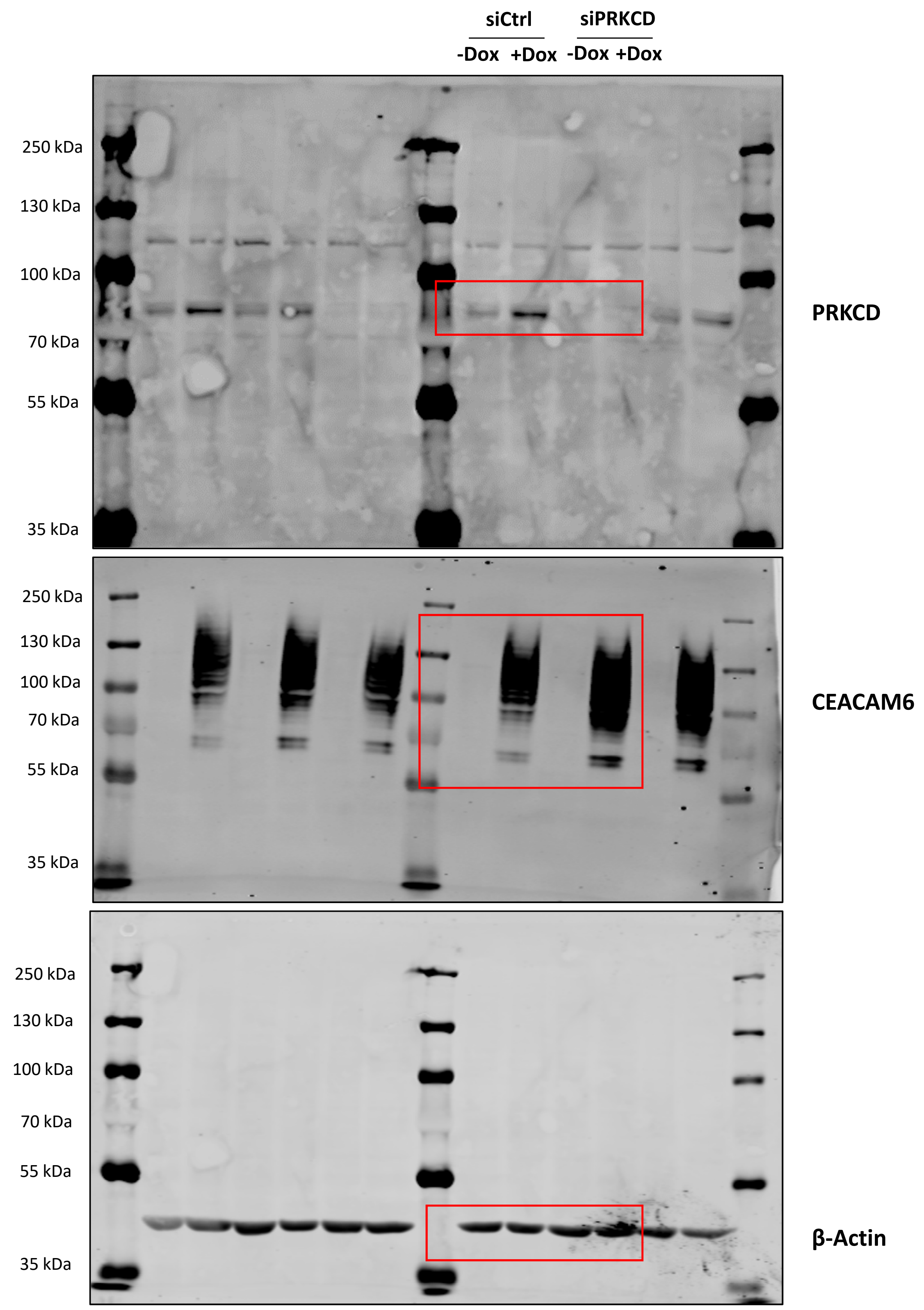

Figure 8C

Capivasertib treatment

GB-d1-CEACAM6

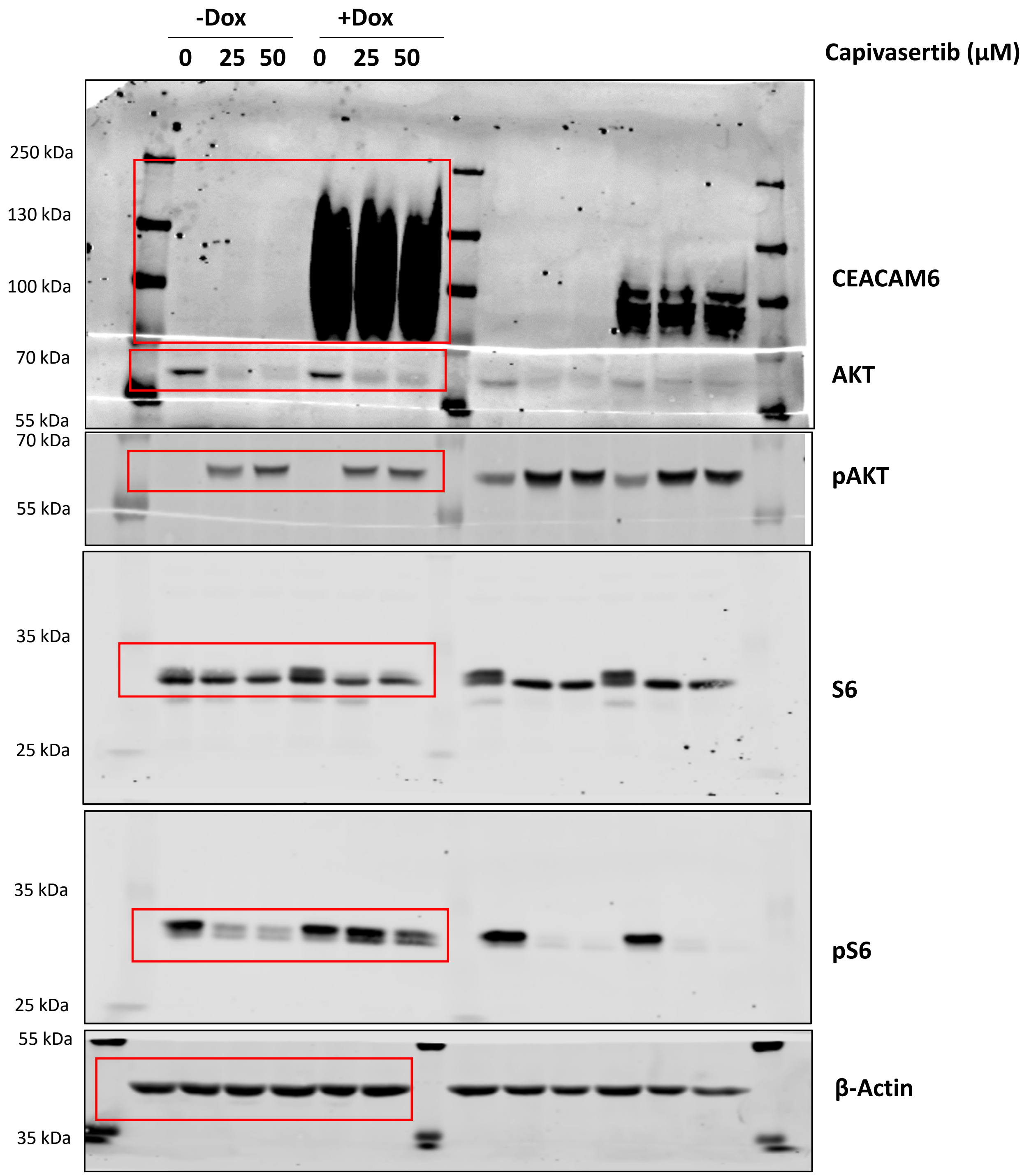

Figure 8E

Ulixertinib treatment

GB-d1-CEACAM6

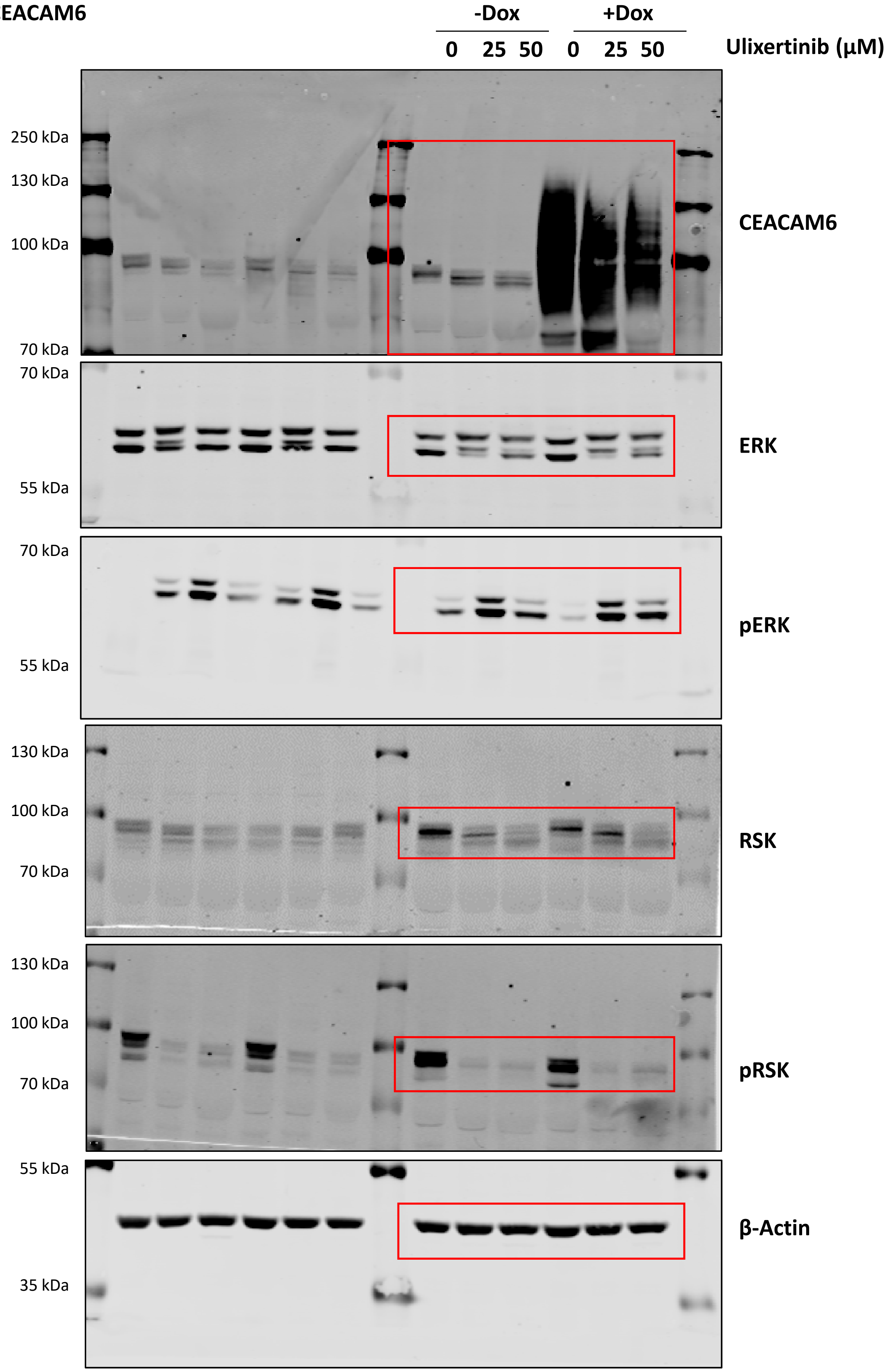

Figure S1H

CEACAM6 overview

GBC cell lines

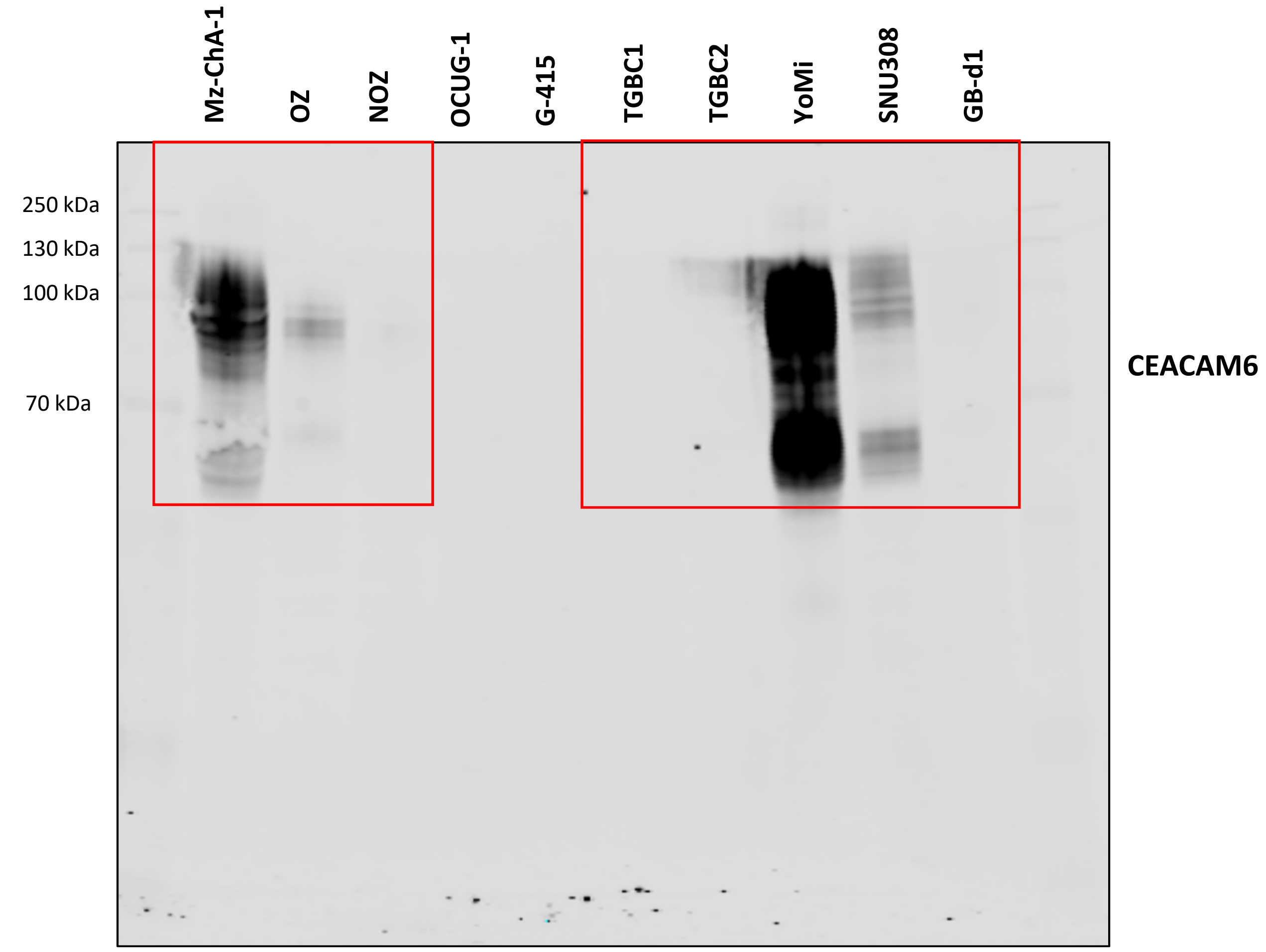

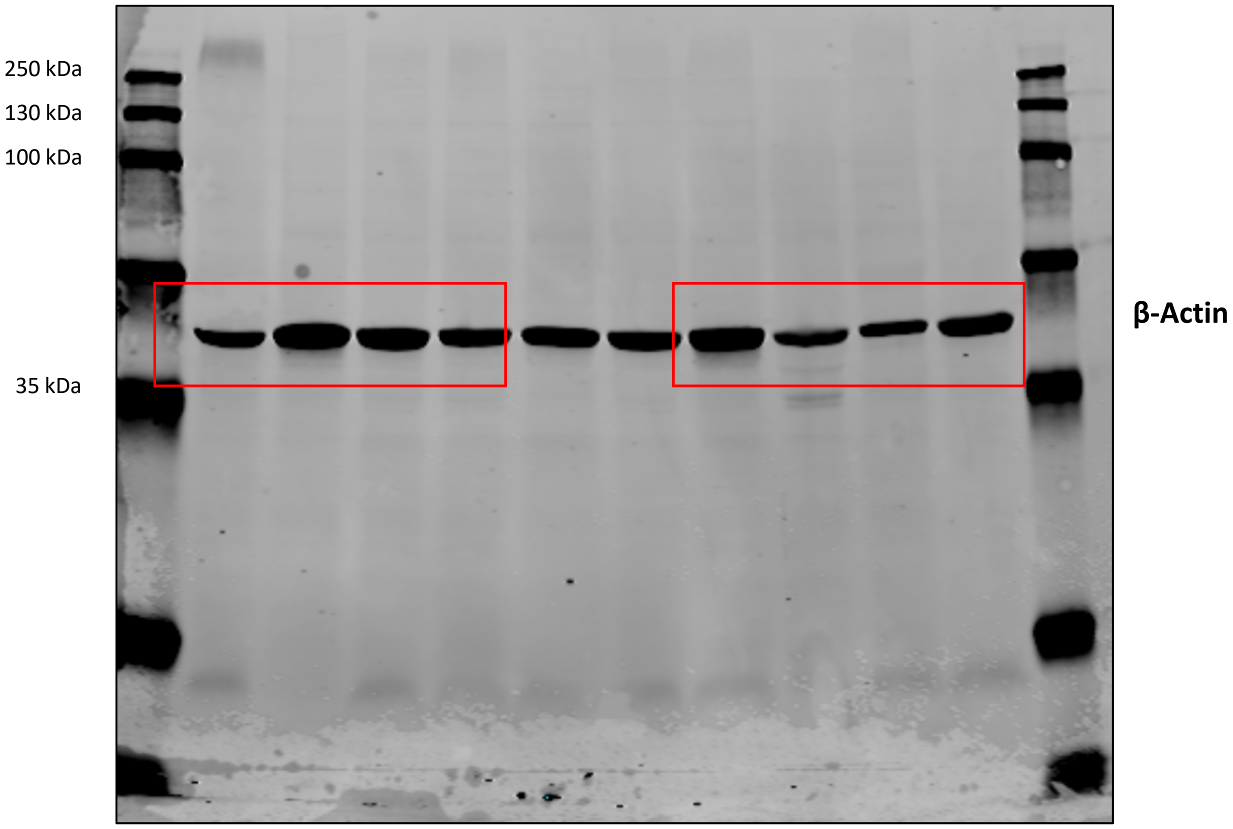

Figure S2H

PARP cleavage after siCEACAM6

Mz-ChA-1

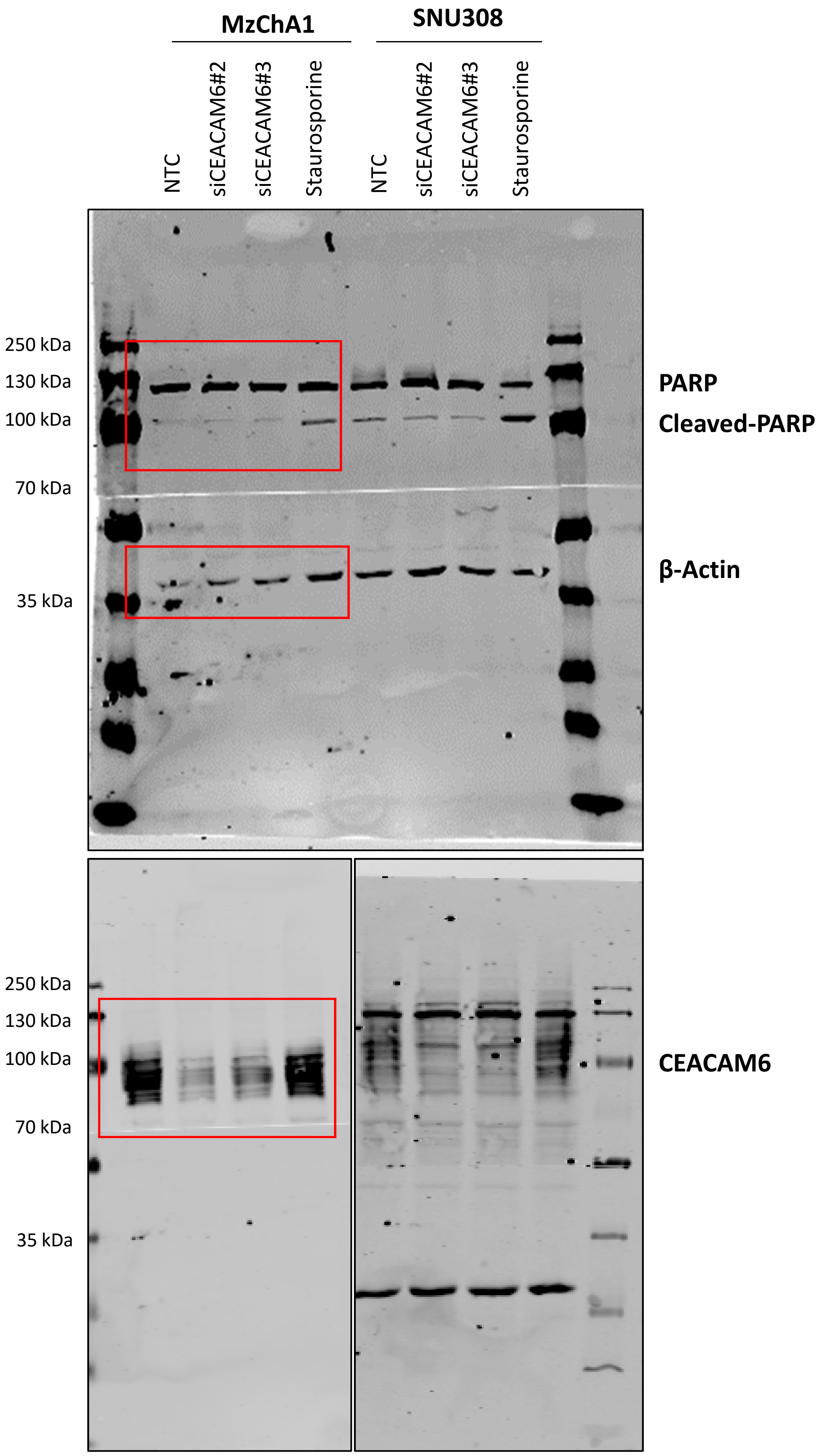

Figure S2J

P16 expression after siCEACAM6

Mz-ChA-1 and SNU308

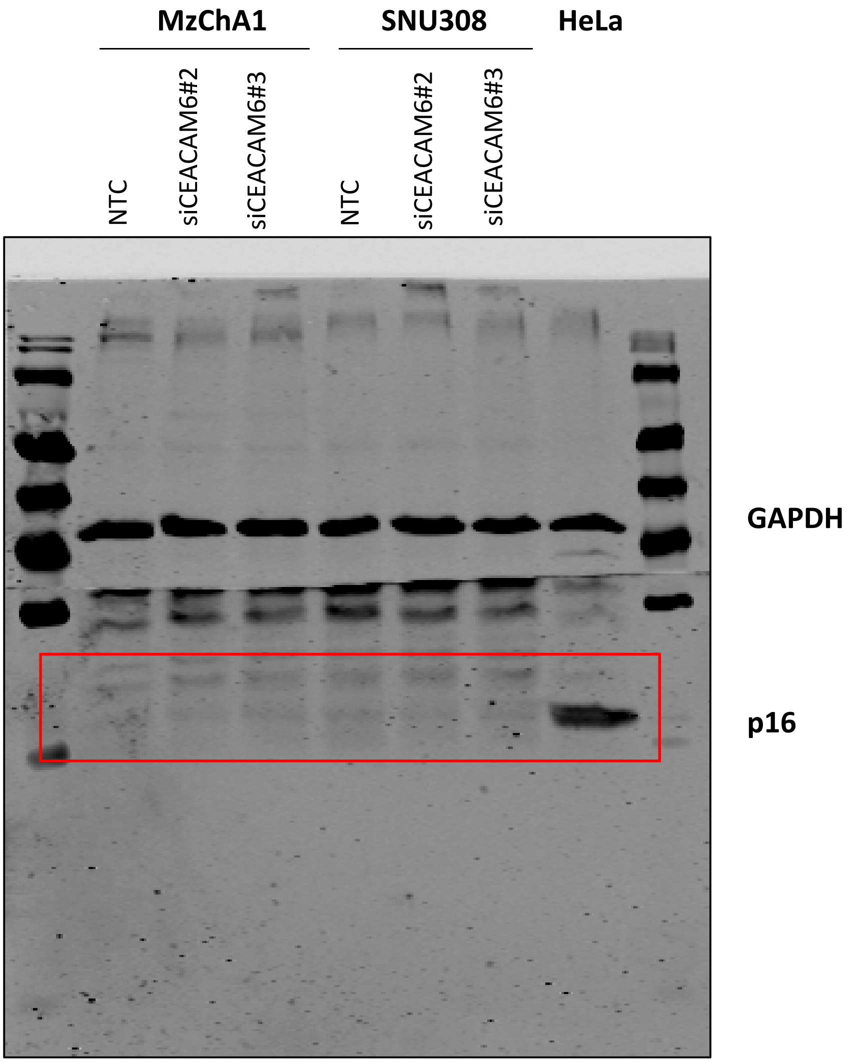

Figure S3C

CEACAM6 Overexpression

TGBC1

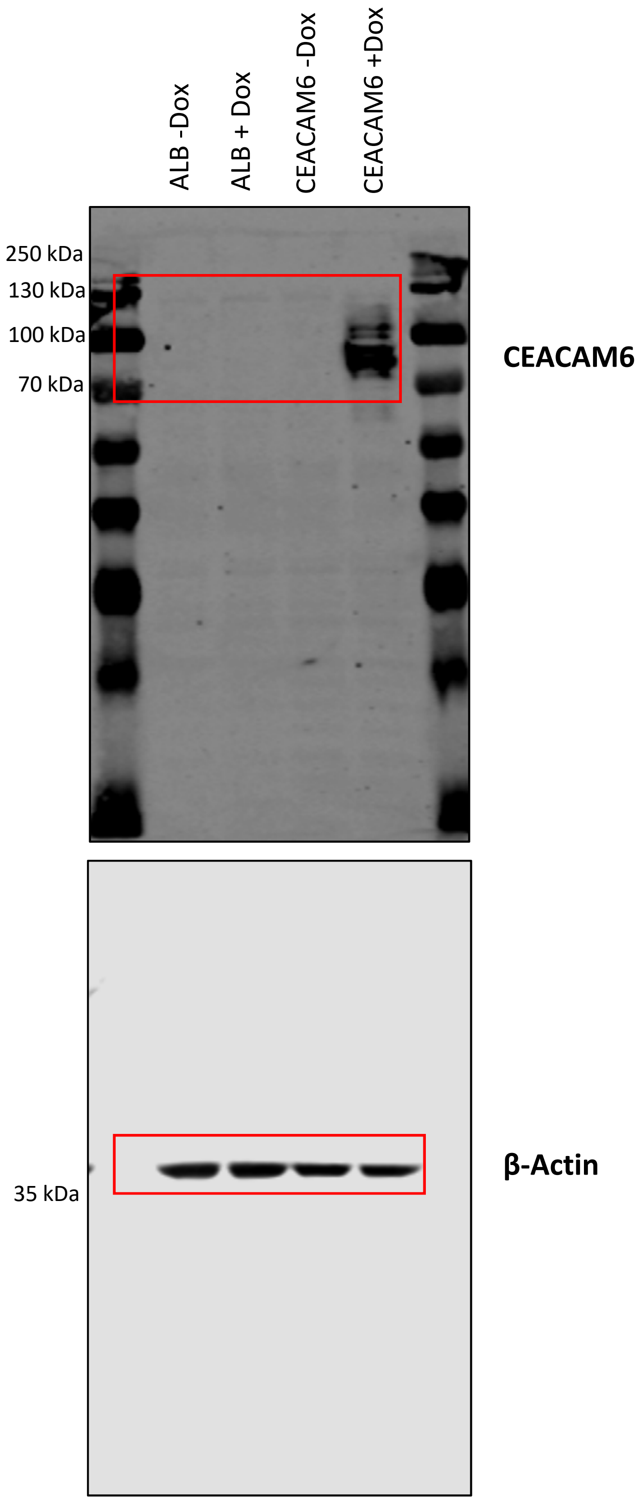

Figure S3I

CEACAM6 Overexpression

GB-d1-GFP-Luc

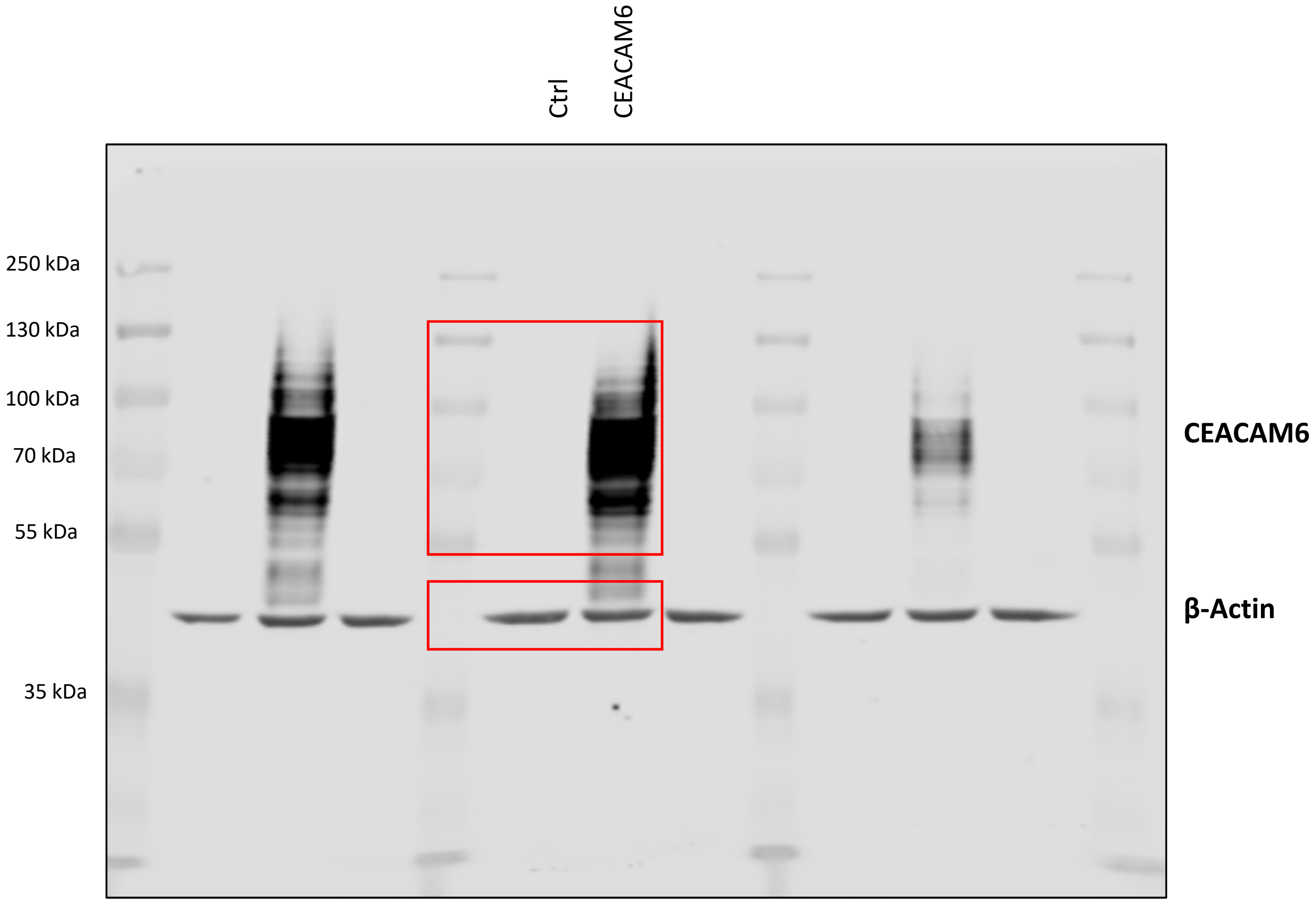

Figure S4B

BioID

GB-d1

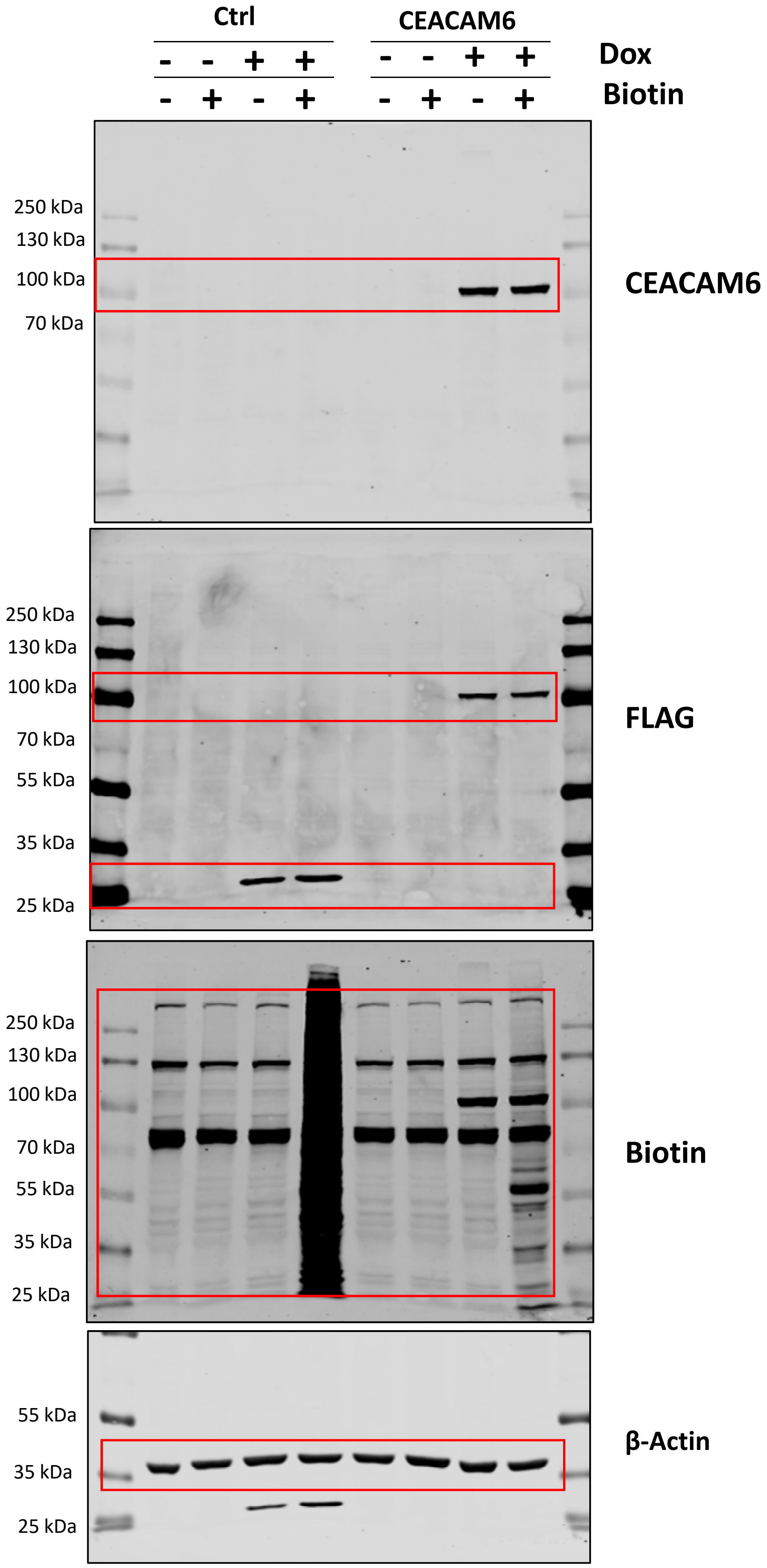

Figure S4C

BioID

GB-d1

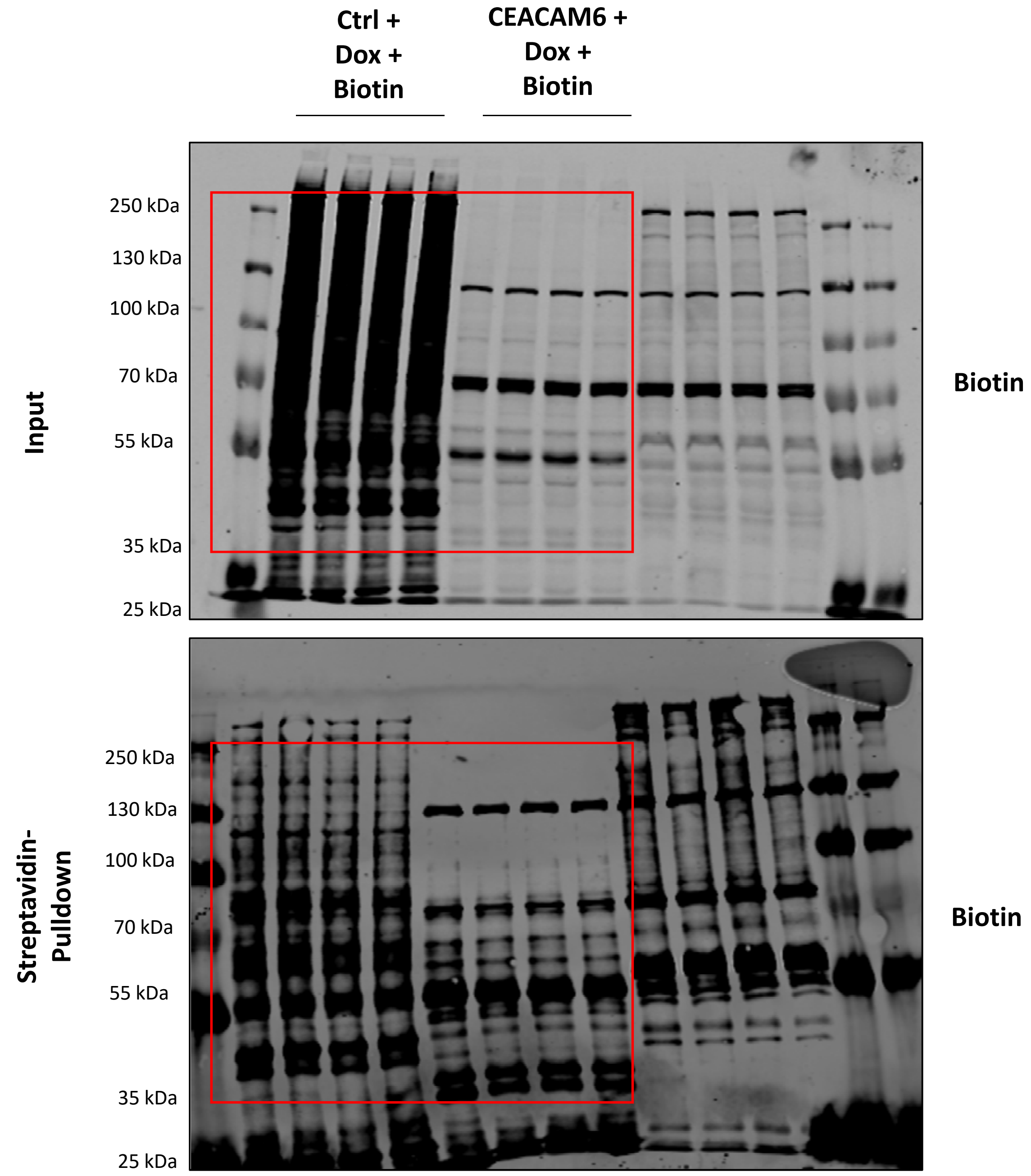

Figure S8A

siITGA2

TGBC1-CEACAM6

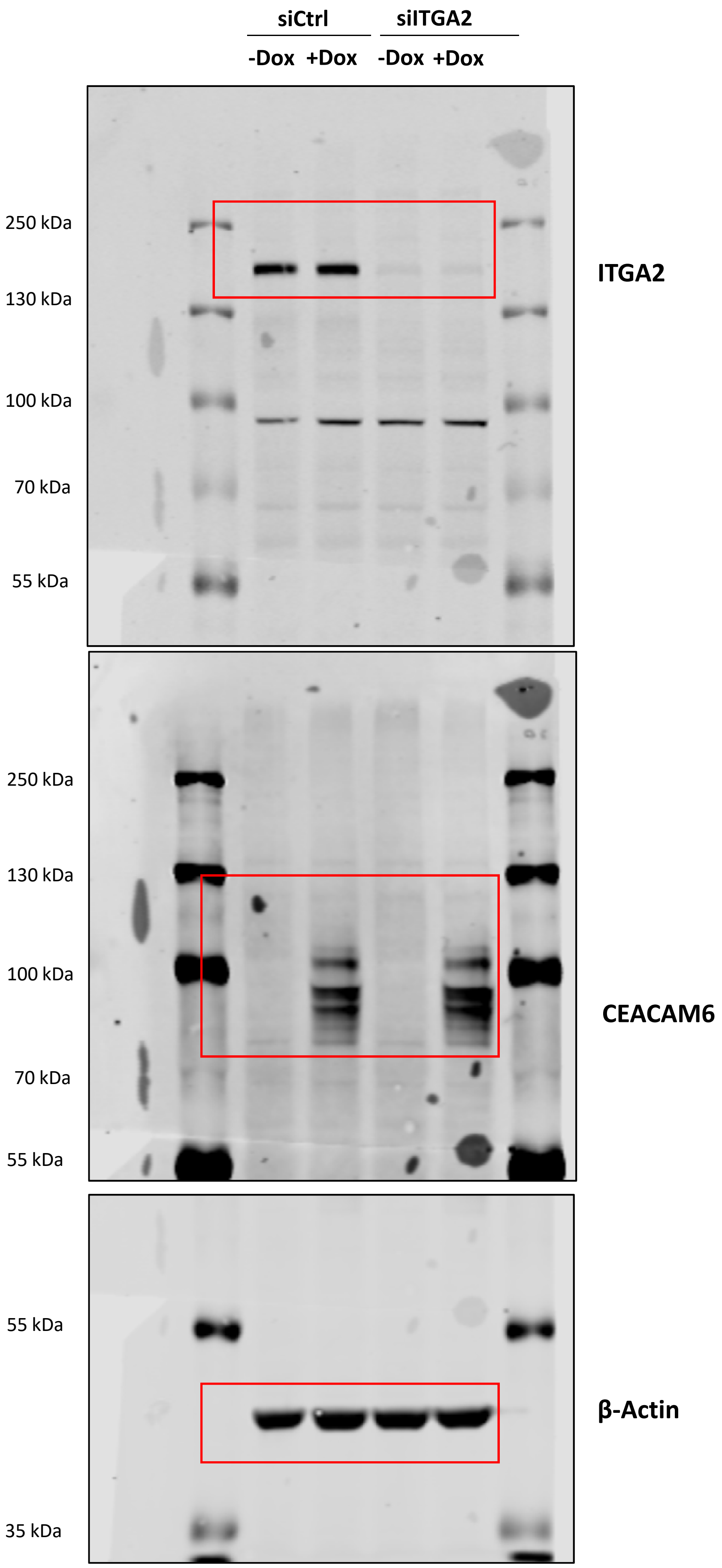

Figure S8B

siITGB1

TGBC1-CEACAM6

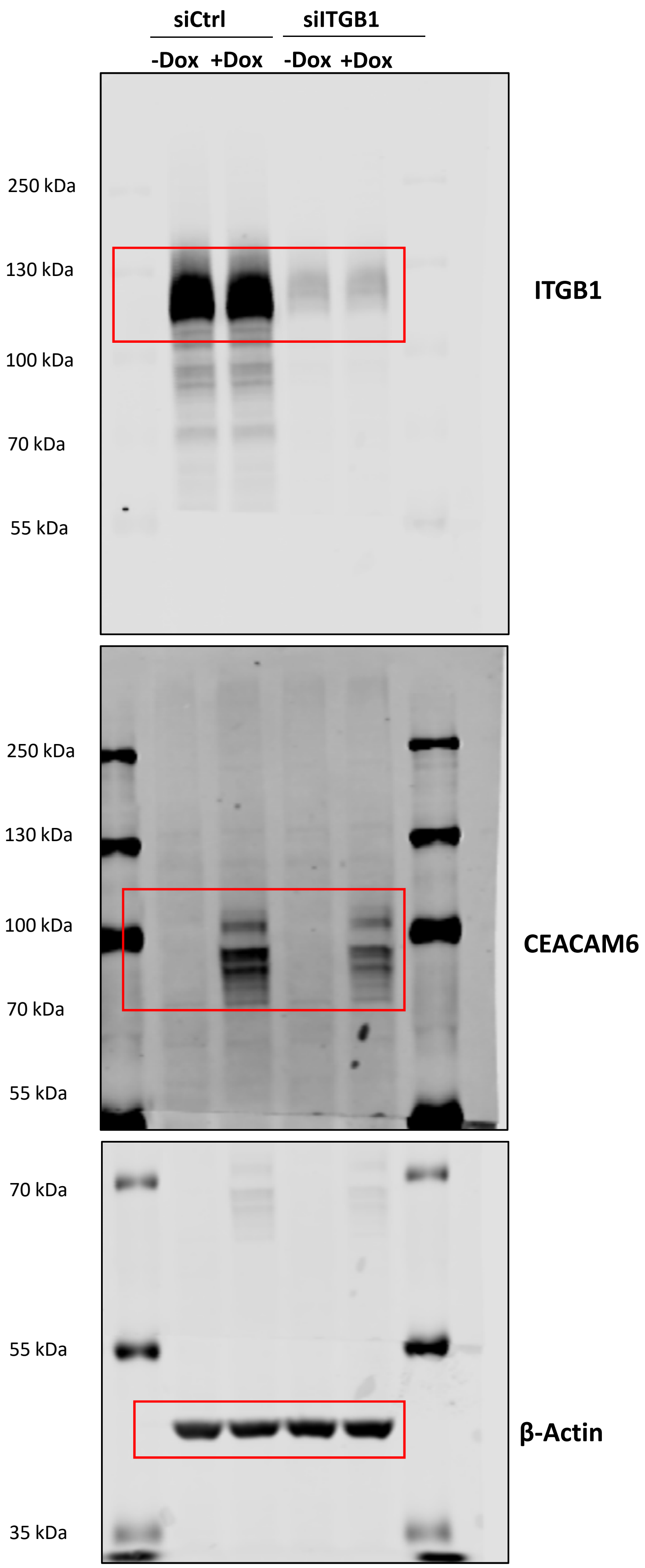

Figure S8C

siITGA2+siITGB1

TGBC1-CEACAM6

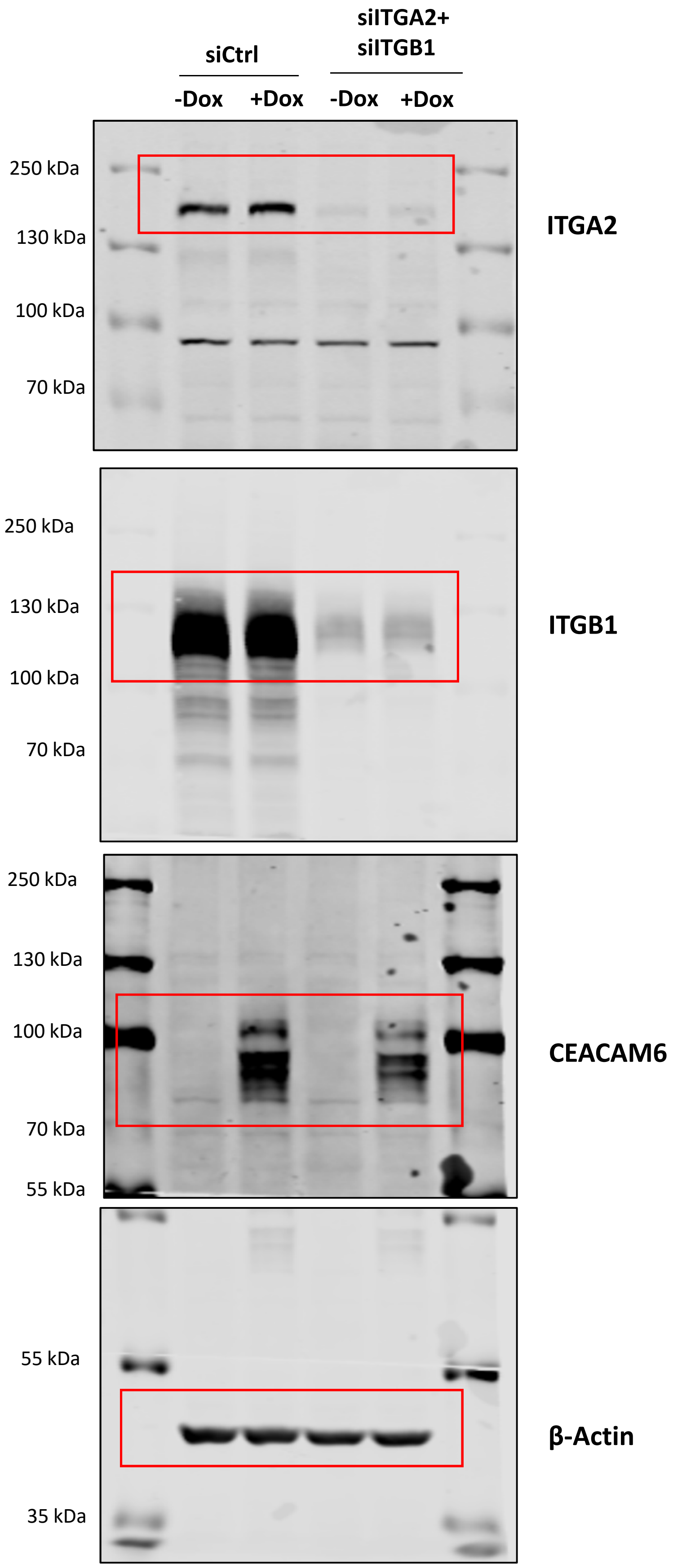

Figure S8D

siIPRKCD

TGBC1-CEACAM6

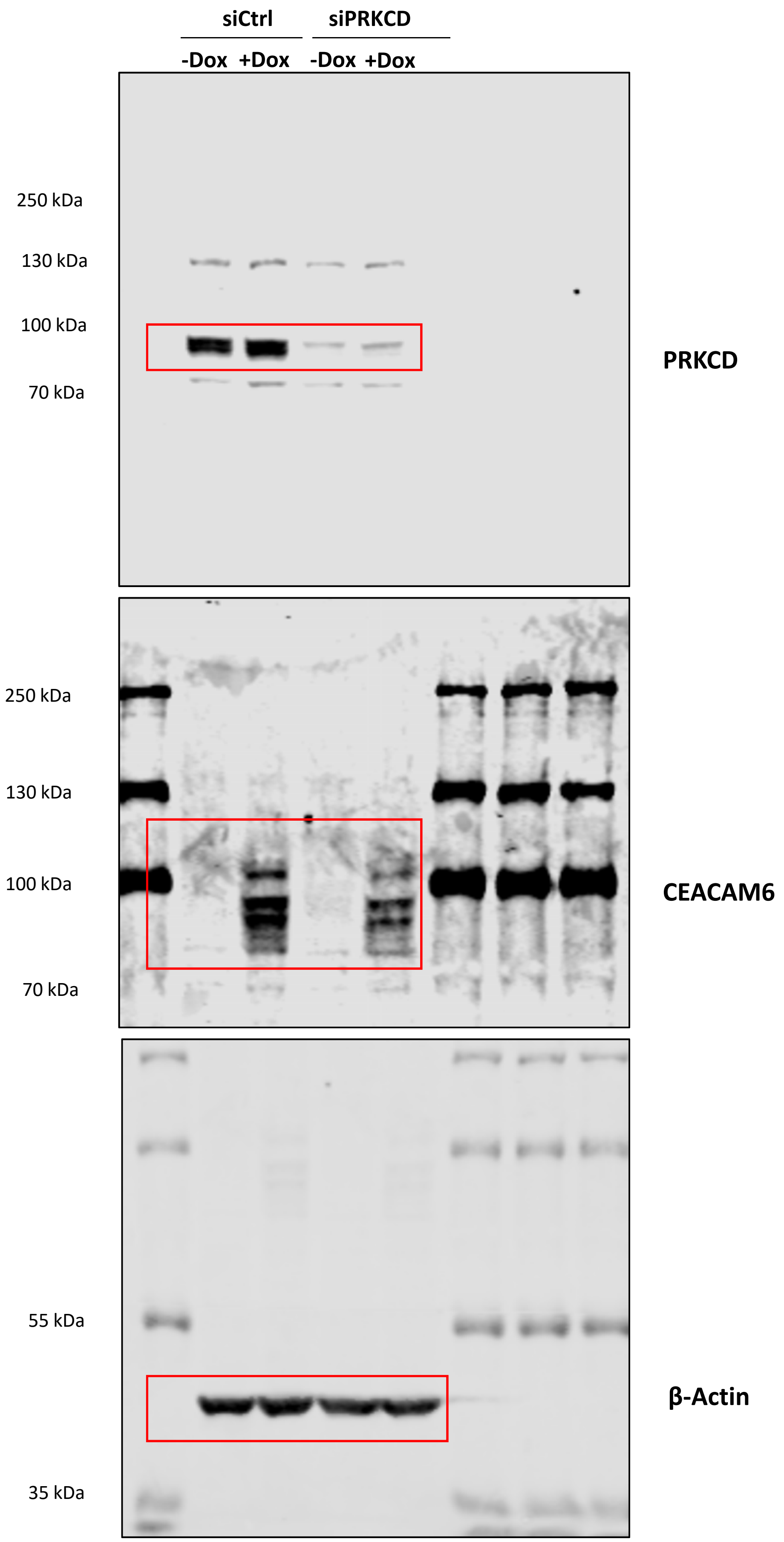

Figure S9E

Capivasertib treatment

TGBC1-CEACAM6

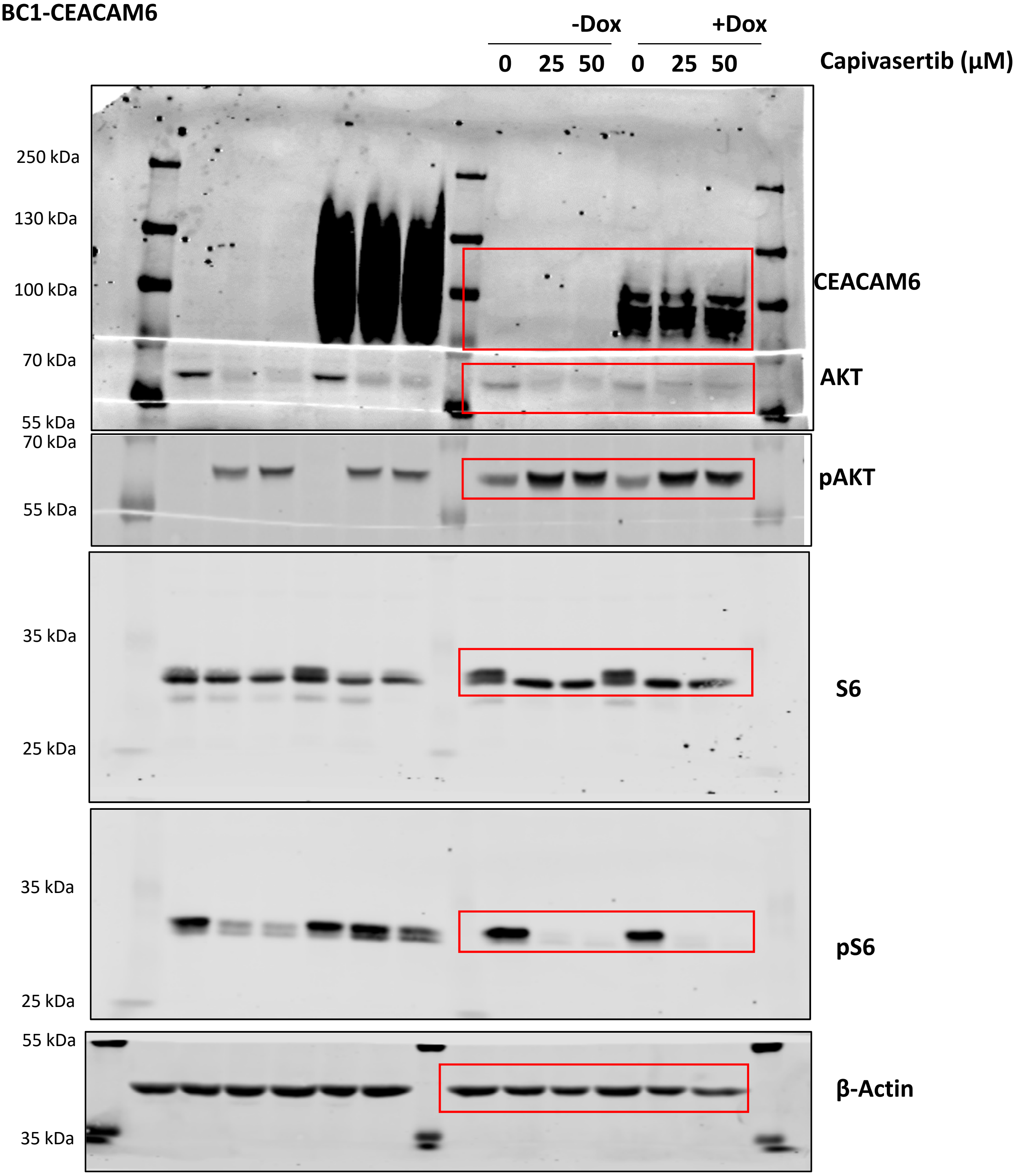

Figure S9G

Ulixertinib treatment

TGBC1-CEACAM6

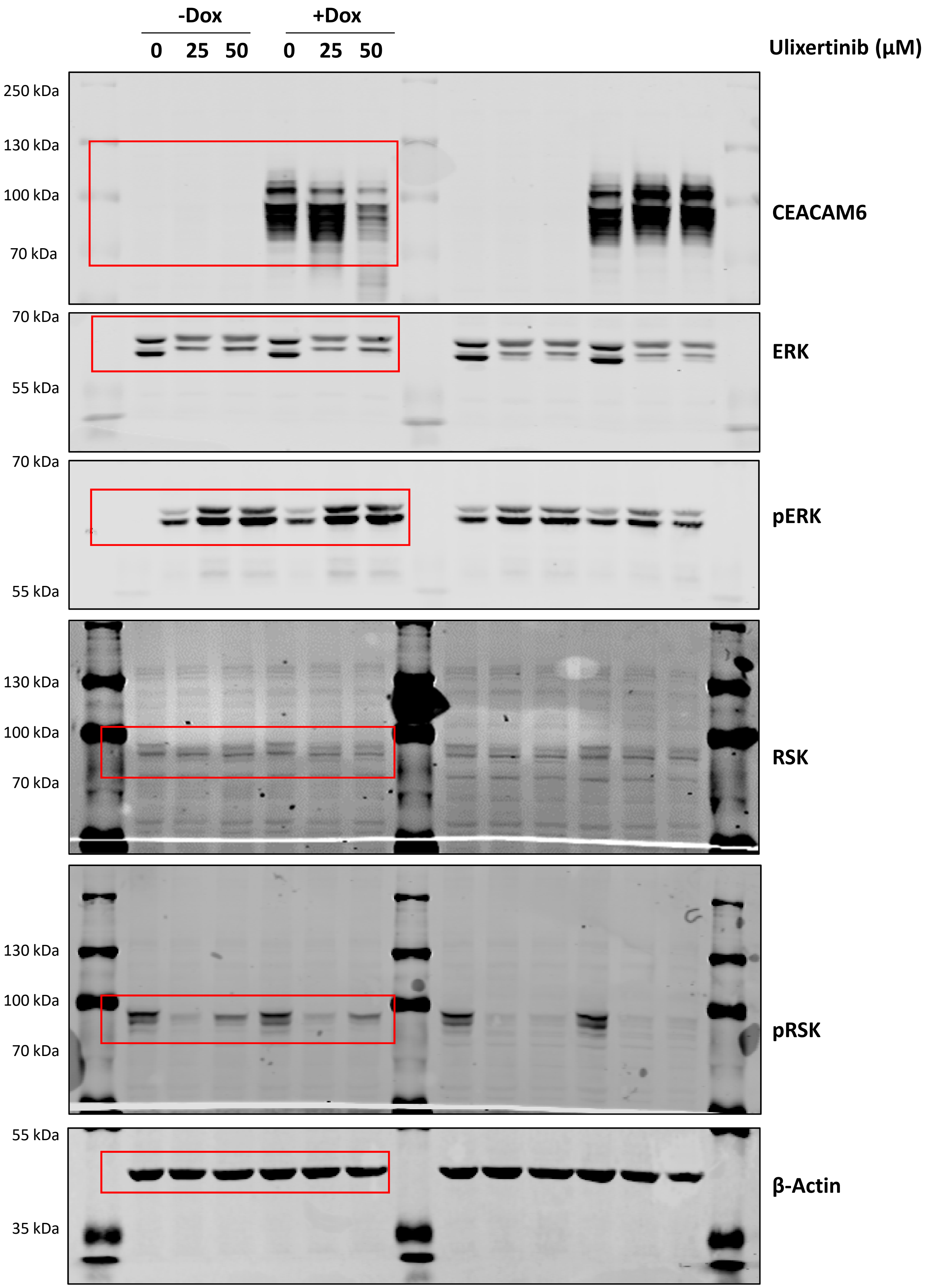

Figure S10A

Capivasertib treatment

Mz-ChA-1 and SNU308

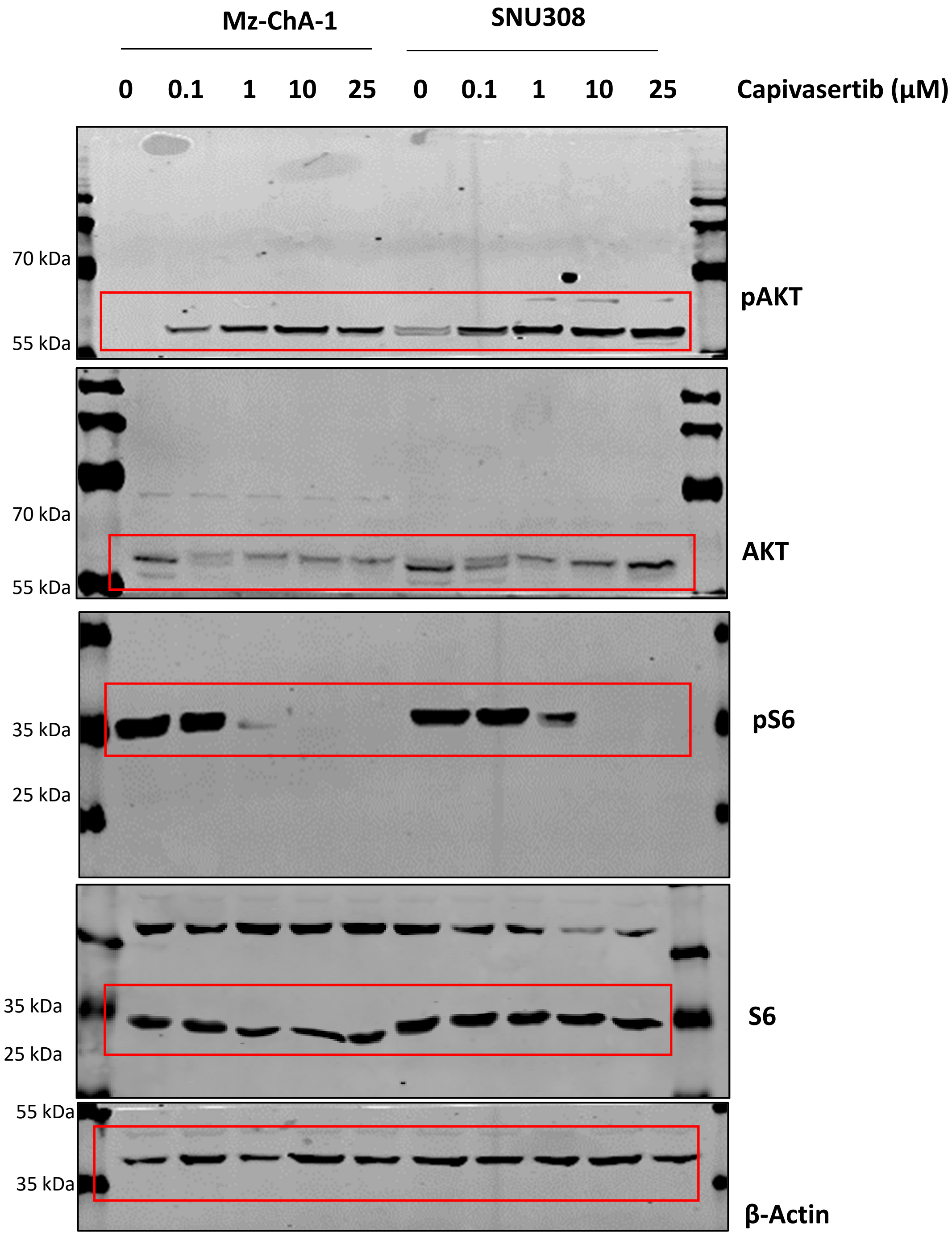

Figure S10A

Capivasertib treatment

GB-d1-CEACAM6

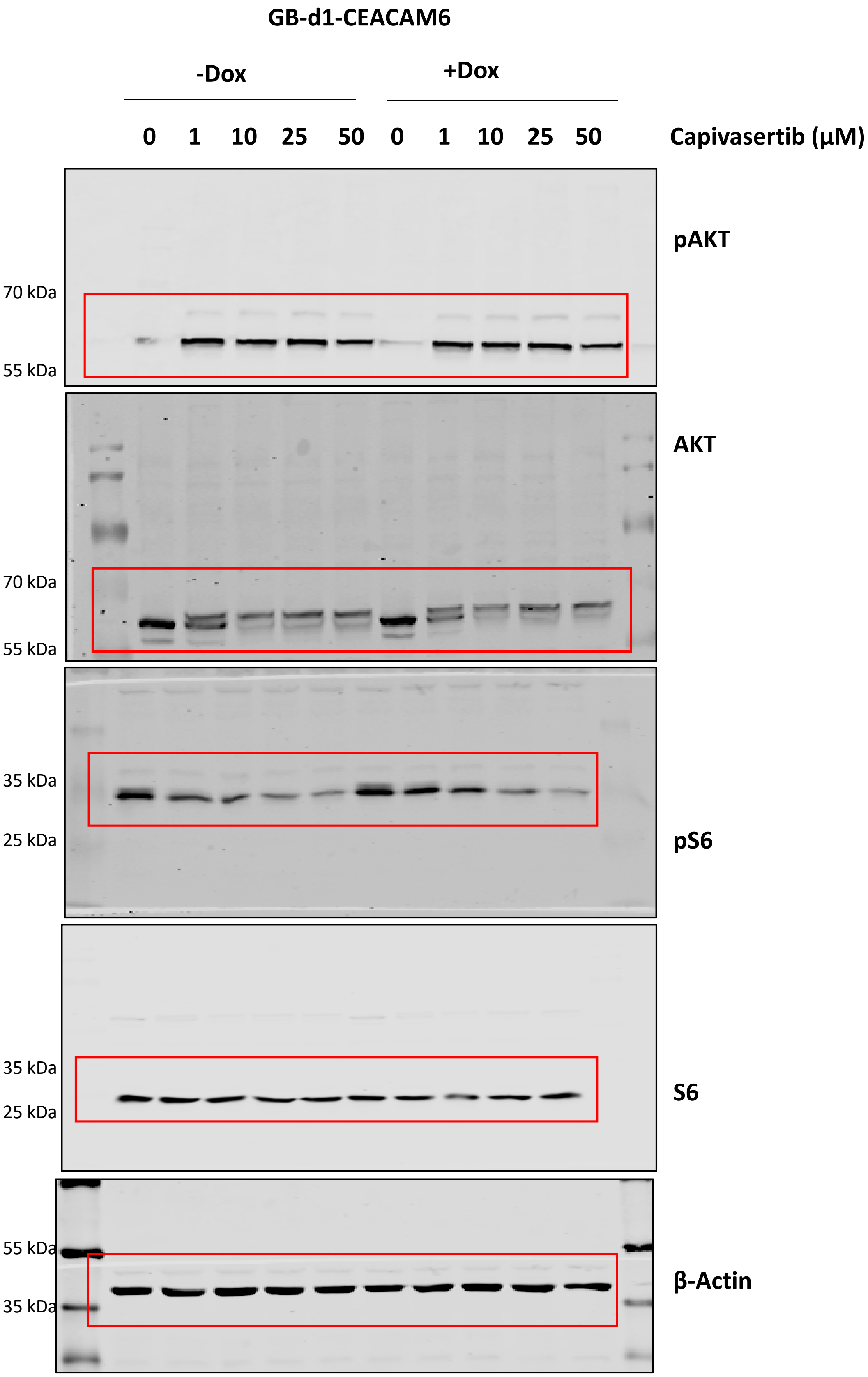

Figure S10A

Capivasertib treatment

TGBC1-CEACAM6

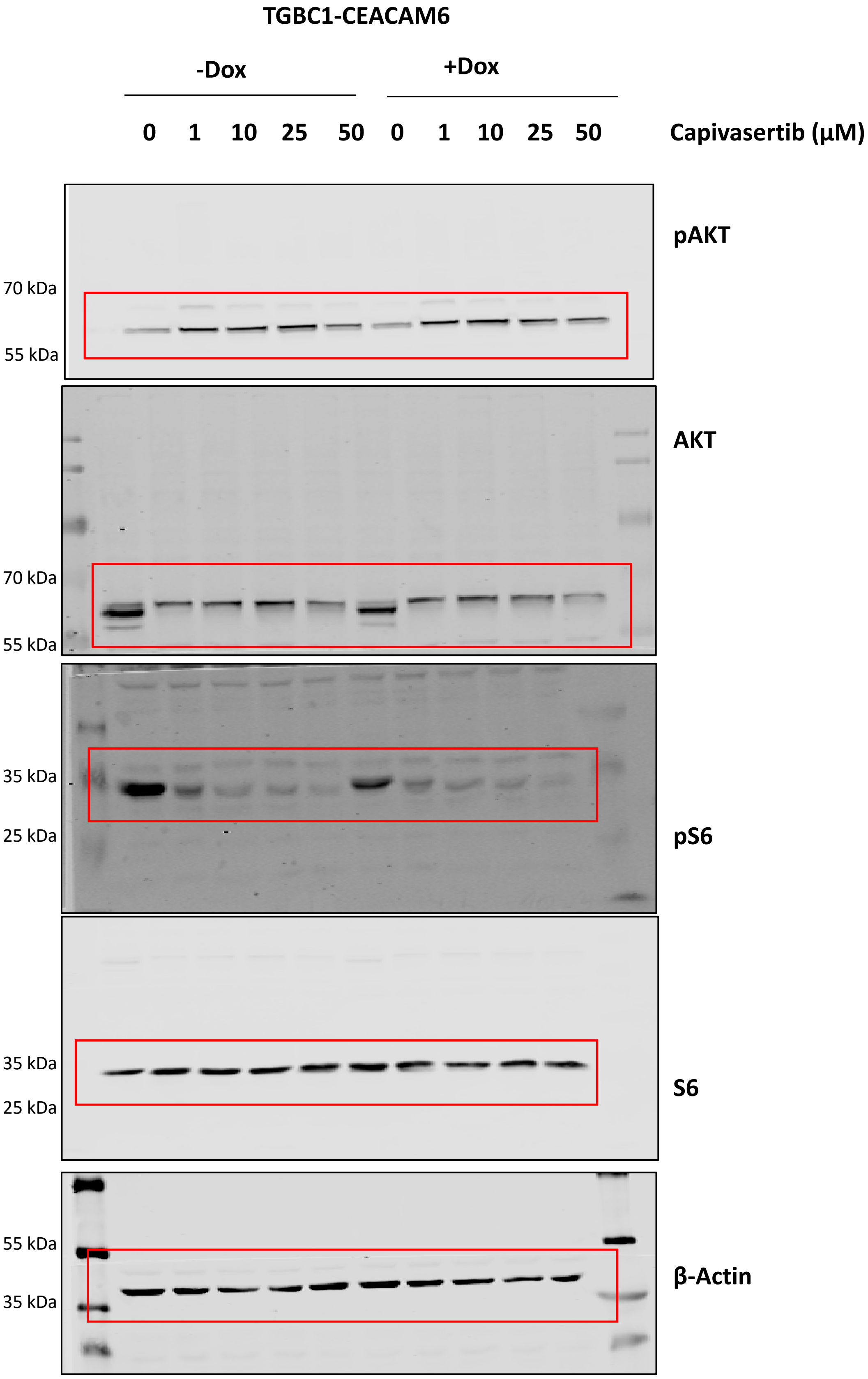

Figure S10B

Ulixertinib treatment

Mz-ChA-1 and SNU308

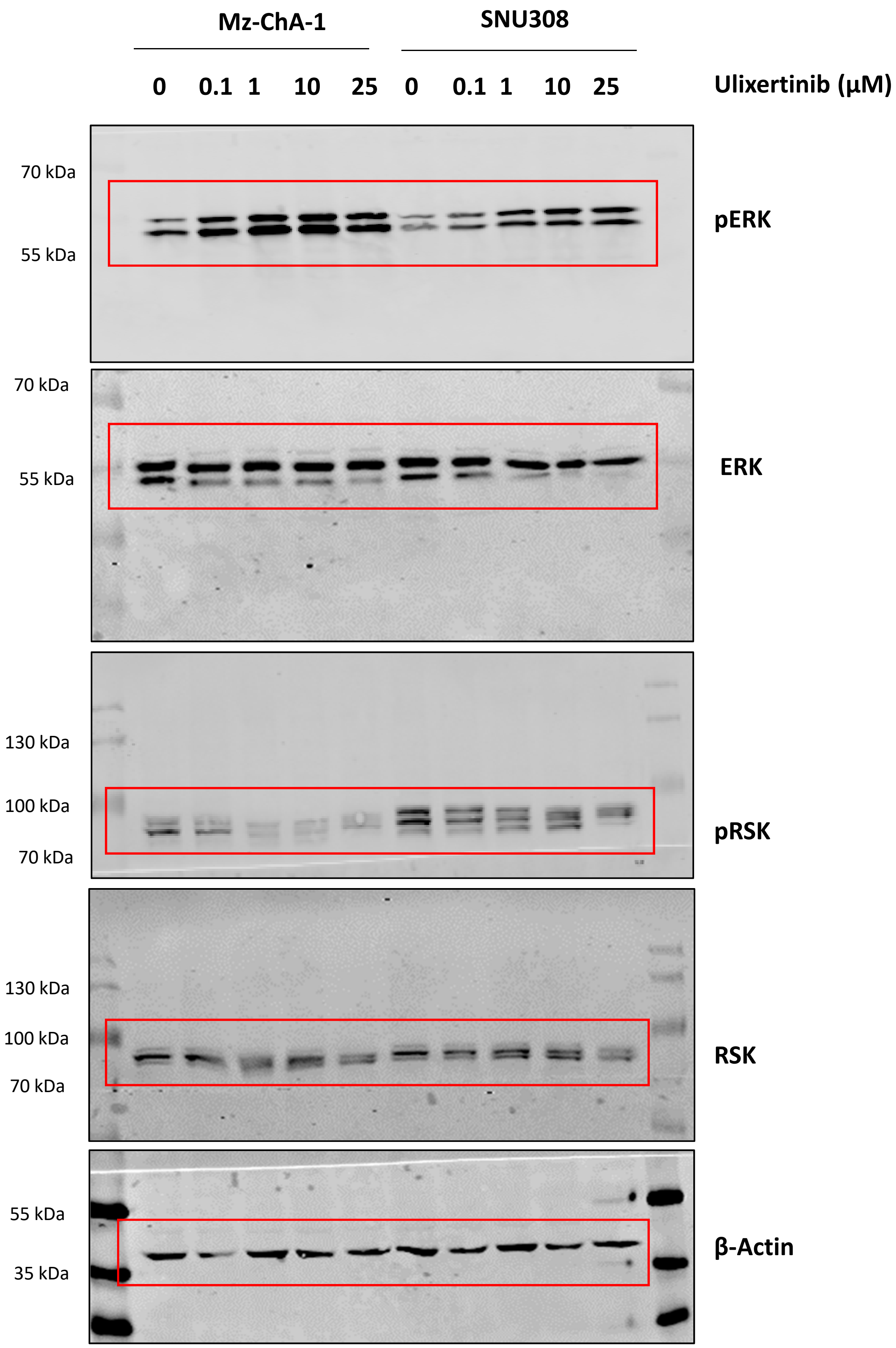

Figure S10B

Ulixertinib treatment

GB-d1-CEACAM6

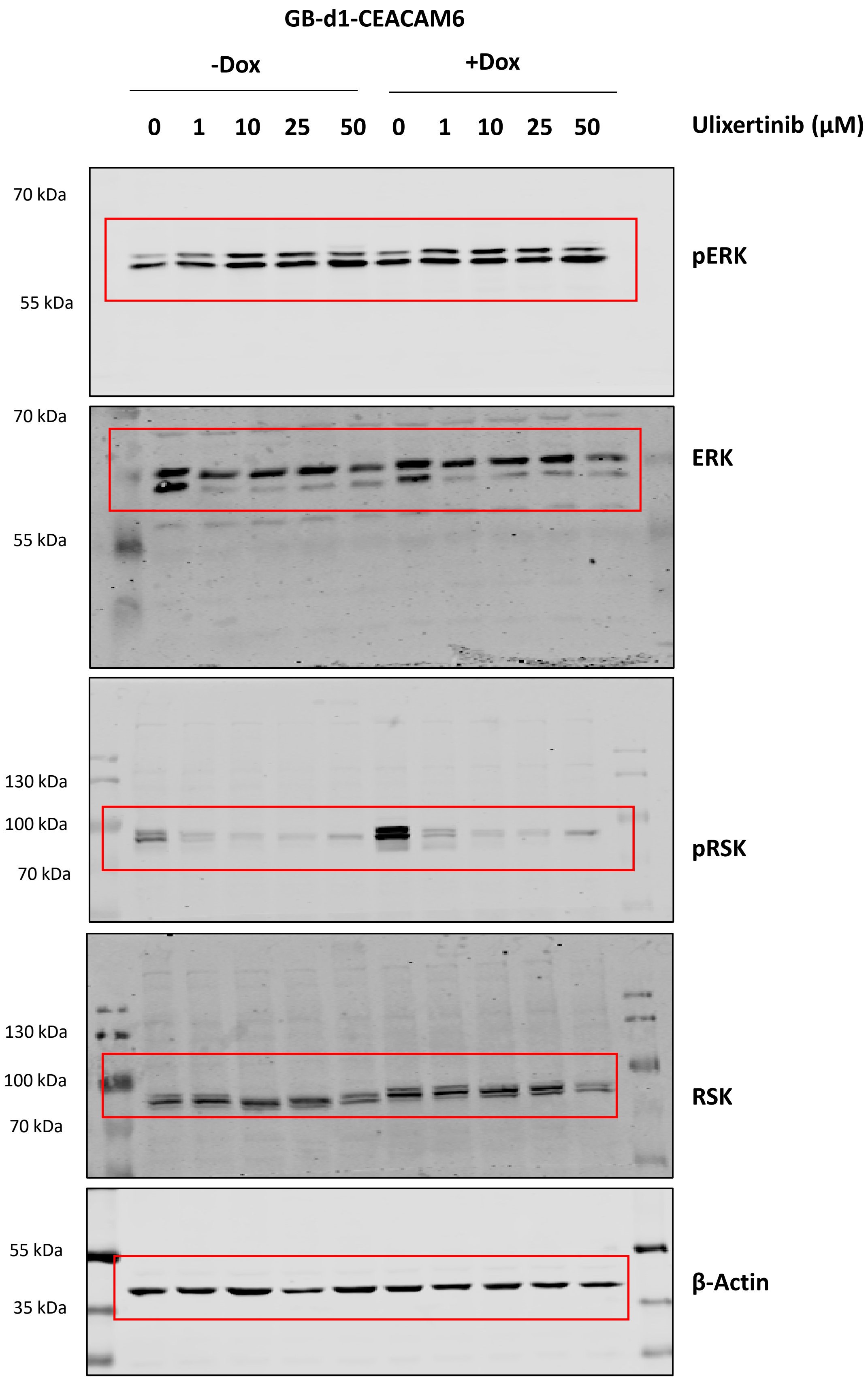

Figure S10B

Ulixertinib treatment

TGBC1-CEACAM6

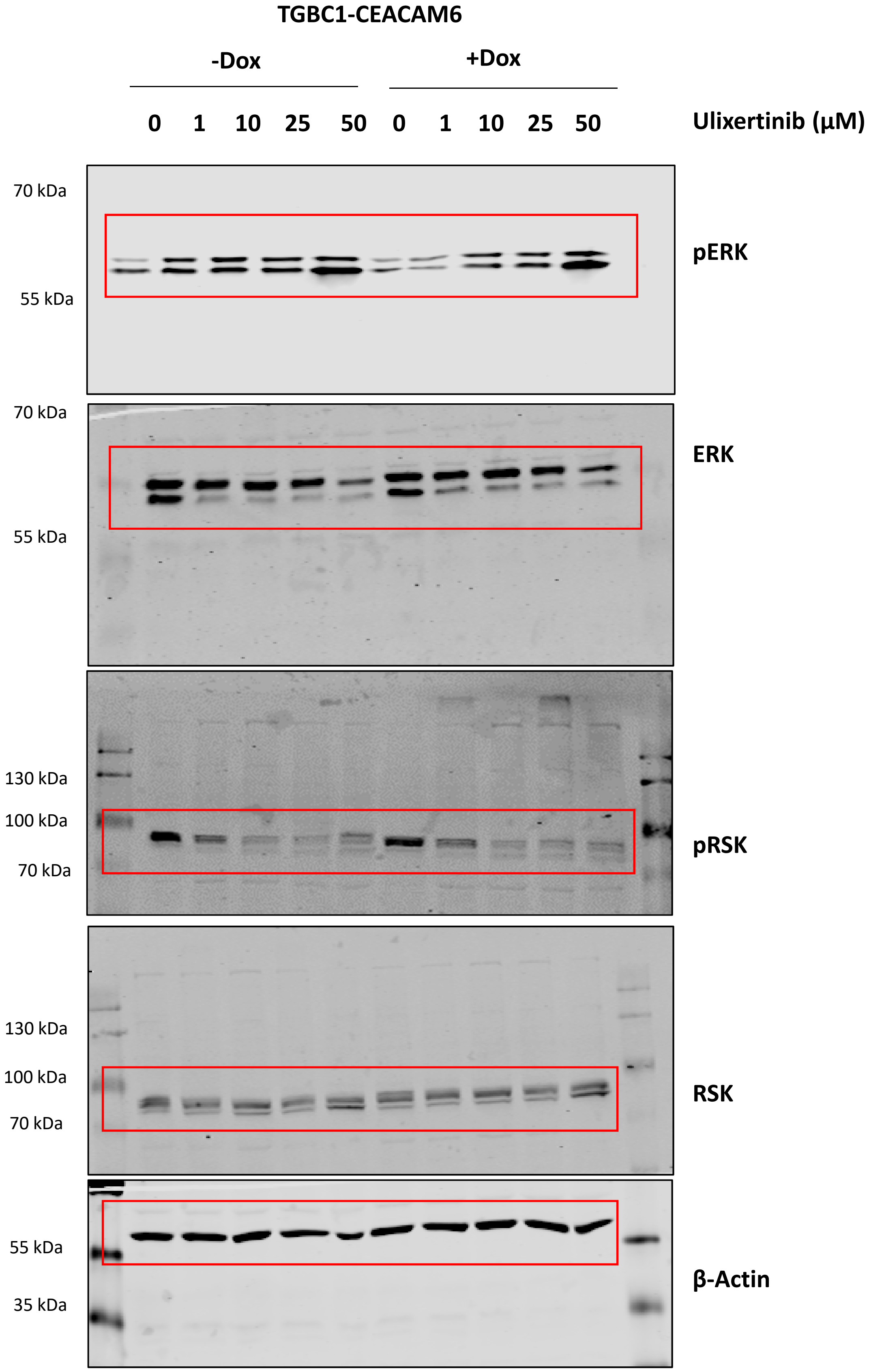

Figure S12F

CEACAM5 expression

GBC cell lines

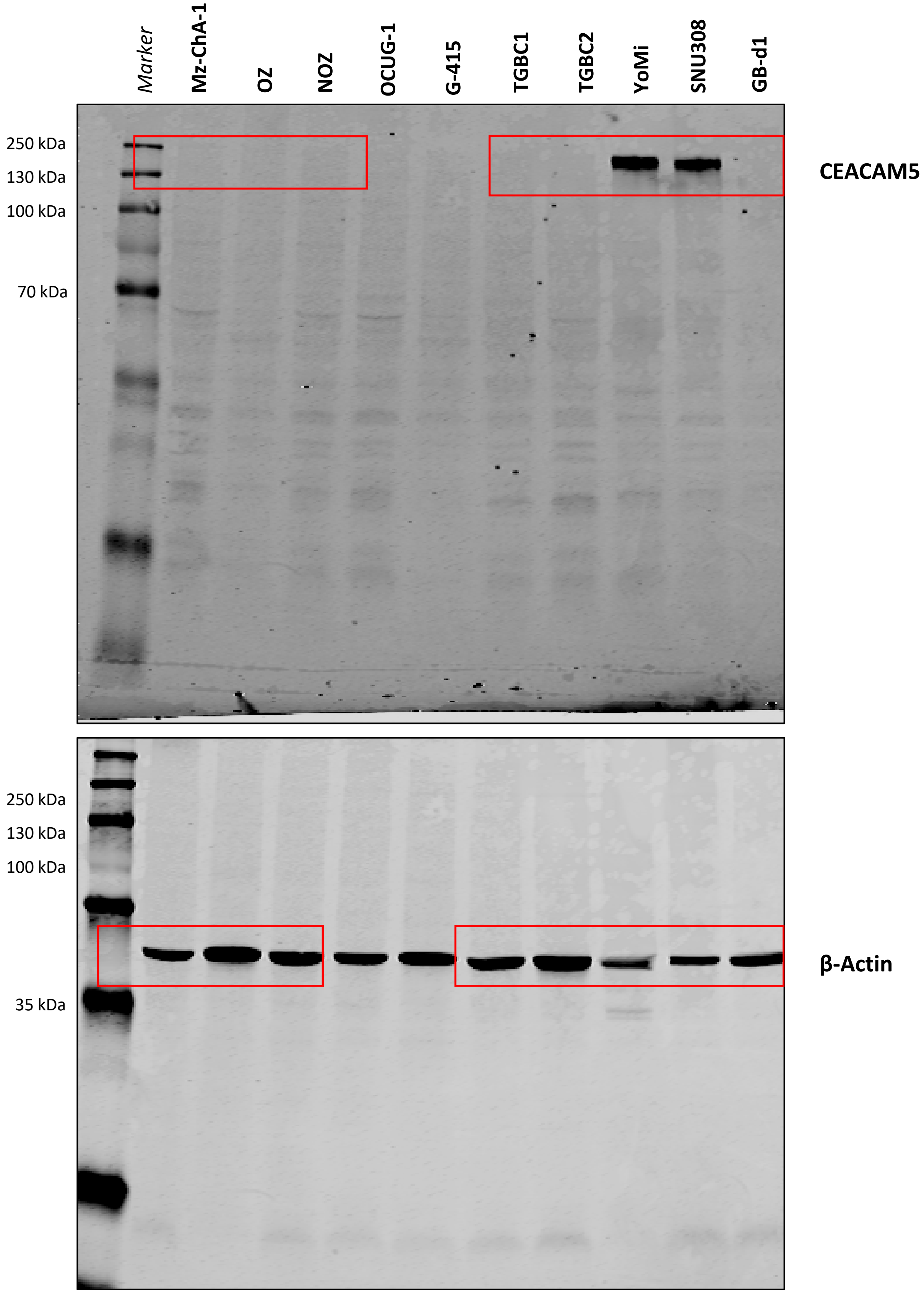

Figure S12F
CEACAM5 knockdown
SNU308

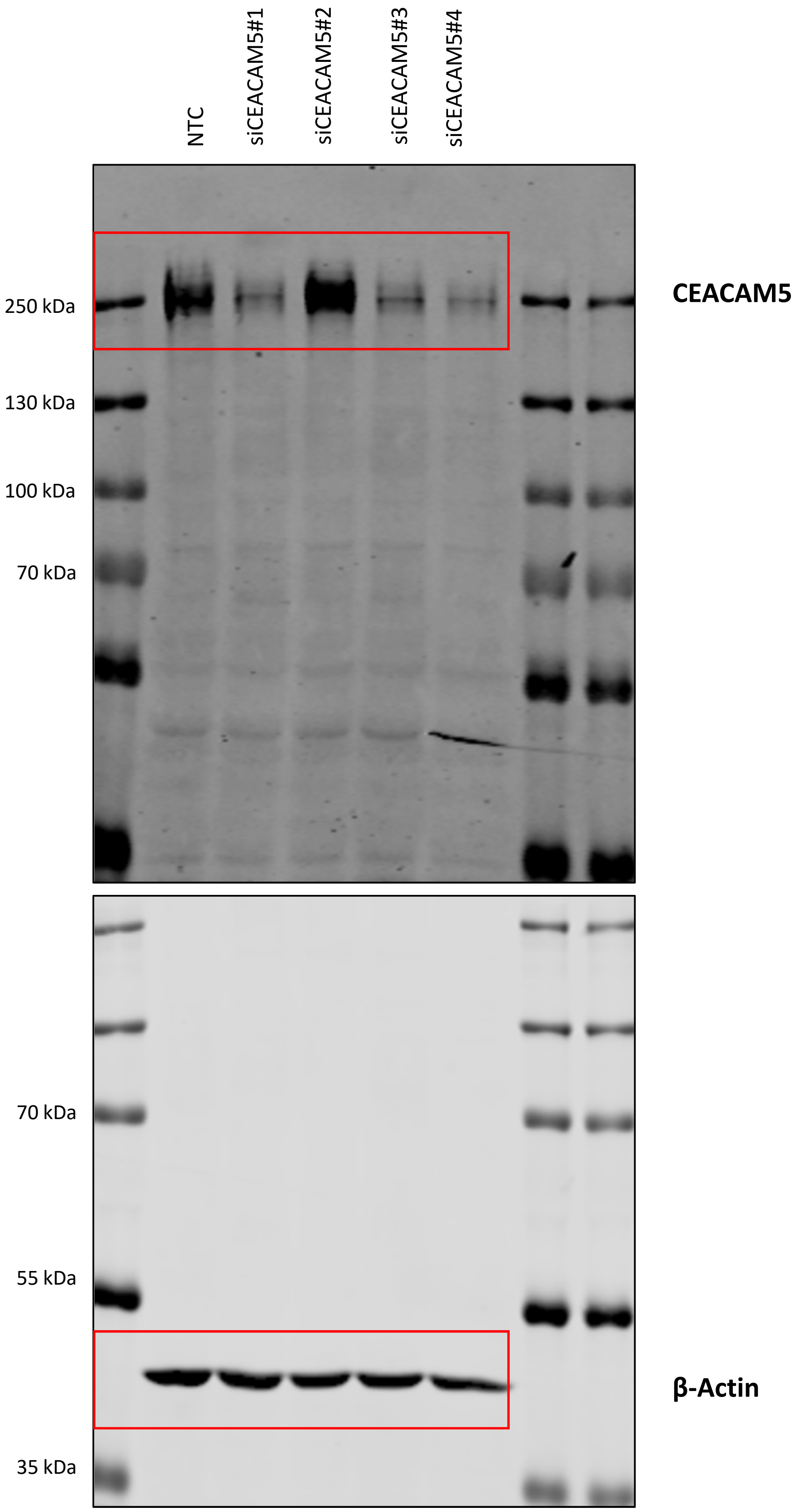

Supplement: Supplementary file 2 — Western blot images [file 41419_2024_7171_MOESM2_ESM.pdf]
